# Genome-wide methylation analysis identifies ELOVL5 as an epigenetic biomarker for the risk of type 2 diabetes mellitus

**DOI:** 10.1038/s41598-018-33238-9

**Published:** 2018-10-05

**Authors:** Joo-Yeon Hwang, Hyo Jung Lee, Min Jin Go, Han Byul Jang, Nak-Hyun Choi, Jae Bum Bae, Juan E. Castillo-Fernandez, Jordana T. Bell, Tim D. Spector, Hye-Ja Lee, Bong-Jo Kim

**Affiliations:** 1 Center for Genome Science, National Institute of Health, Osong Health Technology Administration Complex, Chungcheongbuk-do, Republic of Korea; 2 Center for Biomedical Science, National Institute of Health, Osong Health Technology Administration Complex, Chungcheonbuk-do, Republic of Korea; 3 Department of Twin Research & Genetic Epidemiology, King’s College London, London, SE1 7EH UK

## Abstract

Genome-wide DNA methylation has been implicated in complex human diseases. Here, we identified epigenetic biomarkers for type 2 diabetes (T2D) underlying obesogenic environments. In a blood-based DNA methylation analysis of 11 monozygotic twins (MZTW) discordant for T2D, we discovered genetically independent candidate methylation sites. In a follow-up replication study (17 MZTW pairs) for external validation, we replicated the T2D-association at a novel CpG signal in the ELOVL fatty acid elongase 5 (*ELOVL5*) gene specific to T2D-discordant MZTW. For concordant DNA methylation signatures in tissues, we further confirmed that a CpG site (cg18681426) was associated with adipogenic differentiation between human preadipocytes and adipocytes isolated from the same biopsy sample. In addition, the *ELOVL5* gene was significantly differentially expressed in adipose tissues from unrelated T2D patients and in human pancreatic islets. Our results demonstrate that blood-derived DNA methylation is associated with T2D risk as a proxy for cumulative epigenetic status in human adipose and pancreatic tissues. Moreover, *ELOVL5* expression was increased in cellular and mouse models of induced obesity-related diabetes. These findings may provide new insights into epigenetic architecture by uncovering methylation-based biomarkers.

## Introduction

Type 2 diabetes mellitus (T2D) is a metabolic disease characterized by persistent hyperglycemia and insulin resistance in individuals with obesogenic environmental triggers^1^. T2D is also strongly associated with obesity in adults. In fact, most patients with T2D are overweight or obese^2^. Adipose tissue is implicated in systemic insulin sensitivity, insulin resistance, and diabetes risk^3^.

To date, genome-wide association studies (GWAS) have identified many T2D susceptibility loci. A systematic meta-analysis investigated the role of obesity-associated loci in the development of T2D. These pleiotropic variants are associated with obesity-independent or obesity-mediated T2D risk^4^. To address unresolved issues concerning the etiology of T2D, integrative epigenetic approaches are considered an important strategy^5–7^.

Currently, epigenome-wide association studies (EWAS) have identified epigenetic markers for phenotypic consequences underlying cumulative environmental changes using disease-discordant monozygotic twin (MZTW) models^8–12^. In particular, an epigenomic analysis of the blood-derived DNA methylome demonstrated T2D-associated genetically independent differentially methylated regions (giDMRs) in MZTW^13^. In addition, Ling *et al*. studied epigenetic variations involved in oxidative phosphorylation and insulin secretion in human pancreatic islets^14–17^, including DNA methylation-based biomarkers^18^. However, comprehensive EWAS of T2D in blood and tissues have not been fully explored. In this study, we carried out a multi-stage methylation analysis to identify corresponding epigenetic biomarkers across blood and tissues. We also investigated *ELOVL5* expression in human liver cells and in tissues from mice with induced obesity-related diabetes.

## Results

### Discovery of giDMRs in T2D-discordant MZTW pairs

**Figure 1:**
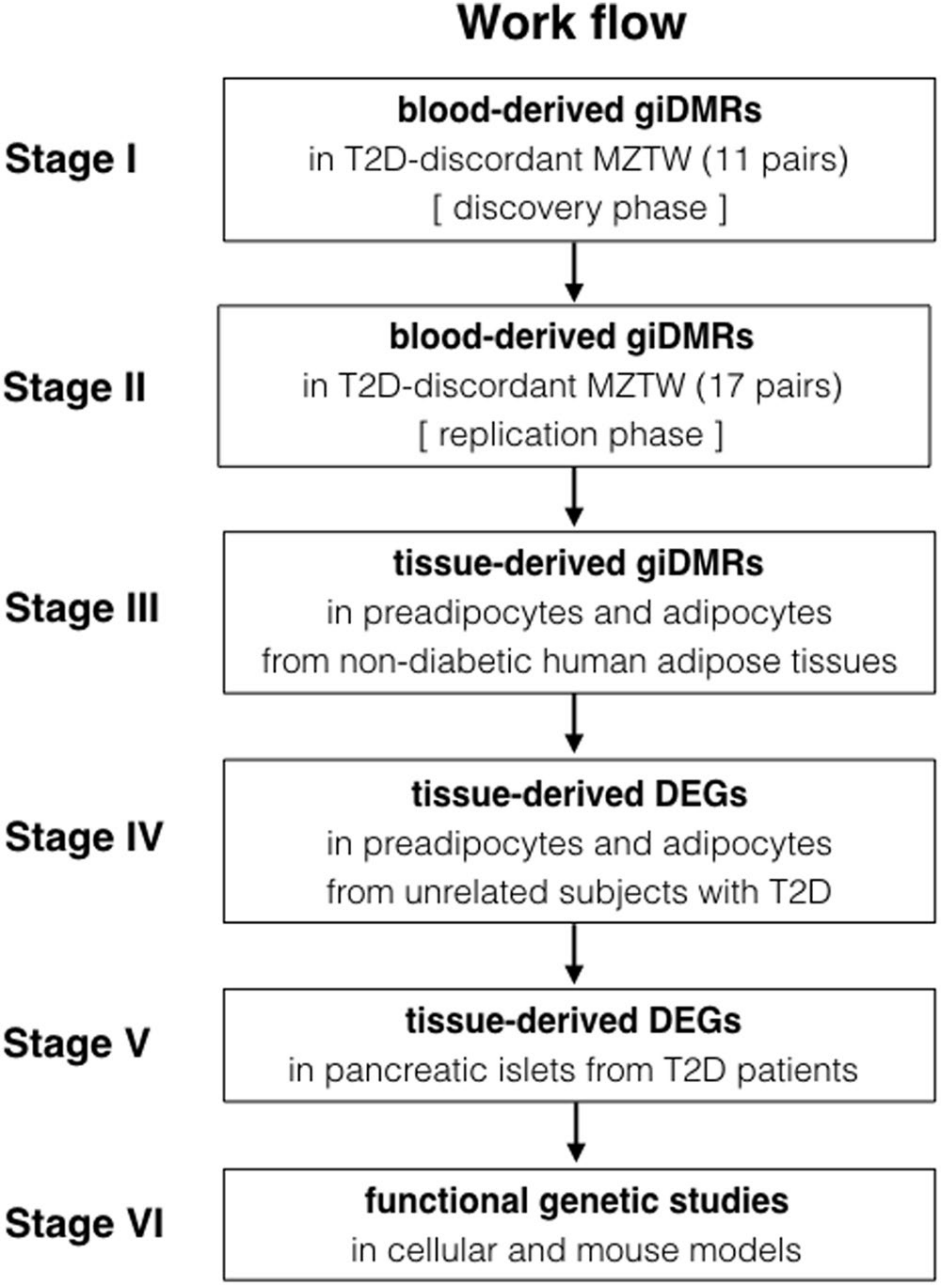
Study design and overall work flow for the analysis pipeline.

This study was conducted with the approval from the appropriate institutional review board, and all participants provided written informed consent. The flowchart shown in Fig. 1 and Supplementary Data 1 and 2 summarize the overall study design for a multi-stage EWAS of T2D. Descriptive characteristics and statistics of T2D-discordant MZTW pairs are outlined in Table S1. In the discovery stage, we detected putative blood-derived epigenetic differences within genetically identical twins using a one-sample parametric *t*-test (Fig. S1) with the Illumina Infinium Human Methylation 450 k BeadChip. These giDMRs represent the pure environmental status of the disease itself. In addition, we confirmed the highly-replicated smoking-associated 450 k CpG sites (cg06644428 and cg21566642 in the 2q37.1 region) within our data set (data not shown).

### T2D-giDMR replication in T2D-discordant MZTW pairs

To replicate putative T2D-giDMRs for external validation in an independent ethnic group, we performed an *in silico* search for 16 leading CpG sites using blood-derived methylated DNA immunoprecipitation and high-throughput sequencing (MeDIP-seq) data from 17 T2D-discordant MZTW pairs in the TwinsUK cohort. Of these sites, cg18681426 in the *ELOVL5* gene was only replicated with the same direction of association and with nominal significance (*P* < 0.05; Table 1). In addition, a meta analysis demonstrated straightforward enrichment of associations across the two studies (P_meta_ = 3.697e-04).

**Table 1:** DNA methylation specific to T2D-discordant MZ twins in the discovery and the replication stages.

CHR	MAPINFO	TargetID	Gene	Location	Discovery stage		Replication stage	
					mean.dif	P	mean.dif	P
1	99469819	cg26527487	**LPPR5**	Body	−0.0716	0.0078	−0.8500	0.0031
17	16283976	cg06537829	**UBB**	TSS1500	−0.2170	0.0066	−0.5728	0.0073
4	52943197	cg24395452	SPATA18	Body	0.1015	0.0486	−0.6919	0.0107
6	53174395	cg18681426	**ELOVL5**	5′UTR;Body	−0.0075	0.0102	−0.6708	0.0123
1	64649805	cg08036553	**ROR1**	Intergenic	0.4494	0.0370	0.7339	0.0128
1	32714038	cg11286035	**FAM167B**	Body	−0.2385	0.0003	−0.7814	0.0176
3	142700876	cg00831726	PAQR9	Intergenic	0.7795	0.0396	−0.7331	0.0196
6	169002120	cg14806083	SMOC2	Body	0.9558	0.0347	−0.7905	0.0208
6	21589356	cg20825506	SOX4	Intergenic	−0.2274	0.0295	0.8511	0.0217
6	27841122	cg25845597	**HIST1H4L**	Body	−0.2798	0.0454	−0.5534	0.0221
12	7021987	cg09815977	**LRRC23**	Body	0.9468	0.0079	0.4969	0.0240
7	156813574	cg24052359	MNX1	Intergenic	0.0741	0.0289	−0.5202	0.0332
14	55661413	cg10351052	**DLGAP5**	Intergenic	0.9583	0.0392	0.7630	0.0381
8	41168336	cg23359714	**SFRP1**	Intergenic	−0.2461	0.0435	−0.8940	0.0382
3	38496096	cg01465620	**ACVR2B**	Body	−0.0141	0.0448	−0.4999	0.0404
18	77201558	cg18108009	**NFATC1**	Body	0.6736	0.0232	0.6492	0.0504

### T2D-giDMR is associated with epigenetic changes in adipose tissue

To elucidate the impact of blood-derived epigenetic differences on human tissues, we further evaluated the CpG site in *ELOVL5* and its relationship to altered DNA methylomic profiles between preadipocytes and adipocytes isolated from adipose tissue from the same individual (Fig. S2). Characteristics of the non-diabetic human adipose tissue donors are shown in Table S2. The blood-derived T2D-giDMR (cg18681426 in *ELOVL5*) was significantly associated with adipogenic differences using a one-sample parametric *t*-test with the Illumina Infinium Human Methylation 450 k BeadChip data (*P* = 0.0265; Supplementary Data 3 and 4).

### *ELOVL5* is associated with expression changes in adipose tissue from T2D patients

To validate the biological relevance of mRNA expression, we analyzed the expression of *ELOVL5* in adipose tissue from unrelated subjects with T2D (Table S3). We observed that ELOVL5 expression differed significantly between preadipocytes and adipocytes (Figs S3 and 4, Table 2). The transcript variants (type 1–3) of the ELOVL5 gene were significantly higher in the adipocytes. The variant 4 (NM_001242831) lacks several 3′ exons and contains a novel 3′ terminal exon compared to predominant isoform 1 (NM_021824). We further confirmed adipose tissue specific expression marks that are previously known to be overexpressed in preadipocytes and adipocytes^19^ (Tables S4 and 5, Fig. S5).

**Table 2:** ELOVL5 gene expression profiles in preadipocytes and adipocytes from T2D patients.

ID	transcript type	length	adipocyte (n = 7)		pre-adipocyte (n = 6)		*P*
			mean	std dev	mean	std dev	
NM_021814	variant 1	81,782	134.10	51.20	52.27	17.16	0.0050
NM_001242828	variant 2	81,782	1.03	0.79	0.31	0.28	0.0337
NM_001242830	variant 3	81,782	14.48	7.82	1.83	1.26	0.0020
NM_001242831	variant 4	55,092	5.24	3.78	2.45	1.11	0.0522

### *ELOVL5* is correlated with gene expression in human pancreatic islets

To investigate the relationship between DNA methylation and gene expression, we tested expression profiles using publicly accessible resources (GEO accession number: GSE38642) in the Gene Expression Omnibus (GEO) database. The data were from an mRNA expression microarray based on the Affymetrix GeneChip Human Gene 1.0 ST Array. The clinical characteristics of the T2D donors and non-diabetic donors (age-/gender-matched samples, n = 10) are summarized in Table S6. The *ELOVL5* gene was found to be slightly upregulated in pancreatic islets from T2D patients (P = 0.04) (Fig. S6). LASAGNA-Search 2.0 showed no evidence for transcription factor binding sites positionally overlapping with CpG sites in the promoter region of *ELOVL5* (data not shown)^18^. Also, we observed a positive correlation between gene body DNA methylation and expression.

### *ELOVL5* expression is increased in insulin resistance and inflammation

**Figure 2:**
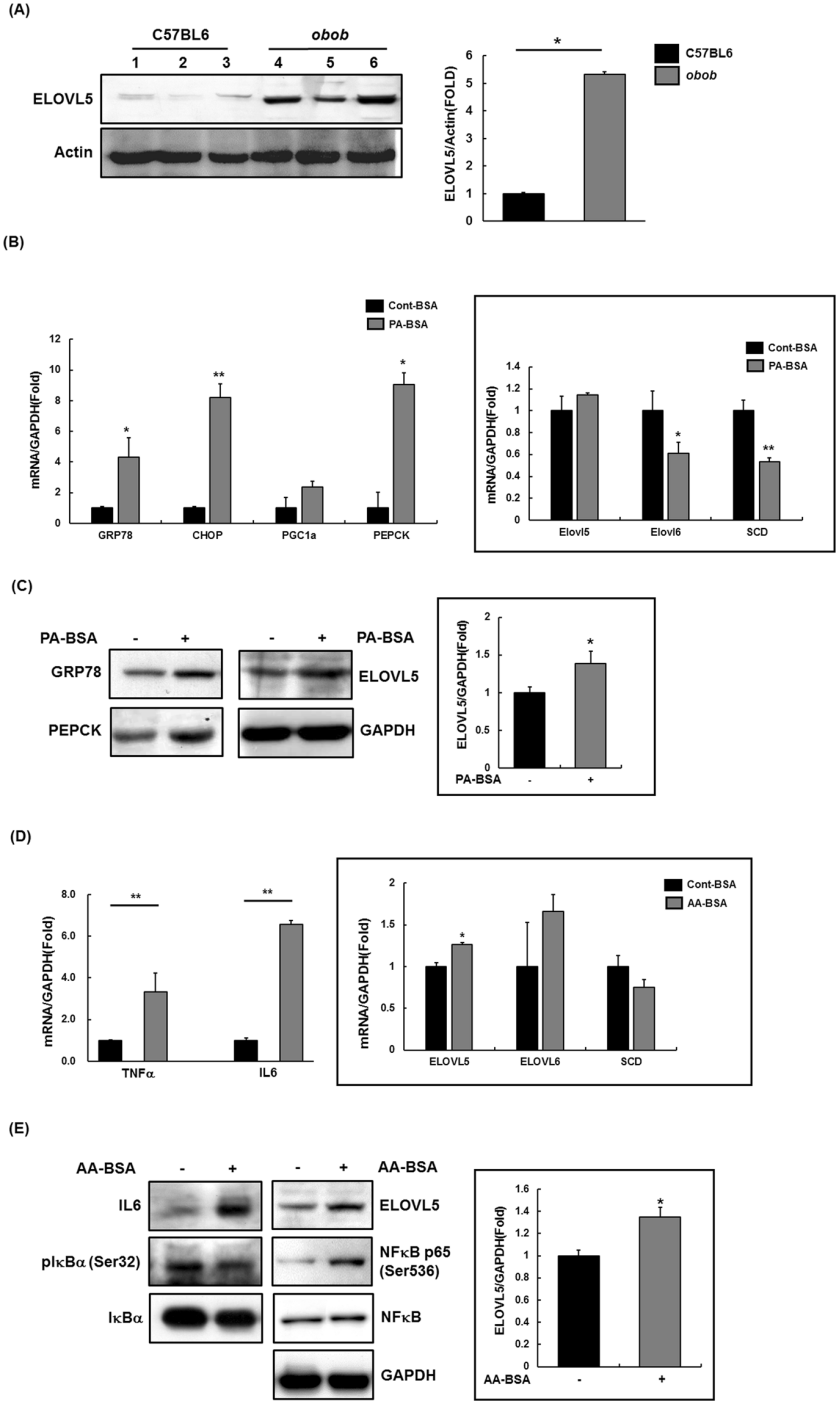
Expression of ELOVL5 on insulin resistance and inflammation causing T2D. (**A**) ELOVL5 expression identified in liver of *obob* mice using western blot. (**B**) Quantitative real time PCR was performed in SK-Hep I treated palmitate (0.5 mM) for 24 hr. (**C**) Whole lysate was extracted from palmitate-treated Sk-Hep I and western blot performed. (**D**) Quantitative real time PCR was performed in SK-Hep I treated arachidonic acid (0.05 mM) for 8 hr. (**E**) Whole lysate was extracted from arachidonic acid treated Sk-Hep I and western blot performed. All experimental results are presented as mean ± SE; n = 3; *p < 0.05; **p < 0.01.

To expand on the functional implications of *ELOVL5*, we studied its biological interactions using disease-specific cellular and mouse models. We first determined the protein expression levels in liver tissues of *ob/ob* mice and age-matched lean mice (C57BL/6J). Hepatic ELOVL5 expression was significantly augmented in *ob/ob* mice compared with lean mice (Figs 2A and S10A). We also measured ELOVL5 mRNA and protein levels in SK-Hep I cells treated with palmitate to induce insulin resistance. As predicted, mRNA and protein levels of gluconeogenesis-related PEPCK and endoplasmic reticulum stress-related factors GRP78 and CHOP were increased in the palmitate-treated SK-Hep I cell line. ELOVL5 expression was significantly upregulated, whereas mRNA levels were slightly increased and mRNA levels of other elongases (ELOVL6, SCD2) were decreased (Figs 2B,C and S10C).

Further, the expression of inflammation-related factors (TNFα, IL-6, plκBα, and pNFκB p65) was altered in cells treated with arachidonic acid (AA) to induce inflammatory reactions. Under these conditions, we also confirmed that mRNA and protein levels of ELOVL5 were significantly upregulated (Figs 2D,E and S10E). These findings demonstrated that ELOVL5 was associated with insulin resistance and inflammation, which cause T2D.

## Discussion

T2D is a complex metabolic disorder with underlying obesogenic environmental factors^20^. During the past decade, genome-wide studies have identified ~75 genetic loci for T2D. Integrative genetic-epigenetic studies have demonstrated that some of the known T2D loci are associated with epigenetic regulation in human peripheral blood^21–24^. Epigenome-wide approaches by Ling *et al*. identified altered DNA methylation^15,25–27^ and blood-derived biomarkers associated with insulin secretion and T2D in human pancreatic islets^18^. However, the precise molecular mechanisms remain unknown.

Adipose tissue functions as a metabolic and endocrine organ for obesity-mediated T2D development. Epigenetic factors such as DNA methylation play an important role in regulating gene expression. Given the role of adipose tissue in the development of T2D, we studied epigenome-wide DNA methylation differences (especially “genetically independent” DMR) within genetically identical twins (stage I) and identified T2D-associated giDMR (cg18681426) in the *ELOVL5* gene across ethnic groups (stage II) (Table 1). Further, we investigated the concordant (corresponding) epigenetic effect between blood and adipose tissue DNA methylation in human preadipocyte and adipocyte derived from the same adipose tissue sample (stage III, Fig. S2). Therefore, we found T2D-associated blood DNA methylation as a surrogate in human adipose tissue as performing the same statistical analysis (one-sample parametric t-test) in the analytical phase. Also, we studied biological correlation between DNA methylation and expression by profiling differentially expressed genes between preadipocytes and adipocytes from unrelated subjects with T2D (stage IV, Table 2). To evaluate endogenous data quality, we additionally confirmed adipose tissue specific expression marks that are previously known to be overexpressed in preadipocytes and adipocytes^19^ (Tables S4 and 5, Fig. S5). Finally, we studied the functional implications of ELOVL5 using the liver tissue of well-established mouse model and cell lines (stage VI, Fig. 2).

Genome-wide methylomic analyses are particularly enhanced by the study of trait-discordant MZTW, who share a complete genomic sequence^8^. These approaches allow us to detect moderate epigenetic effects by controlling for diverse confounding factors in T2D risk (Fig. S7). Recently, an epigenomic analysis identified genetically independent differentially methylated signals specific to T2D-discordant twins using a one-sample parametric *t*-test^13^. In this multi-stage association study, we focused on blood DNA methylation and its biological relevance in human adipose and pancreatic tissues. Despite insufficient availability of MZ twin cohorts to detect unbiased biological epi-variants, our meta analysis demonstrated straightforward enrichment of associations across the two studies beyond chance expectation. Also, there was significant directional consistency of the association between DNA methylation and gene expression between blood and human adipose tissue levels. Further replication studies using an enhancer promoter informed content array will be required to detect regulatory epi-variants.

ELOVL5 is involved in the elongation of long-chain polyunsaturated fatty acids and is highly expressed in human adipose tissue (subcutaneous and visceral) at the mRNA and protein levels (Fig. S8). ELOVL5 belongs to the ELO family (ELOVL1−7 in mammals). Of this family, ELOVL2, ELOVL5, and ELOVL6 were significantly associated with protein domain (GNS1/SUR4 family, *P* = 7.34e−08), KEGG pathway (polyunsaturated fatty acid biosynthesis, *P* = 4.67e−06), and molecular function (gene ontology) (transferase activity, *P* = 1.37e−04) in a network-based enrichment analysis (Fig. S9). These findings imply functional connectivity in a shared pathogenesis.

Jump *et al*. studied the functional effects of Elovl5 activity in controlling hepatic triglyceride catabolism and carbohydrate composition in high-fat diet-induced obese mice^28–30^. In human genomic studies, there were no obvious genetic associations between ELOVL5 and T2D risk. However, genome-wide studies identified single nucleotide polymorphisms (SNPs) in the ELOVL5 gene that contributed to the development of primary open-angle glaucoma (POAG) including late-onset normal tension glaucoma in Japanese populations^31,32^. Diabetes is considered a risk factor for POAG, as they share a common pathogenesis^33^. In ENCODE annotation analyses, we observed that an intronic variant, rs209485, 53 bp proximal to CpG site cg18681426, was associated with epigenetic modifications in six binding proteins (EBF1, HDAC2, POL24H8, TAL1, GATA1, and POL2) and an enhancer histone mark (H3K4me1) in adipose-derived mesenchymal stem cells (data not shown).

Interestingly, Ling *et al*. observed that the ELOVL6 gene influenced metabolism and inflammation using differential mRNA expression data from T2D-discordant MZTW adipose tissues. They suggested that reduced ELOVL6 gene expression, indicating oxidative phosphorylation, is significantly associated with decreased mitochondrial DNA content in adipose tissue from subjects with T2D^27^. In addition, a genome-wide study demonstrated that ELOVL2 is strongly associated with serum metabolite concentrations in metabolism-related genes^34^. Similarly, our studies in cellular and mouse models showed that *ELOVL5* levels were significantly upregulated in *ob/ob* mice and in a human liver cell line inducing insulin resistance and inflammation. These results indicate that ELOVL5 expression is associated with T2D.

In this study, we identified the *ELOVL5* gene as a new epigenetic mark in an epigenome-wide analysis of the blood DNA methylome using T2D-discordant MZTW models. We suggest that blood-derived epigenetic alterations in *ELOVL5* reflect both DNA methylation and RNA expression changes in human adipose and pancreatic islet tissues. These findings may provide new insights into epigenetic architecture by uncovering methylation-based biomarkers in common complex diseases.

## Materials and Methods

### Study participants

Eleven pairs of T2D-discordant MZTW were selected from the TwinsUK cohort. Participant information was collected by interviews and questionnaires. Based on WHO diagnosis guidelines, T2D subjects were selected based on the following criteria: (1) past medical and family history of T2D, (2) fasting plasma glucose ≥7 mmol/L or plasma glucose 2 h after ingestion of 75 g oral glucose ≥11.1 mmol/L, and (3) age of disease onset ≥40 years. The inclusion criteria of normal controls were as follows: (1) no past medical or family history of T2D, and (2) fasting plasma glucose <5.6 mmol/L and plasma glucose 2 h after ingestion of 75 g oral glucose <7.8 mmol/L. Procedures were in accordance with institutional guidelines and approved by an institutional review committee. Written informed consent was obtained from all study participants. The study protocol was approved by the institutional review board of the Korea Center for Disease Control and Prevention (2017-02-06-P-A).

### DNA isolation and bisulphite conversion

Whole blood was collected from participants at the time of the interview from the TwinsUK cohort. Genomic DNA was then isolated with a DNA purification kit (Norgen Biotek Corporation, Thorold, Canada). Single beta cells were isolated from islet tissues from pancreatic cancer patients diagnosed at Asan Medical Center. To detect positive selection of beta cells, we used polysialic acid-neural cell adhesion molecule (PSA-NCAM) as a beta-cell-specific surface antigen^35^. Pancreatic beta cell purity (>95%) was confirmed by staining with dithizone (DTZ). DNA samples extracted from blood and tissues were quantified with picogreen (Invitrogen, Carlsbad, CA, USA) and a Victor 3 spectrophotometer (PerkinElmer, Waltham, MA, USA). DNA quantity was measured with NanoDrop ND-1000 spectrophotometer (NanoDrop Technologies, Wilmington, DE, USA). After bisulfite conversion of the DNA samples (500 ng) with the EZ-96 DNA Methylation kit (Zymo Research, Irving, CA, USA), the cytosines in the CpG sites were genotyped according to the manufacturer’s protocol.

### Genome-wide DNA methylation

Genome-wide DNA methylation was assessed using the Infinium HumanMethylation450 BeadChip (Illumina Inc., San Diego, CA, USA). BeadChips were imaged with an Illumina iScan and then called using the GenomeStudio software (v2010). Further analyses were performed using the R package RnBeads from Bioconductor^36^. We used a score called a “β value” for each CpG site, which represents the ratio of the intensity of the methylated bead signals over the sum of the methylated and unmethylated bead signals. All CpG sites with a detection threshold *P*-value < 0.05 were considered for subsequent analysis. The methylation data were background corrected by subtracting the median intensities of internal control probes and then normalized using beta-mixture quantile normalization (BMIQ) in data preprocessing^37^.

### T2D-giDMR replication

A total of 17 T2D-discordant MZTW pairs from the TwinsUK registry were used for replication^13^. Briefly, all participants provided written informed consent in accordance with the St. Thomas Hospital local ethics research committee. T2D cases were determined by fasting glucose ≥7 mmol/L and/or self-reported via questionnaire. DNA methylation was measured using methylated DNA immunoprecipitation and high-throughput sequencing (MeDIP-seq), as described previously^13^. Reads were mapped with Novoalign V2.07.11 to the human genome version 19 (hg19) and quantified with MEDIPS in regions of 500 bp. Regions harboring CpG sites identified in the discovery phase were analyzed for replication. Within-discordant twin pair differences were tested using a one-sample parametric *t*-test.

### mRNA sequencing

The quantity and quality scores of RNA samples were based on fluorometric (>0.5 μg) and RIN values (>7). The poly(A)-based TruSeq V2 RNA sample preparation kit was used according to the manufacturer’s instructions. Image analysis and base calling were performed using Illumina pipeline version 1.5.15.1. cDNA libraries (fragment size: 200–500 bp) were loaded into the Illumina HiSeq 2000. Reads were aligned with TopHat 2.0.6 using the GRCh 37 (hg19) reference. All gene FPKMs were calculated by Cufflinks.

### Statistical analyses

Differentially methylated regions (DMRs) and giDMRs were analyzed using SAS software (version 9.1; SAS Institute, Inc., Cary, NC, USA). In a linear mixed effects model, age, sex, and BMI were incorporated as fixed effects and family structure was also included as a random effect. giDMRs were characterized by phenotypic consequences underlying pure environmental status. *P*-values were calculated from one-sample parametric *t*-tests^13^. To perform additional association tests, visual inspection was analyzed with the R v2.15.1 software package.

### Functional annotation analysis

Functional annotation and visualization for gene ontology, biological pathways, and regulatory enrichment were tested using the WebGestalt program (http://bioinfo.vanderbilt.edu/webgestalt/), the HaploReg program (http://www.broadinstitute.org/mammals/haploreg/haploreg.php), the Methylation Plotter (http://gattaca.imppc.org:3838/methylation_plotter/) and the Roadmap Epigenome Browser (http://epigenomegateway.wustl.edu/browser/roadmap/). The gene expression data set was retrieved from the NCBI Gene Expression Omnibus (GEO). We performed GEO2R comparisons (“a simple interface that allows users to perform R statistical analysis”) on original submitter-supplied processed data (GSE38642) using the GEOquery and limma R packages from the Bioconductor. The GEOquery R package parses GEO data into R data structures. The limma (Linear Models for Microarray Analysis) R package is one of the most widely used statistical tests for identifying differentially expressed genes (DEGs). Functional connectivity and networks were analyzed using the STRING database (http://string-db.org/) and the ingenuity pathway analysis (IPA) (https://analysis.ingenuity.com/).

### Chemicals and animals

Palmitate and arachidonic acid were purchased from Sigma-Aldrich (St. Louis, MO, USA) and Nu-chek Prep, Inc. (Elysian, MN, USA), respectively. Palmitate- and arachidonic acid-bovine serum albumin (BSA) solutions were prepared by dissolving palmitate in ethanol and then mixing it with fatty acid-free BSA (2% wt/vol in water; Sigma-Aldrich) at 37 °C on a shaker for 2 h. We obtained 7-week-old male *ob/ob* mice and age-matched lean mice (C57BL/6J) from the Animal Center of SLC, Inc. (Hamamatsu, Shizuoka, Japan). The mice were housed in individual cages at 22 ± 2 °C with a 12-h light-dark cycle. After overnight fasting, the liver was removed from each mouse and used for western blot analysis. All animal experiments were approved by the institutional animal care and use committee of the Korea Center for Disease Control and Prevention (KCDC-015-11-2A). The methods were carried out in accordance with the approved guidelines.

### Cell culture

SK-Hep I human liver cells (ATCC CRL 1772; American Type Culture Collection, Manassas, VA, USA) were cultured using Dulbecco’s modified Eagle’s medium (DMEM) supplemented with 10% fetal bovine serum (FBS) and antibiotics. However, we cultured these cells using DMEM supplemented with 2% FBS and antibiotics when they were treated with palmitate-BSA solution (500 µM) for 24 h or arachidonic acid-BSA solution (50 uM) for 8 hr.

### Western blotting

At the end of each treatment regimen, whole cell lysate was prepared by incubating cells on ice with lysis buffer (50 mM Tris-Cl (pH 7.5), 20 mM NaCl, 5 mM EDTA, 1% TX-100, 0.1% sodium dodecyl sulfate (SDS), 5% glycerol, and protease inhibitor), followed by ultrasonication for 10 s (Sonics & Materials Inc., Newtown, CT, USA). After centrifugation at 12,000 rpm for 20 min, the supernatants were subjected to SDS-polyacrylamide gel electrophoresis (PAGE) and then transferred to a polyvinylidene fluoride (PVDF) membrane. After transfer, the membrane was blocked and then probed with antibodies. Immunoblots were visualized using an ECL chemiluminescence detection kit (Thermo Fisher Scientific, Waltham, MA, USA). ELOVL5, PEPCK, and GRP78 antibodies were purchased from Santa Cruz Biotechnology (Santa Cruz, CA, USA). All other antibodies were purchased from Cell Signaling Technology (Beverly, MA, USA).

### Quantitative reverse transcription PCR (qPCR)

Total RNA was isolated from tissues and Sk-Hep I cells using the RNeasy Mini Kit (QIAGEN, Hilden, Germany). cDNA synthesis was performed with 2 μg of total RNA in 20 μL using oligo(dt) primers and Superscript III reverse transcriptase (Invitrogen). QPCR analyses of the genes described in Supplementary Table X were performed using the QuantStudio 6 Flex Real-Time PCR System (Applied Biosystems, Foster City, CA, USA). Reactions were performed in a 20-μL volume containing 10 μL 5X SYBR Green PCR master mix (Applied Biosystems), 1 μL cDNA, and 5 pmol of each primer. After an initial incubation for 2 min at 50 °C, the cDNA was denatured at 95 °C for 10 min followed by 40 cycles of PCR (95 °C for 15 s, 60 °C for 60 s). Data analyses were performed on QuantStudio^TM^ Real-Time PCR software v1.1 (Applied Biosystems). All samples were normalized to the corresponding expression levels of glyceraldehyde-3-phosphate dehydrogenase (GAPDH).

## Electronic supplementary material

Supplementary Information
