# Supplementary material for: Genome-wide methylation analysis identifies ELOVL5 as an epigenetic biomarker for the risk of type 2 diabetes mellitus
Source: Sci Rep. 2018 Oct 5;8:14862. doi: 10.1038/s41598-018-33238-9 (PMC6173741; doi:10.1038/s41598-018-33238-9)
Supplement: Supplementary file 1 — Supplementary Information [file 41598_2018_33238_MOESM1_ESM.pdf]

Genome-wide methylation analysis identifies ELOVL5 as an epigenetic biomarker for the risk of type 2 diabetes mellitus

Joo-Yeon Hwang[a,b], Hyo Jung Lee[b], Min Jin Go[a], Han Byul Jang[b], Nak-Hyun Choi[a], Jae Bum Bae[a], Juan E. Castillo-Fernandez[c], Jordana T. Bell[c], Tim D. Spector[c], Hye-Ja Lee[b,1] and Bong-Jo Kim[a,1]

[a]Center for Genome Science, National Institute of Health, Osong Health Technology Administration Complex, Chungcheongbuk-do, Republic of Korea

[b]Center for Biomedical Science, National Institute of Health, Osong Health Technology Administration Complex, Chungcheonbuk-do, Republic of korea

[c]Department of Twin Research & Genetic Epidemiology, King's College London, London SE1 7EH, UK

[1]Corresponding author

Hye-Ja Lee, Phone: +82-43-719-8692, Fax: +82-43-719-8602, Email: hyejalee@yahoo.co.kr

Bong-Jo Kim, Phone: +82-43-719-8870, Fax: +82-43-719-7219, Email: kbj6181@cdc.go.kr

File name: Supplementary Information

Description: Supplementary Figures and Supplementary Tables

File name: Supplementary Data 1

Description: Gene set list for pathway enrichment analysis for giDMR genes in T2D-discordant MZ twin pairs.

File name: Supplementary Data 2

Description: Gene set enrichment analysis for giDMR genes in T2D-discordant MZ twin pairs.

File name: Supplementary Data 3

Description: Gene set list for pathway enrichment analysis for giDMR genes in in human preadipocytes and adipocytes.

File name: Supplementary Data 4

Description: Gene set enrichment analysis for giDMR genes in human preadipocytes and adipocytes.

Supplementary Information

Supplementary Table 1. Descriptive characteristics and statistics in T2D-discordant MZ twin pairs.

Variable	T2D	Control
Sex (M/F)	5 / 6	5 / 6
Age (yr)	42.18 ± 8.35	42.18 ± 8.35
Body mass index (kg/m2)	24.08 ± 2.56	24.43 ± 2.27
Low-density lipoprotein Cholesterol (mg/dl)	123 ± 30.95	107 ± 29.42
High-density lipoprotein Cholesterol (mg/dl)	42.91 ± 8.99	53.82 ± 23.88
Fasting plasma glucose (mmol/L)	8.08 ± 2.6	6.49 ± 1.42

Supplementary Table 2. Sample characteristics for giDMRs in the non-diabetic human adipose tissue donors (n=8).

Variable	Mean ± SD
Sex	Female
Age (yr)	60.50 ± 5.07
Height (cm)	149.45 ± 2.28
Weight (kg)	65.94 ± 12.11
BMI (kg/m2)	29.48 ± 5.02
SBP (mmHg)	116.75 ± 15.73
DBP (mmHg)	85.5 ± 14.73

Supplementary Table 3. Sample characteristics for DEGs in preadipocytes and adipocytes.

Variable	adipocyte	pre-adipocyte
Sex (M/F)	1 / 6	0 / 6
Age (yr)	63.71 ± 6.26	66.83 ± 3.31
Height (cm)	163.59 ± 6.61	167.67 ± 3.47
Weight (kg)	72.24 ± 13.30	76.82 ± 20.80
BMI (kg/m2)	26.81 ± 3.43	27.42 ± 7.61
SBP (mmHg)	118.43 ± 14.88	124.67 ± 7.84
DBP (mmHg)	75.86 ± 7.58	79.33 ± 9.22
FPG (mmol/L)	9.11 ± 3.57	8.96 ± 3.43
GLU120 (mmol/L)	13.90 ± 9.02	13.15 ± 8.60
HbA1c (%)	7.47 ± 2.16	7.02 ± 0.84

Supplementary Table 4. Summary of previously known DEGs for preadipocyte-specific expression.

Gene	Description	*P*-value[a]	*P*-value[b]
Transcription factors			
E2F4	E2F transcription factor 4	0.0204	0.041602*
PPARD	PPARδ	0.0428	0.046304*
Hormone, receptors and signaling molecules			
PTPRN	Protein tyrosine phosphatease receptor N	0.0466	0.034793*
Cellular matrix and cytoskeleton			
LOXL1	Lysl oxidase L1	0.0002	0.192687
LOX	Lysyl oxidase	0.0017	0.005391*
COL3A1	Collagen type 3 A1	0.0043	0.135406
COL5A1	Collagen type 5 A1	0.0002	0.52462
COL6A1	Collagen type 6 A1	0.0013	0.098899
COL6A3	Collagen type 6 A3	0.0049	0.07238
THBS2	Thrombospondin 2	0.0003	0.286955
MMP2	Matrix metalloprotein 2	0.0005	0.185501
DCN	Decorin	0.0008	0.400615
LUM	Lumican	0.0001	0.049838*
PPP2R1A	Protein phosphatase 2 (formerly 2A), regulatory subunit 1A (PR 65)	0.0001	0.466572

[a] *P*-values of previously known DEGs for preadipocyte were obtained from data by Urs S, et al.

[b] *P*-values in this study.

Supplementary Table 5. Summary of previously known DEGs for adipocyte-specific expression.

Gene	Description	*P*-value	*P*-value
Metabolism (primarily lipid and carbohydrate) and transport			
LPL	Lipoprotein lipase	0.0001	0.043873*
FABP4	Fatty acid binding protein 4	0.0002	0.029077*
FABP5	Fatty acid binding protein 5	0.0005	0.086621
GPD1	Glycerol 3 phosphate dehydrogenase 1	0.0001	0.392569
PLIN1	Perilipin 1	0.0001	0.06504
FXYD1	FXYD domain containing ion transport regulator 1	0.0004	0.2338
CTSG	Cathepsin G	0.0008	0.133232
ALDH6A1	Aldehyde dehydrogenase 6 family member A1	0.0008	0.064601
LIPE	Lipase E, hormone sensitive type	0.0002	0.561167
DGAT1	Diacylglycerol *O*-acyltransferase 1	0.0025	0.140209
GPX3	Glutathione peroxidase 3	0.0055	0.02157*
PLEK	Pleckstrin	0.0079	0.02745*
AGT	Angiotensinogen	0.0343	0.092
CRYAB	Crystalline, αB	0.0372	0.02186*
SPTBN4	Spectrin beta, non-erythrocytic 4	0.001	0.03693*
MGST1	Microsomal glutathione S-transferase 1	0.0015	0.05687
PFKFB3	6-Phosphofructo-2-kinase/fructose-2,6-biphosphatase 3	0.007	0.345025
GLUL	glutamate-ammonia ligase	0.0429	0.00302*
Transcription factors and binding proteins			
PPARG	PPARγ	0.0008	0.124407
STAT5B	Signal transducer and activator of transcription 5B	0.0001	0.14924
RXRA	Retinoid X receptor A	0.0002	0.076394
CEBPD	C/EBPδ	0.0044	0.455117
Signaling molecules			
INSR	Insulin like receptor	0.0001	0.381202
CAP2	Adenylate cyclase associated protein 2	0.001	0.14862
PTPRS	Protein tyrosine phosphatase receptor S	0.0225	0.224986
Cellular matrix and cytoskeleton			
ECM2	Extracellular matrix protein 2	0.002	0.522223
DPT	Dermatopontin	0.0169	0.010096*

Supplementary Table 6. Sample characteristics for DEGs in human pancreatic islet donors.

Variable	T2D pancreatic donors	non-diabetic donors
Sex (M/F)	2 / 3	2 / 3
Age (yr)	47.2	47.6
BMI (kg/m2)	28.26 ± 4.46	23.76 ± 1.04
HbA1c (%)	7.06 ± 0.42	5.32 ± 0.19

Supplementary Figure 1. Manhattan plot for giDMRs in T2D-discordant MZ twins.

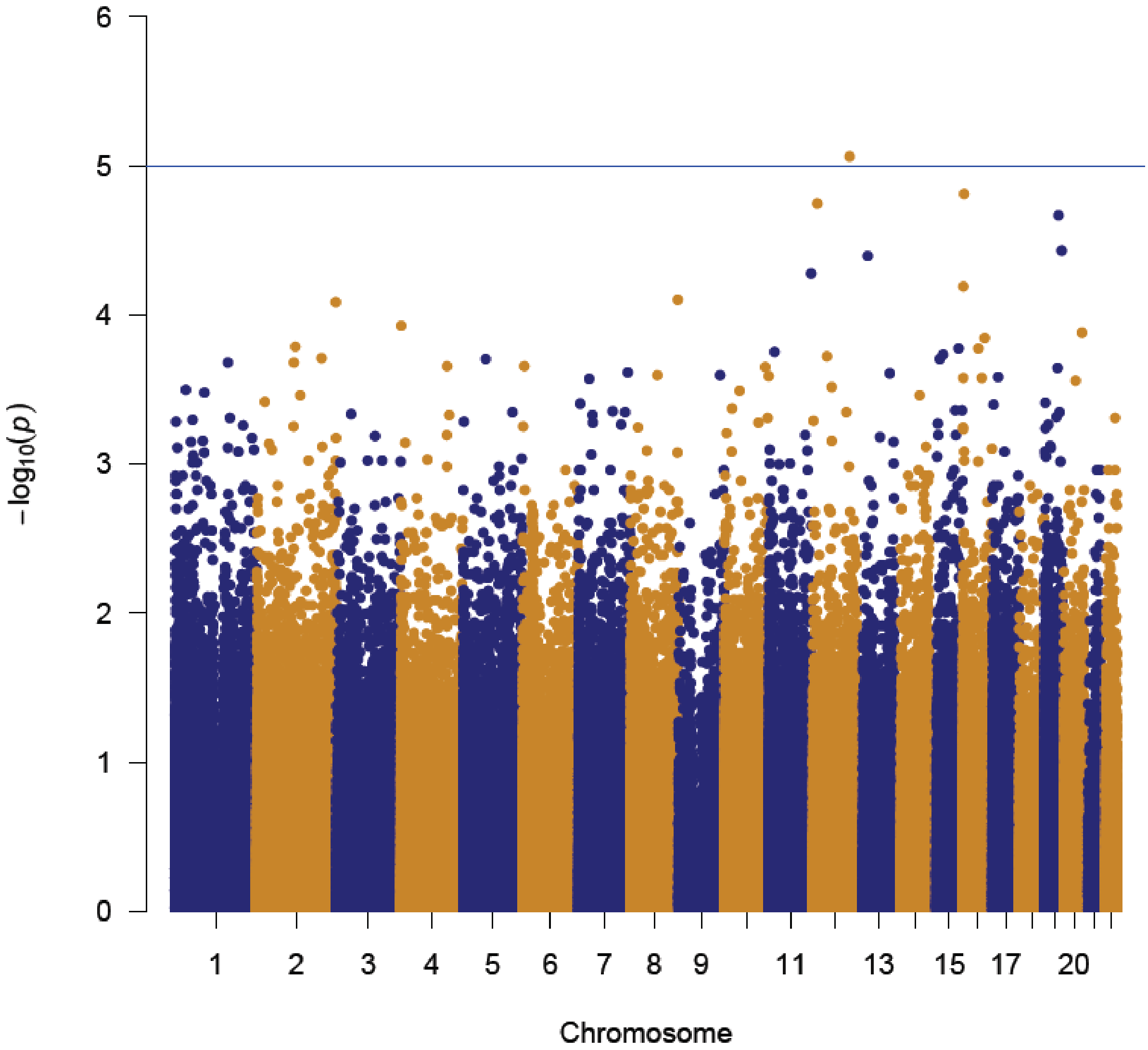

Supplementary Figure 2. PCA plot for methylome in preadipocytes and adipocytes isolated from human adipose tissues.

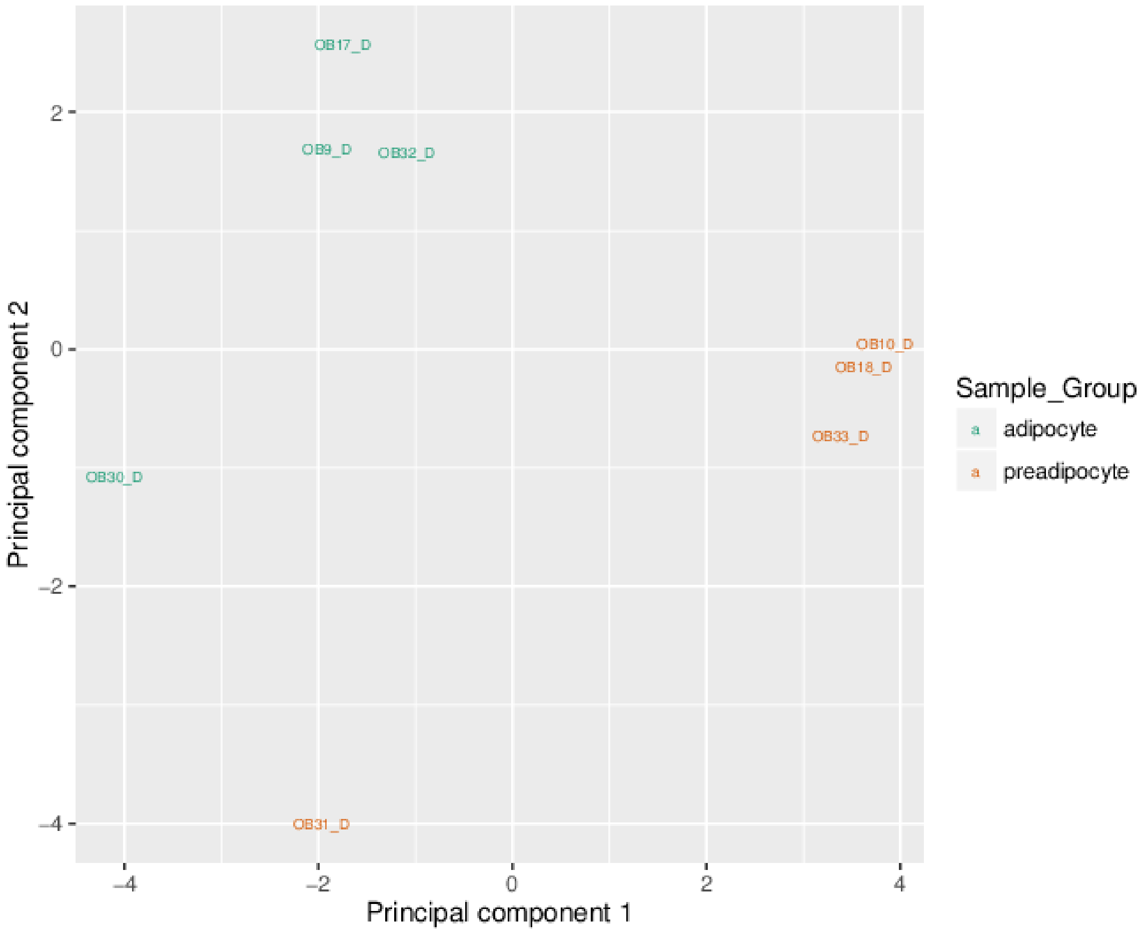

Supplementary Figure 3. Differential expression analysis in preadipocytes and adipocytes isolated from human adipose tissues. X-axis, sample; Y-axis, cluster; Bar, density

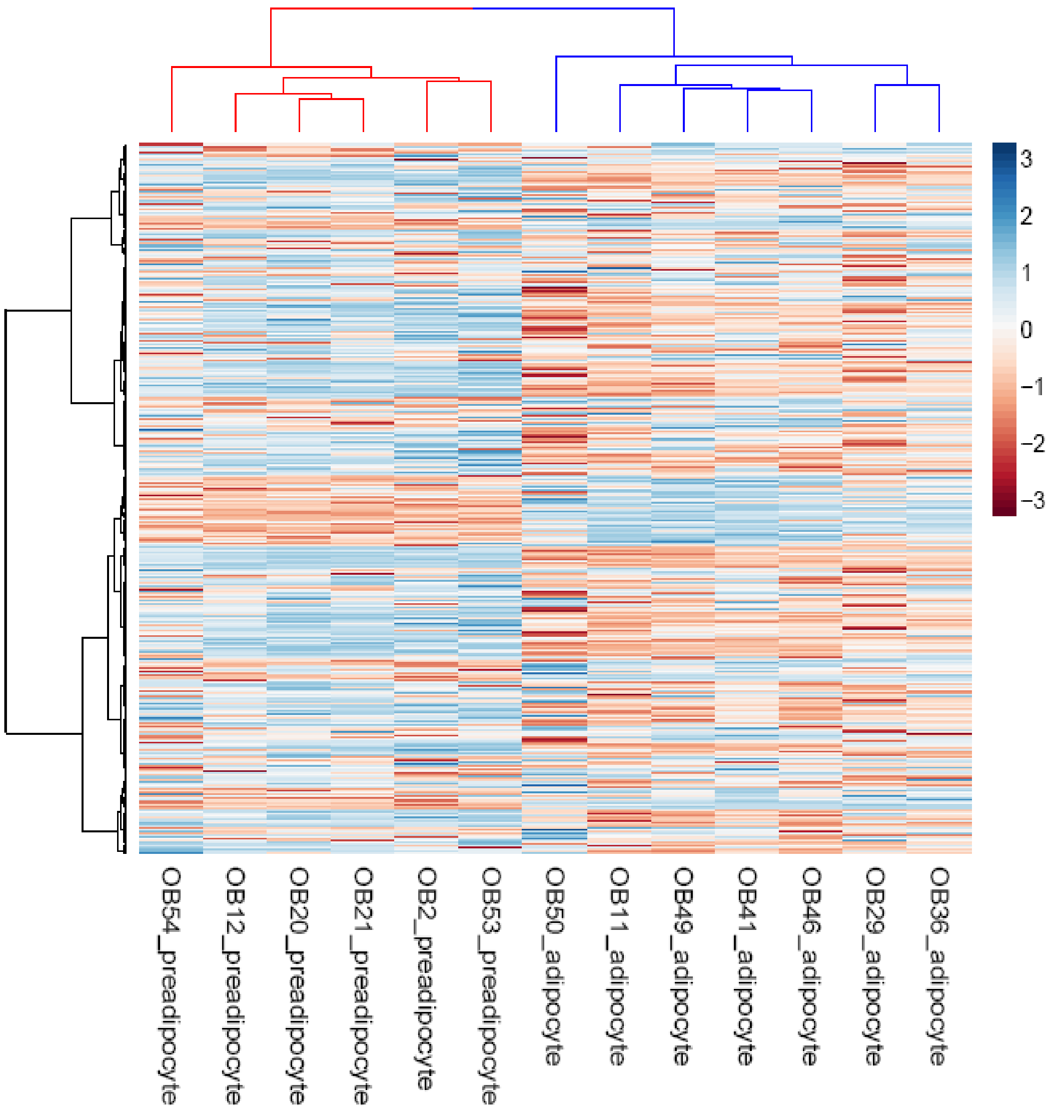

Supplementary Figure 4. Hierachical clustering dendrogram for transcriptome in preadipocytes and adipocytes. X-axis, sample; Y- axis, height

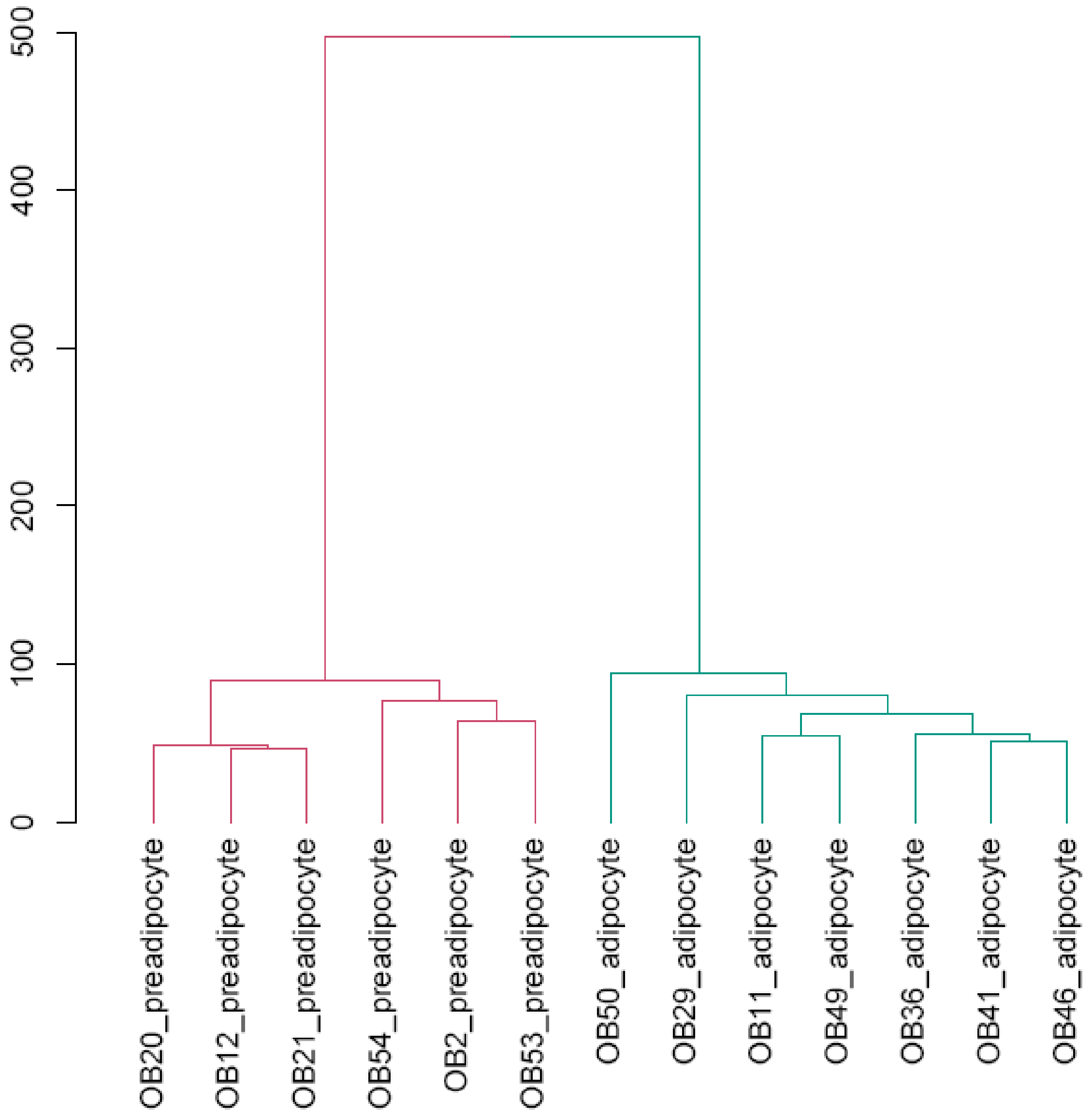

Supplementary Figure 5. Gene expression profiles for previous known genes specific to preadipocytes and adipocytes. X-axis, sample; Y- axis, target-gene expression value

(a) Gene overexpressed in human preadipocytes

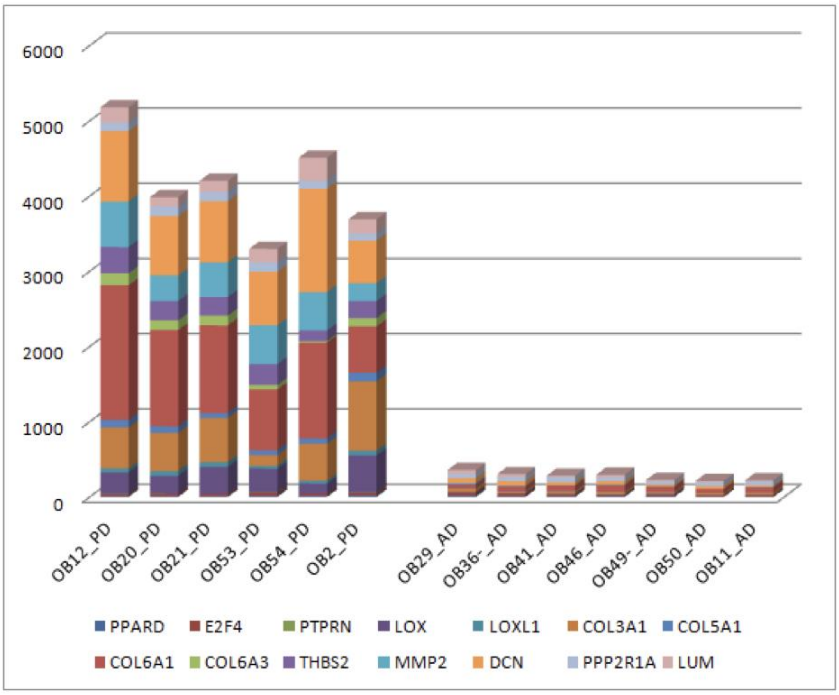

(b) Gene overexpressed in human adipocytes

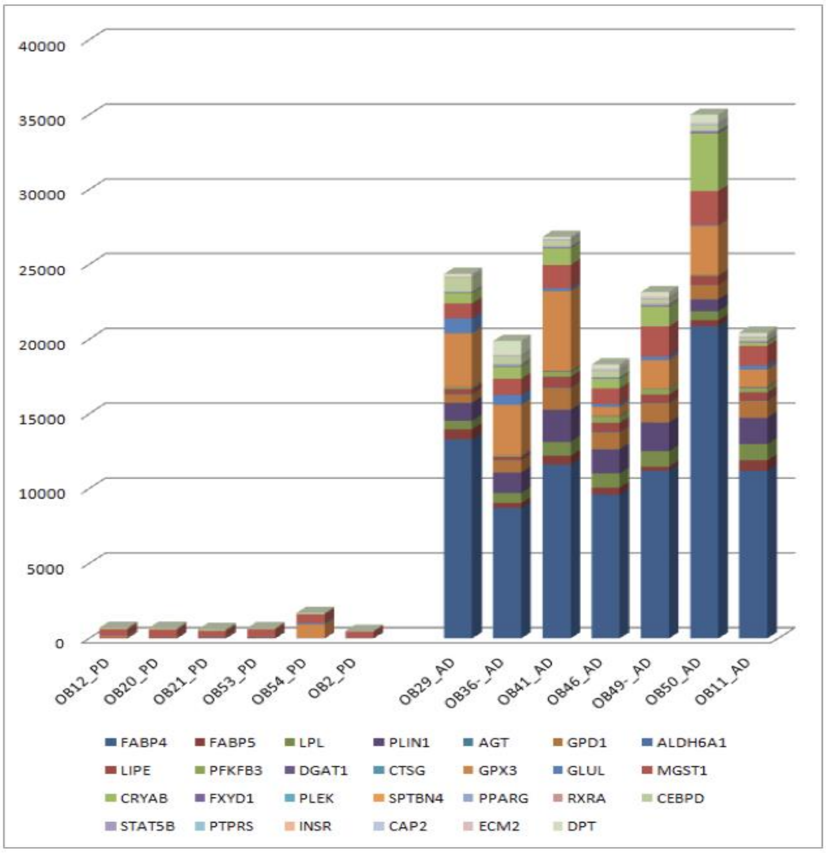

Supplementary Figure 6. GEO profiles in human pancreatic islets.

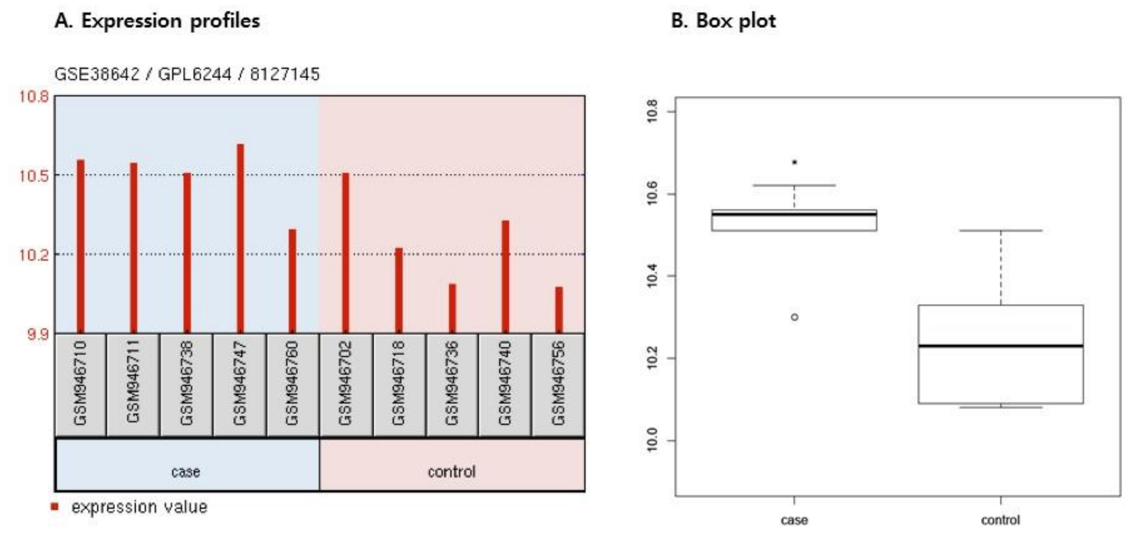

Supplementary Figure 7. Chromatin accessibility within DHSs of trait-determining cell types.

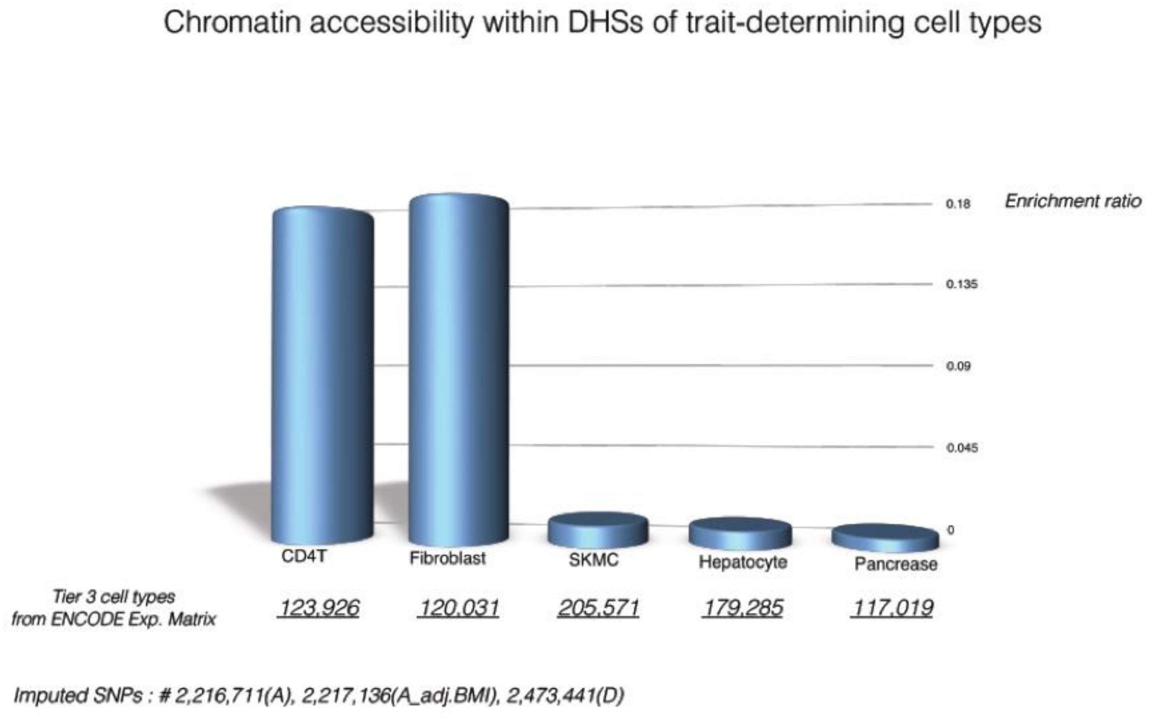

Supplementary Figure 8. Gene expression profiles for ELOVL5 gene from GTEx.

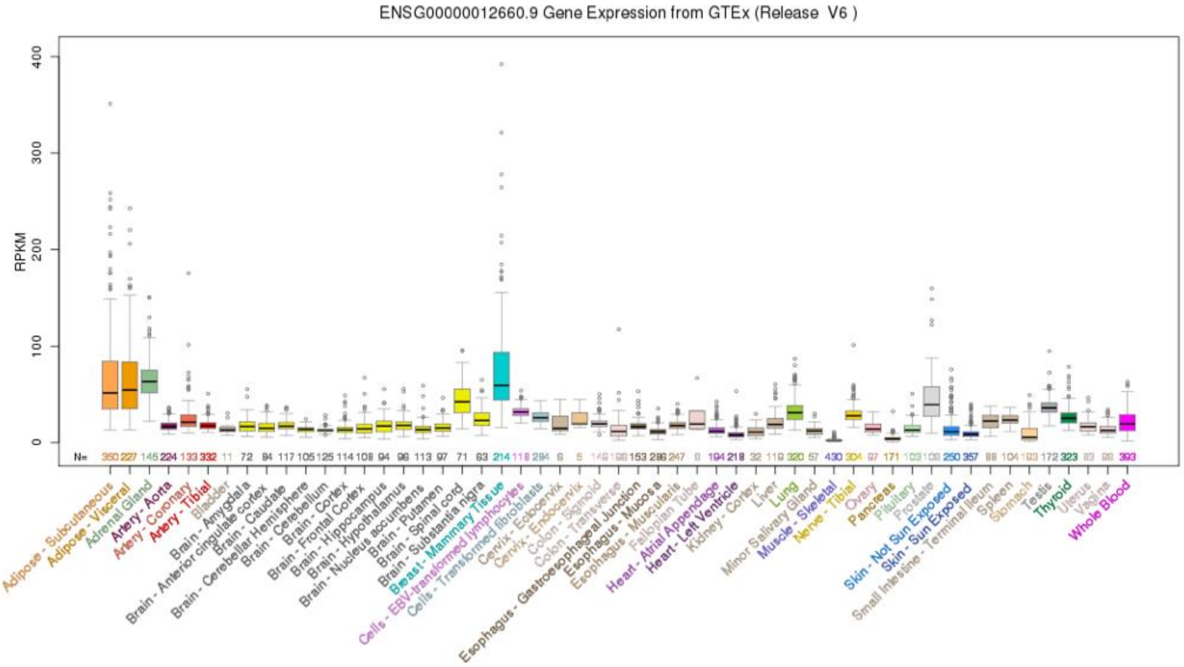

Supplementary Figure 9. Functional connectivity network based on the ELO family. Colored nodes, first shell of interactions; White nodes, second shell of interactions; Colored lines, protein homology, text mining, and coexpression

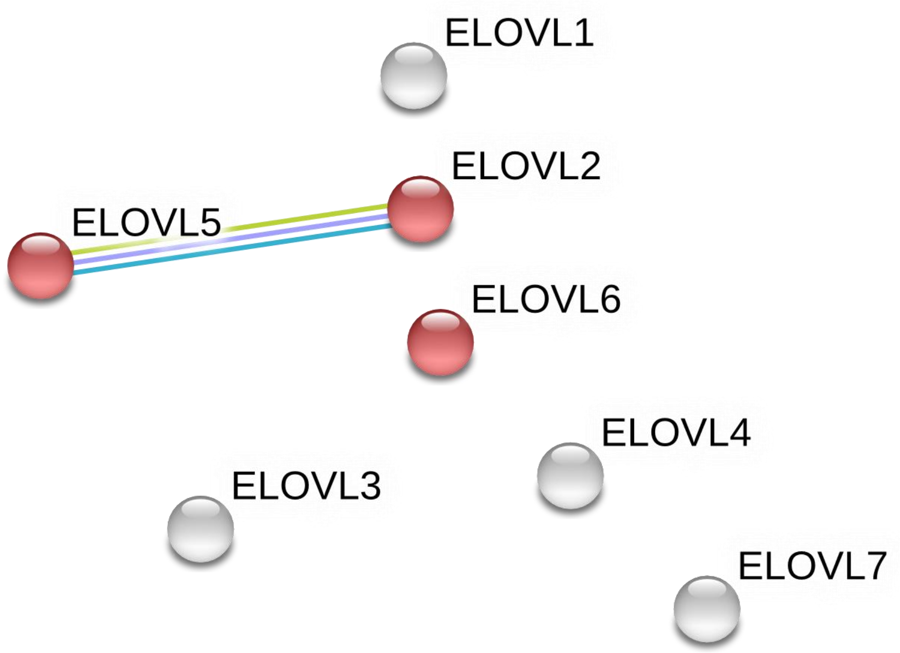

Supplementary Figure 10. Middle-length blots and two exposures of Figure 2.

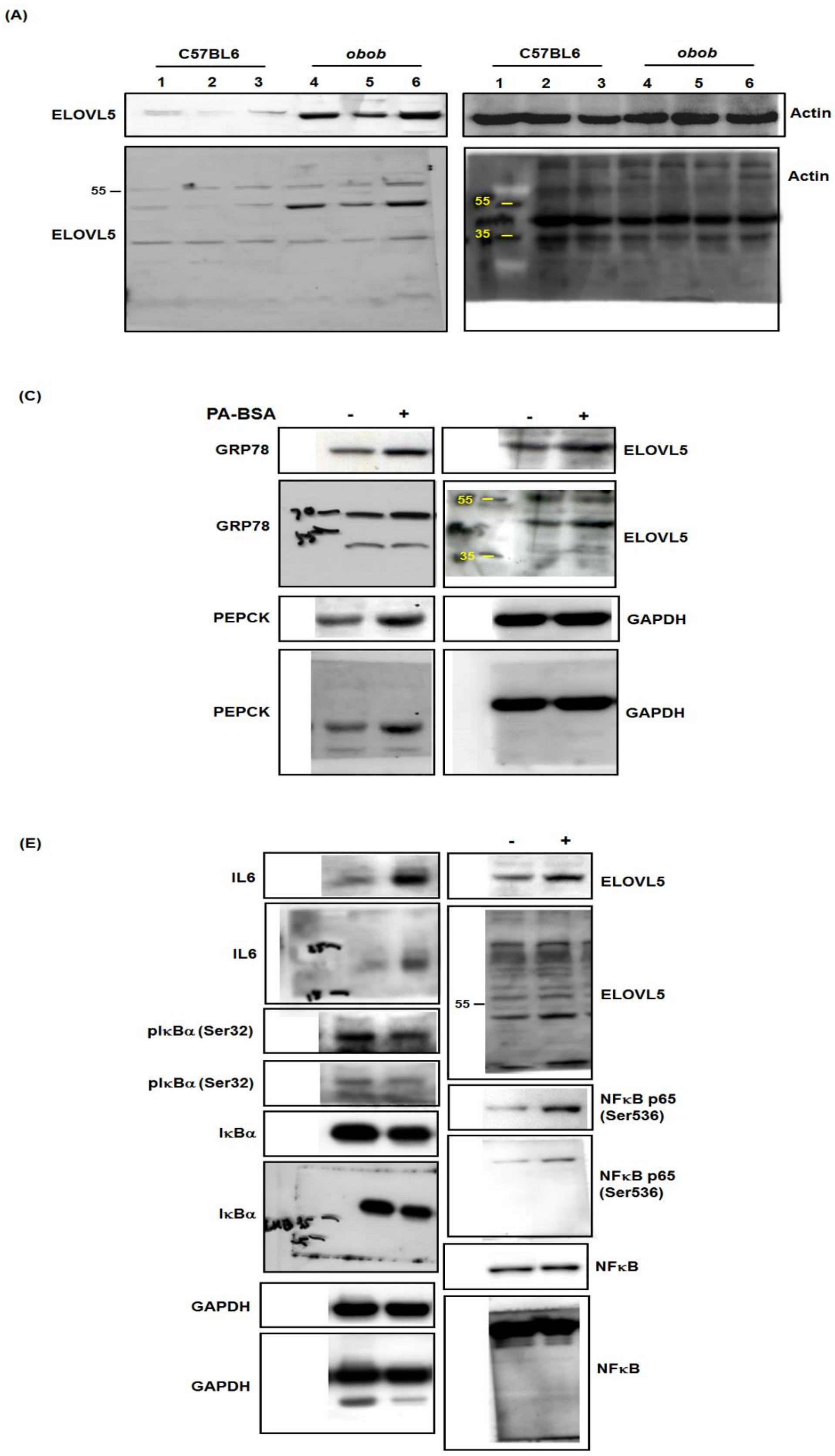

Supplementary Data 1. Gene set list for pathway enrichment analysis for giDMR genes in T2D-discordant MZ twin pairs

Gene Symbol	Gene Name	EntrezGene	Ensembl
PNCK	pregnancy up-regulated non-ubiquitously expressed CaM kinase	139728	ENSG00000130822
AACS	acetoacetyl-CoA synthetase	65985	ENSG00000081760
ELMO2	engulfment and cell motility 2	63916	ENSG00000062598
CHPT1	choline phosphotransferase 1	56994	ENSG00000111666
RPTOR	regulatory associated protein of MTOR, complex 1	57521	ENSG00000141564
HAUS2	HAUS augmin-like complex, subunit 2	55142	ENSG00000137814
FARP1	FERM, RhoGEF (ARHGEF) and pleckstrin domain protein 1 (chondrocyte-derived)	10160	ENSG00000152767
BARHL2	BarH-like homeobox 2	343472	ENSG00000143032
KCNMA1	potassium large conductance calcium-activated channel, subfamily M, alpha member 1	3778	ENSG00000156113
KIF26B	kinesin family member 26B	55083	ENSG00000162849
OR2T33	olfactory receptor, family 2, subfamily T, member 33	391195	ENSG00000177212
ATF6B	activating transcription factor 6 beta	1388	ENSG00000213676
LPP	LIM domain containing preferred translocation partner in lipoma	4026	ENSG00000145012
GOLT1A	golgi transport 1A	127845	ENSG00000174567
HECA	headcase homolog (Drosophila)	51696	ENSG00000112406
TACC2	transforming, acidic coiled-coil containing protein 2	10579	ENSG00000138162
DMRT1	doublesex and mab-3 related transcription factor 1	1761	ENSG00000137090
TNIK	TRAF2 and NCK interacting kinase	23043	ENSG00000154310
GLT1D1	glycosyltransferase 1 domain containing 1	144423	ENSG00000151948
PMPCA	peptidase (mitochondrial processing) alpha	23203	ENSG00000165688
MYO1B	myosin IB	4430	ENSG00000128641
PDCL3	phosducin-like 3	79031	ENSG00000115539
ZNF799	zinc finger protein 799	90576	ENSG00000196466
PANX1	pannexin 1	24145	ENSG00000110218
CUBN	cubilin (intrinsic factor-cobalamin receptor)	8029	ENSG00000107611
CXCL14	chemokine (C-X-C motif) ligand 14	9547	ENSG00000145824
SLC17A1	solute carrier family 17 (sodium phosphate), member 1	6568	ENSG00000124568
SEC24D	SEC24 family, member D (S. cerevisiae)	9871	ENSG00000150961
LDB3	LIM domain binding 3	11155	ENSG00000122367
MOB2	MOB kinase activator 2	81532	ENSG00000182208
ADCY7	adenylate cyclase 7	113	ENSG00000121281
HSPB8	heat shock 22kDa protein 8	26353	ENSG00000152137
DGAT2L6	diacylglycerol O-acyltransferase 2-like 6	347516	ENSG00000184210
PCMTD2	protein-L-isoaspartate (D-aspartate) O-methyltransferase domain containing 2	55251	ENSG00000203880
NTRK1	neurotrophic tyrosine kinase, receptor, type 1	4914	ENSG00000198400
MBNL2	muscleblind-like splicing regulator 2	10150	ENSG00000139793
FRAS1	Fraser syndrome 1	80144	ENSG00000138759
FRMD3	FERM domain containing 3	257019	ENSG00000172159
ATP13A1	ATPase type 13A1	57130	ENSG00000105726
TBC1D14	TBC1 domain family, member 14	57533	ENSG00000132405
GAL3ST3	galactose-3-O-sulfotransferase 3	89792	ENSG00000175229
TOP1MT	topoisomerase (DNA) I, mitochondrial	116447	ENSG00000184428
CNGB1	cyclic nucleotide gated channel beta 1	1258	ENSG00000070729
FAM134B	family with sequence similarity 134, member B	54463	ENSG00000154153
TRIO	trio Rho guanine nucleotide exchange factor	7204	ENSG00000038382
SCAND3	SCAN domain containing 3	114821	ENSG00000232040
ATXN7L1	ataxin 7-like 1	222255	ENSG00000146776
SIRPB2	signal-regulatory protein beta 2	284759	ENSG00000196209
RBFOX3	RNA binding protein, fox-1 homolog (C. elegans) 3	146713	ENSG00000167281
TICAM1	toll-like receptor adaptor molecule 1	148022	ENSG00000127666
KIF7	kinesin family member 7	374654	ENSG00000166813
CA13	carbonic anhydrase XIII	377677	ENSG00000185015
IGF2BP3	insulin-like growth factor 2 mRNA binding protein 3	10643	ENSG00000136231

DTNBP1	dystrobrevin binding protein 1	84062	ENSG00000047579
STK32C	serine/threonine kinase 32C	282974	ENSG00000165752
LOC100130331	POTE ankyrin domain family, member F pseudogene	100130331	NA
LGR6	leucine-rich repeat containing G protein-coupled receptor 6	59352	ENSG00000133067
CCNB1	cyclin B1	891	ENSG00000134057
UBB	ubiquitin B	7314	ENSG00000170315
TIA1	TIA1 cytotoxic granule-associated RNA binding protein	7072	ENSG00000116001
SP140L	SP140 nuclear body protein-like	93349	ENSG00000185404
ZSWIM1	zinc finger, SWIM-type containing 1	90204	ENSG00000168612
UPK1A	uroplakin 1A	11045	ENSG00000105668
AP1G1	adaptor-related protein complex 1, gamma 1 subunit	164	ENSG00000166747
CCDC12	coiled-coil domain containing 12	151903	ENSG00000160799
SYCP3	synaptonemal complex protein 3	50511	ENSG00000139351
GNAI3	guanine nucleotide binding protein (G protein), alpha inhibiting activity polypeptide 3	2773	ENSG00000065135
NETO2	neuropilin (NRP) and tolloid (TLL)-like 2	81831	ENSG00000171208
FHL3	four and a half LIM domains 3	2275	ENSG00000183386
SLC16A14	solute carrier family 16, member 14 (monocarboxylic acid transporter 14)	151473	ENSG00000163053
PDE1C	phosphodiesterase 1C, calmodulin-dependent 70kDa	5137	ENSG00000154678
FLJ12825	uncharacterized LOC440101	440101	NA
RAD51B	RAD51 homolog B (S. cerevisiae)	5890	ENSG00000182185
FGFR1	fibroblast growth factor receptor 1	2260	ENSG00000077782
DSP	desmoplakin	1832	ENSG00000096696
CHRM5	cholinergic receptor, muscarinic 5	1133	ENSG00000184984
CEACAM6	carcinoembryonic antigen-related cell adhesion molecule 6 (non-specific cross reacting antigen)	4680	ENSG00000086548
NMS	neuromedin S	129521	ENSG00000204640
ZNF248	zinc finger protein 248	57209	ENSG00000198105
PTPRE	protein tyrosine phosphatase, receptor type, E	5791	ENSG00000132334
NSMCE2	non-SMC element 2, MMS21 homolog (S. cerevisiae)	286053	ENSG00000156831
CLCNKA	chloride channel, voltage-sensitive Ka	1187	ENSG00000186510
TTC37	tetratricopeptide repeat domain 37	9652	ENSG00000198677
MAGEC3	melanoma antigen family C, 3	139081	ENSG00000165509
LOC100996307	uncharacterized LOC100996307	100996307	NA
TBC1D3H	TBC1 domain family, member 3H	729877	ENSG00000242384
SDHB	succinate dehydrogenase complex, subunit B, iron sulfur (Ip)	6390	ENSG00000117118
KCTD15	potassium channel tetramerisation domain containing 15	79047	ENSG00000153885
OSBPL8	oxysterol binding protein-like 8	114882	ENSG00000091039
KNDC1	kinase non-catalytic C-lobe domain (KIND) containing 1	85442	ENSG00000171798
TMEM174	transmembrane protein 174	134288	ENSG00000164325
IGSF3	immunoglobulin superfamily, member 3	3321	ENSG00000143061
LONP1	lon peptidase 1, mitochondrial	9361	ENSG00000196365
MYO3B	myosin IIIB	140469	ENSG00000071909
AKD1	adenylate kinase domain containing 1	221264	ENSG00000155085
USP20	ubiquitin specific peptidase 20	10868	ENSG00000136878
HOXA13	homeobox A13	3209	ENSG00000106031
TEAD1	TEA domain family member 1 (SV40 transcriptional enhancer factor)	7003	ENSG00000187079
SORL1	sortilin-related receptor, L(DLR class) A repeats containing	6653	ENSG00000137642
NRP1	neuropilin 1	8829	ENSG00000099250
TUSC5	tumor suppressor candidate 5	286753	ENSG00000184811
B4GALNT4	beta-1,4-N-acetyl-galactosaminyl transferase 4	338707	ENSG00000182272
SLC25A14	solute carrier family 25 (mitochondrial carrier, brain), member 14	9016	ENSG00000102078
DMC1	DMC1 dosage suppressor of mck1 homolog, meiosis-specific homologous recombination (yeast)	11144	ENSG00000100206
PKD1	polycystic kidney disease 1 (autosomal dominant)	5310	ENSG00000008710
GJA1	gap junction protein, alpha 1, 43kDa	2697	ENSG00000152661
ATG2B	autophagy related 2B	55102	ENSG00000066739

LHFPL2	lipoma HMGIC fusion partner-like 2	10184	ENSG00000145685
EPS8	epidermal growth factor receptor pathway substrate 8	2059	ENSG00000151491
ELOVL5	ELOVL fatty acid elongase 5	60481	ENSG00000012660
DIRC2	disrupted in renal carcinoma 2	84925	ENSG00000138463
CAMK1	calcium/calmodulin-dependent protein kinase I	8536	ENSG00000134072
RFC3	replication factor C (activator 1) 3, 38kDa	5983	ENSG00000133119
KIAA1522	KIAA1522	57648	ENSG00000162522
OR6B3	olfactory receptor, family 6, subfamily B, member 3	150681	ENSG00000178586
SLC39A14	solute carrier family 39 (zinc transporter), member 14	23516	ENSG00000104635
SLC12A4	solute carrier family 12 (potassium/chloride transporters), member 4	6560	ENSG00000124067
RABGGTA	Rab geranylgeranyltransferase, alpha subunit	5875	ENSG00000100949
NGRN	neugrin, neurite outgrowth associated	51335	ENSG00000182768
CDK15	cyclin-dependent kinase 15	65061	ENSG00000138395
LRFN5	leucine rich repeat and fibronectin type III domain containing 5	145581	ENSG00000165379
LINC00469	long intergenic non-protein coding RNA 469	283982	NA
GPR137B	G protein-coupled receptor 137B	7107	ENSG00000077585
LUZP1	leucine zipper protein 1	7798	ENSG00000169641
EFNB1	ephrin-B1	1947	ENSG00000090776
DIP2C	DIP2 disco-interacting protein 2 homolog C (Drosophila)	22982	ENSG00000151240
BAHCC1	BAH domain and coiled-coil containing 1	57597	ENSG00000171282
CLDN4	claudin 4	1364	ENSG00000189143
BCO2	beta-carotene oxygenase 2	83875	ENSG00000197580
ARHGEF7	Rho guanine nucleotide exchange factor (GEF) 7	8874	ENSG00000102606
SNIP1	Smad nuclear interacting protein 1	79753	ENSG00000163877
A2M	alpha-2-macroglobulin	2	ENSG00000175899
ACSL3	acyl-CoA synthetase long-chain family member 3	2181	ENSG00000123983
PIK3R5	phosphoinositide-3-kinase, regulatory subunit 5	23533	ENSG00000141506
LIN54	lin-54 homolog (C. elegans)	132660	ENSG00000189308
COL9A2	collagen, type IX, alpha 2	1298	ENSG00000049089
TMEM86A	transmembrane protein 86A	144110	ENSG00000151117
MPPE1	metallophosphoesterase 1	65258	ENSG00000154889
ROCK2	Rho-associated, coiled-coil containing protein kinase 2	9475	ENSG00000134318
SNX16	sorting nexin 16	64089	ENSG00000104497
KIAA1683	KIAA1683	80726	ENSG00000130518
ACACA	acetyl-CoA carboxylase alpha	31	ENSG00000132142
TBL3	transducin (beta)-like 3	10607	ENSG00000183751
PAIP2	poly(A) binding protein interacting protein 2	51247	ENSG00000120727
CYP2U1	cytochrome P450, family 2, subfamily U, polypeptide 1	113612	ENSG00000155016
FOXK2	forkhead box K2	3607	ENSG00000141568
NFATC1	nuclear factor of activated T-cells, cytoplasmic, calcineurin-dependent 1	4772	ENSG00000131196
DSCAML1	Down syndrome cell adhesion molecule like 1	57453	ENSG00000177103
ASB13	ankyrin repeat and SOCS box containing 13	79754	ENSG00000196372
PRDM9	PR domain containing 9	56979	ENSG00000164256
NT5M	5',3'-nucleotidase, mitochondrial	56953	ENSG00000205309
INTU	inturned planar cell polarity effector homolog (Drosophila)	27152	ENSG00000164066
PTPN11	protein tyrosine phosphatase, non-receptor type 11	5781	ENSG00000179295
PRDM2	PR domain containing 2, with ZNF domain	7799	ENSG00000116731
TMCO3	transmembrane and coiled-coil domains 3	55002	ENSG00000150403
LYPD4	LY6/PLAUR domain containing 4	147719	ENSG00000183103
TSSC1	tumor suppressing subtransferable candidate 1	7260	ENSG00000032389
PPP3CA	protein phosphatase 3, catalytic subunit, alpha isozyme	5530	ENSG00000138814
SLC1A7	solute carrier family 1 (glutamate transporter), member 7	6512	ENSG00000162383
GJB6	gap junction protein, beta 6, 30kDa	10804	ENSG00000121742
C6orf108	chromosome 6 open reading frame 108	10591	ENSG00000112667
OR10J5	olfactory receptor, family 10, subfamily J, member 5	127385	ENSG00000184155
ANO5	anoctamin 5	203859	ENSG00000171714
SHROOM1	shroom family member 1	134549	ENSG00000164403
GFPT2	glutamine-fructose-6-phosphate transaminase 2	9945	ENSG00000131459
DAB1	disabled homolog 1 (Drosophila)	1600	ENSG00000173406

KCNC1	potassium voltage-gated channel, Shaw-related subfamily, member 1	3746	ENSG00000129159
CACNB3	calcium channel, voltage-dependent, beta 3 subunit	784	ENSG00000167535
RFTN1	raftlin, lipid raft linker 1	23180	ENSG00000131378
EVI5L	ecotropic viral integration site 5-like	115704	ENSG00000142459
DYSF	dysferlin, limb girdle muscular dystrophy 2B (autosomal recessive)	8291	ENSG00000135636
GOLGA3	golgin A3	2802	ENSG00000090615
SCUBE3	signal peptide, CUB domain, EGF-like 3	222663	ENSG00000146197
ARHGAP39	Rho GTPase activating protein 39	80728	ENSG00000147799
ABR	active BCR-related	29	ENSG00000159842
BBS7	Bardet-Biedl syndrome 7	55212	ENSG00000138686
PEX14	peroxisomal biogenesis factor 14	5195	ENSG00000142655
KIAA2018	KIAA2018	205717	ENSG00000176542
RIPK4	receptor-interacting serine-threonine kinase 4	54101	ENSG00000183421
ASB10	ankyrin repeat and SOCS box containing 10	136371	ENSG00000146926
MEIS3P1	Meis homeobox 3 pseudogene 1	4213	NA
FYCO1	FYVE and coiled-coil domain containing 1	79443	ENSG00000163820
FBLIM1	filamin binding LIM protein 1	54751	ENSG00000162458
PODN	podocan	127435	ENSG00000174348
NPC2	Niemann-Pick disease, type C2	10577	ENSG00000119655
FGF17	fibroblast growth factor 17	8822	ENSG00000158815
SNTG1	syntrophin, gamma 1	54212	ENSG00000147481
A2ML1	alpha-2-macroglobulin-like 1	144568	ENSG00000166535
C1orf227	chromosome 1 open reading frame 227	149643	ENSG00000185523
PCYT1A	phosphate cytidylyltransferase 1, choline, alpha	5130	ENSG00000161217
CNTN4	contactin 4	152330	ENSG00000144619
LINC00173	long intergenic non-protein coding RNA 173	100287569	NA
CHST1	carbohydrate (keratan sulfate Gal-6) sulfotransferase 1	8534	ENSG00000175264
TM4SF4	transmembrane 4 L six family member 4	7104	ENSG00000169903
C5orf38	chromosome 5 open reading frame 38	153571	ENSG00000186493
XIRP1	xin actin-binding repeat containing 1	165904	ENSG00000168334
ALDH18A1	aldehyde dehydrogenase 18 family, member A1	5832	ENSG00000059573
DNAJA4	DnaJ (Hsp40) homolog, subfamily A, member 4	55466	ENSG00000140403
ATP11A	ATPase, class VI, type 11A	23250	ENSG00000068650
SRI	sorcin	6717	ENSG00000075142
RASGRF1	Ras protein-specific guanine nucleotide-releasing factor 1	5923	ENSG00000058335
HLA-E	major histocompatibility complex, class I, E	3133	ENSG00000204592
ALDH2	aldehyde dehydrogenase 2 family (mitochondrial)	217	ENSG00000111275
AIRE	autoimmune regulator	326	ENSG00000160224
WDR1	WD repeat domain 1	9948	ENSG00000071127
RFXAP	regulatory factor X-associated protein	5994	ENSG00000133111
RNF4	ring finger protein 4	6047	ENSG00000063978
GMPR	guanosine monophosphate reductase	2766	ENSG00000137198
PIN1	peptidylprolyl cis/trans isomerase, NIMA-interacting 1	5300	ENSG00000127445
ADAT2	adenosine deaminase, tRNA-specific 2	134637	ENSG00000189007
MAPK12	mitogen-activated protein kinase 12	6300	ENSG00000188130
BOP1	block of proliferation 1	23246	ENSG00000170727
PIWIL3	piwi-like 3 (Drosophila)	440822	ENSG00000184571
F2RL1	coagulation factor II (thrombin) receptor-like 1	2150	ENSG00000164251
DKFZp779M0652	uncharacterized DKFZp779M0652	374387	NA
PRDM16	PR domain containing 16	63976	ENSG00000142611
CHST15	carbohydrate (N-acetylgalactosamine 4-sulfate 6-O) sulfotransferase 15	51363	ENSG00000182022
FRMD5	FERM domain containing 5	84978	ENSG00000171877
SLC15A4	solute carrier family 15, member 4	121260	ENSG00000139370
C1orf216	chromosome 1 open reading frame 216	127703	ENSG00000142686
LEPRE1	leucine proline-enriched proteoglycan (leprecan) 1	64175	ENSG00000117385
EP400	E1A binding protein p400	57634	ENSG00000183495
GNPDA1	glucosamine-6-phosphate deaminase 1	10007	ENSG00000113552
TEFM	transcription elongation factor, mitochondrial	79736	ENSG00000172171

ZC3H3	zinc finger CCCH-type containing 3	23144	ENSG00000014164
TRAPPC9	trafficking protein particle complex 9	83696	ENSG00000167632
LAMP3	lysosomal-associated membrane protein 3	27074	ENSG00000078081
FABP5	fatty acid binding protein 5 (psoriasis-associated)	2171	ENSG00000164687
DDAH1	dimethylarginine dimethylaminohydrolase 1	23576	ENSG00000153904
DLX6-AS1	DLX6 antisense RNA 1	285987	NA
LOC728558	uncharacterized LOC728558	728558	NA
JTB	jumping translocation breakpoint	10899	ENSG00000143543
PLXNA1	plexin A1	5361	ENSG00000114554
YWHAQ	tyrosine 3-monooxygenase/tryptophan 5-monooxygenase activation protein, theta polypeptide	10971	ENSG00000134308
VAX2	ventral anterior homeobox 2	25806	ENSG00000116035
PPAP2A	phosphatidic acid phosphatase type 2A	8611	ENSG00000067113
FAM163A	family with sequence similarity 163, member A	148753	ENSG00000143340
FAM167B	family with sequence similarity 167, member B	84734	ENSG00000183615
BCL2	B-cell CLL/lymphoma 2	596	ENSG00000171791
CAMTA1	calmodulin binding transcription activator 1	23261	ENSG00000171735
MAP3K14	mitogen-activated protein kinase kinase kinase 14	9020	ENSG00000006062
KL	klotho	9365	ENSG00000133116
PPP1R8	protein phosphatase 1, regulatory subunit 8	5511	ENSG00000117751
P4HA3	prolyl 4-hydroxylase, alpha polypeptide III	283208	ENSG00000149380
C12orf50	chromosome 12 open reading frame 50	160419	ENSG00000165805
MAFK	v-maf musculoaponeurotic fibrosarcoma oncogene homolog K (avian)	7975	ENSG00000198517
UBLCP1	ubiquitin-like domain containing CTD phosphatase 1	134510	ENSG00000164332
SLC6A6	solute carrier family 6 (neurotransmitter transporter, taurine), member 6	6533	ENSG00000131389
SEC14L1	SEC14-like 1 (S. cerevisiae)	6397	ENSG00000129657
UHRF2	ubiquitin-like with PHD and ring finger domains 2, E3 ubiquitin protein ligase	115426	ENSG00000147854
NAALADL1	N-acetylated alpha-linked acidic dipeptidase-like 1	10004	ENSG00000168060
EMILIN1	elastin microfibril interfacer 1	11117	ENSG00000138080
C12orf75	chromosome 12 open reading frame 75	387882	ENSG00000235162
PSMD3	proteasome (prosome, macropain) 26S subunit, non-ATPase, 3	5709	ENSG00000108344
PXDN	peroxidasin homolog (Drosophila)	7837	ENSG00000130508
DCAF12	DDB1 and CUL4 associated factor 12	25853	ENSG00000198876
RHEB	Ras homolog enriched in brain	6009	ENSG00000106615
NKRF	NFKB repressing factor	55922	ENSG00000186416
CGB5	chorionic gonadotropin, beta polypeptide 5	93659	ENSG00000189052
TMPRSS11E	transmembrane protease, serine 11E	28983	ENSG00000087128
RAB35	RAB35, member RAS oncogene family	11021	ENSG00000111737
PPP1R9A	protein phosphatase 1, regulatory subunit 9A	55607	ENSG00000158528
LOC100130581	uncharacterized LOC100130581	100130581	NA
LMF1	lipase maturation factor 1	64788	ENSG00000103227
ERLIN2	ER lipid raft associated 2	11160	ENSG00000147475
TMEM233	transmembrane protein 233	387890	ENSG00000224982
COX8A	cytochrome c oxidase subunit VIIIA (ubiquitous)	1351	ENSG00000176340
TOMM34	translocase of outer mitochondrial membrane 34	10953	ENSG00000025772
SLC12A6	solute carrier family 12 (potassium/chloride transporters), member 6	9990	ENSG00000140199
PCGF6	polycomb group ring finger 6	84108	ENSG00000156374
EFHD1	EF-hand domain family, member D1	80303	ENSG00000115468
PPAP2C	phosphatidic acid phosphatase type 2C	8612	ENSG00000141934
HIST1H4G	histone cluster 1, H4g	8369	ENSG00000124578
C14orf132	chromosome 14 open reading frame 132	56967	NA
C17orf101	chromosome 17 open reading frame 101	79701	ENSG00000181396
CXorf56	chromosome X open reading frame 56	63932	ENSG00000018610
CRHBP	corticotropin releasing hormone binding protein	1393	ENSG00000145708
CHST11	carbohydrate (chondroitin 4) sulfotransferase 11	50515	ENSG00000171310
MYEF2	myelin expression factor 2	50804	ENSG00000104177
RNF39	ring finger protein 39	80352	ENSG00000204618

MMP15	matrix metallopeptidase 15 (membrane-inserted)	4324	ENSG00000102996
TSPYL4	TSPY-like 4	23270	ENSG00000187189
USP42	ubiquitin specific peptidase 42	84132	ENSG00000106346
COPZ1	coatomer protein complex, subunit zeta 1	22818	ENSG00000111481
ADAMTS9	ADAM metallopeptidase with thrombospondin type 1 motif, 9	56999	ENSG00000163638
TASP1	taspase, threonine aspartase, 1	55617	ENSG00000089123
RGS4	regulator of G-protein signaling 4	5999	ENSG00000117152
TMEM92	transmembrane protein 92	162461	ENSG00000167105
TMEM200A	transmembrane protein 200A	114801	ENSG00000164484
DDX18	DEAD (Asp-Glu-Ala-Asp) box polypeptide 18	8886	ENSG00000088205
KCNH2	potassium voltage-gated channel, subfamily H (eag-related), member 2	3757	ENSG00000055118
TBX2	T-box 2	6909	ENSG00000121068
ADCK4	aarF domain containing kinase 4	79934	ENSG00000123815
PLXNB1	plexin B1	5364	ENSG00000164050
FYB	FYN binding protein	2533	ENSG00000082074
USP10	ubiquitin specific peptidase 10	9100	ENSG00000103194
SND1	staphylococcal nuclease and tudor domain containing 1	27044	ENSG00000197157
IRS1	insulin receptor substrate 1	3667	ENSG00000169047
ZNF24	zinc finger protein 24	7572	ENSG00000172466
EMBP1	embigin pseudogene 1	647121	NA
RHOH	ras homolog family member H	399	ENSG00000168421
NUCB1	nucleobindin 1	4924	ENSG00000104805
LRFN3	leucine rich repeat and fibronectin type III domain containing 3	79414	ENSG00000126243
TACR1	tachykinin receptor 1	6869	ENSG00000115353
FRAT1	frequently rearranged in advanced T-cell lymphomas	10023	ENSG00000165879
RHBDF1	rhomboid 5 homolog 1 (Drosophila)	64285	NA
IGSF11	immunoglobulin superfamily, member 11	152404	ENSG00000144847
RYR1	ryanodine receptor 1 (skeletal)	6261	ENSG00000196218
LAGE3	L antigen family, member 3	8270	ENSG00000196976
FGF8	fibroblast growth factor 8 (androgen-induced)	2253	ENSG00000107831
CHST6	carbohydrate (N-acetylglucosamine 6-O) sulfotransferase 6	4166	ENSG00000183196
VSNL1	visinin-like 1	7447	ENSG00000163032
DLGAP4	discs, large (Drosophila) homolog-associated protein 4	22839	ENSG00000080845
HAUS6	HAUS augmin-like complex, subunit 6	54801	ENSG00000147874
MYO5C	myosin VC	55930	ENSG00000128833
CDC25C	cell division cycle 25 homolog C (S. pombe)	995	ENSG00000158402
ZNF311	zinc finger protein 311	282890	ENSG00000197935
ACOT7	acyl-CoA thioesterase 7	11332	ENSG00000097021
SPICE1	spindle and centriole associated protein 1	152185	ENSG00000163611
PPEF2	protein phosphatase, EF-hand calcium binding domain 2	5470	ENSG00000156194
RASSF5	Ras association (RalGDS/AF-6) domain family member 5	83593	ENSG00000136653
DHX30	DEAH (Asp-Glu-Ala-His) box polypeptide 30	22907	ENSG00000132153
ABI2	abl-interactor 2	10152	ENSG00000138443
EHMT2	euchromatic histone-lysine N-methyltransferase 2	10919	ENSG00000204371
NUDT12	nudix (nucleoside diphosphate linked moiety X)-type motif 12	83594	ENSG00000112874
NCAM1	neural cell adhesion molecule 1	4684	ENSG00000149294
SLC38A4	solute carrier family 38, member 4	55089	ENSG00000139209
LIN52	lin-52 homolog (C. elegans)	91750	ENSG00000205659
PDP1	pyruvate dehyrogenase phosphatase catalytic subunit 1	54704	ENSG00000164951
FLNC	filamin C, gamma	2318	ENSG00000128591
CLSTN2	calsyntenin 2	64084	ENSG00000158258
RPL32	ribosomal protein L32	6161	ENSG00000144713
CCNG2	cyclin G2	901	ENSG00000138764
GNG12	guanine nucleotide binding protein (G protein), gamma 12	55970	ENSG00000172380
OPRK1	opioid receptor, kappa 1	4986	ENSG00000082556
IL6R	interleukin 6 receptor	3570	ENSG00000160712
PLD1	phospholipase D1, phosphatidylcholine-specific	5337	ENSG00000075651
PPARG	peroxisome proliferator-activated receptor gamma	5468	ENSG00000132170
GALR1	galanin receptor 1	2587	ENSG00000166573

CHRNB2	cholinergic receptor, nicotinic, beta 2 (neuronal)	1141	ENSG00000160716
UFL1	UFM1-specific ligase 1	23376	ENSG00000014123
FBRSL1	fibrosin-like 1	57666	ENSG00000112787
SEZ6	seizure related 6 homolog (mouse)	124925	ENSG00000063015
CD53	CD53 molecule	963	ENSG00000143119
PPP2R1B	protein phosphatase 2, regulatory subunit A, beta	5519	ENSG00000137713
TFAP2B	transcription factor AP-2 beta (activating enhancer binding protein 2 beta)	7021	ENSG00000008196
TANC1	tetratricopeptide repeat, ankyrin repeat and coiled-coil containing 1	85461	ENSG00000115183
HIST1H2AL	histone cluster 1, H2al	8332	ENSG00000198374
IRF6	interferon regulatory factor 6	3664	ENSG00000117595
GLG1	golgi glycoprotein 1	2734	ENSG00000090863
CIITA	class II, major histocompatibility complex, transactivator	4261	ENSG00000179583
SLC10A4	solute carrier family 10 (sodium/bile acid cotransporter family), member 4	201780	ENSG00000145248
CYP4F8	cytochrome P450, family 4, subfamily F, polypeptide 8	11283	ENSG00000186526
EPHA4	EPH receptor A4	2043	ENSG00000116106
FANCC	Fanconi anemia, complementation group C	2176	ENSG00000158169
CMTM3	CKLF-like MARVEL transmembrane domain containing 3	123920	ENSG00000140931
CAPN9	calpain 9	10753	ENSG00000135773
C18orf63	chromosome 18 open reading frame 63	644041	ENSG00000206043
SLC1A5	solute carrier family 1 (neutral amino acid transporter), member 5	6510	ENSG00000105281
MRPL15	mitochondrial ribosomal protein L15	29088	ENSG00000137547
LYSMD2	LysM, putative peptidoglycan-binding, domain containing 2	256586	ENSG00000140280
ARAP1	ArfGAP with RhoGAP domain, ankyrin repeat and PH domain 1	116985	ENSG00000186635
TMPRSS6	transmembrane protease, serine 6	164656	ENSG00000187045
HIST1H1A	histone cluster 1, H1a	3024	ENSG00000124610
DTX1	deltex homolog 1 (Drosophila)	1840	ENSG00000135144
TNNC1	troponin C type 1 (slow)	7134	ENSG00000114854
LOC285441	uncharacterized LOC285441	285441	NA
ANTXR2	anthrax toxin receptor 2	118429	ENSG00000163297
SLC6A18	solute carrier family 6, member 18	348932	ENSG00000164363
ADAMTS2	ADAM metallopeptidase with thrombospondin type 1 motif, 2	9509	ENSG00000087116
NPHS1	nephrosis 1, congenital, Finnish type (nephrin)	4868	ENSG00000161270
TSN	translin	7247	ENSG00000211460
TMEM74	transmembrane protein 74	157753	ENSG00000164841
SPRY2	sprouty homolog 2 (Drosophila)	10253	ENSG00000136158
PRSS56	protease, serine, 56	646960	NA
COLEC11	collectin sub-family member 11	78989	ENSG00000118004
HDHD2	haloacid dehalogenase-like hydrolase domain containing 2	84064	ENSG00000167220
PDZD9	PDZ domain containing 9	255762	ENSG00000155714
TTBK2	tau tubulin kinase 2	146057	ENSG00000128881
PLXNC1	plexin C1	10154	ENSG00000136040
SLC9A3	solute carrier family 9, subfamily A (NHE3, cation proton antiporter 3), member 3	6550	ENSG00000066230
AGTPBP1	ATP/GTP binding protein 1	23287	ENSG00000135049
TMEM230	transmembrane protein 230	29058	ENSG00000089063
PCSK6	proprotein convertase subtilisin/kexin type 6	5046	ENSG00000140479
SPRR2B	small proline-rich protein 2B	6701	ENSG00000196805
POGK	pogo transposable element with KRAB domain	57645	ENSG00000143157
THSD1	thrombospondin, type I, domain containing 1	55901	ENSG00000136114
ESRRG	estrogen-related receptor gamma	2104	ENSG00000196482
CNGA1	cyclic nucleotide gated channel alpha 1	1259	ENSG00000198515
C14orf166B	chromosome 14 open reading frame 166B	145497	ENSG00000100565
OTX1	orthodenticle homeobox 1	5013	ENSG00000115507
NYX	nyctalopin	60506	ENSG00000188937
HDAC9	histone deacetylase 9	9734	ENSG00000048052
HOXA3	homeobox A3	3200	ENSG00000105997
C1orf21	chromosome 1 open reading frame 21	81563	ENSG00000116667
TOM1L2	target of myb1-like 2 (chicken)	146691	ENSG00000175662

FAM108A1	family with sequence similarity 108, member A1	81926	ENSG00000129968
MLN	motilin	4295	ENSG00000096395
TIAM1	T-cell lymphoma invasion and metastasis 1	7074	ENSG00000156299
AHNAK	AHNAK nucleoprotein	79026	ENSG00000124942
RGS14	regulator of G-protein signaling 14	10636	ENSG00000169220
TUBA1B	tubulin, alpha 1b	10376	ENSG00000123416
ZIC4	Zic family member 4	84107	ENSG00000174963
ZNF566	zinc finger protein 566	84924	ENSG00000186017
PPP1R37	protein phosphatase 1, regulatory subunit 37	284352	ENSG00000104866
RBPMS	RNA binding protein with multiple splicing	11030	ENSG00000157110
RDH8	retinol dehydrogenase 8 (all-trans)	50700	ENSG00000080511
PHACTR1	phosphatase and actin regulator 1	221692	ENSG00000112137
CAMSAP1	calmodulin regulated spectrin-associated protein 1	157922	ENSG00000130559
PLAGL1	pleiomorphic adenoma gene-like 1	5325	ENSG00000118495
ATP1A1	ATPase, Na+/K+ transporting, alpha 1 polypeptide	476	ENSG00000163399
PIM1	pim-1 oncogene	5292	ENSG00000137193
GOLGA1	golgin A1	2800	ENSG00000136935
MIR487A	microRNA 487a	619555	NA
EML5	echinoderm microtubule associated protein like 5	161436	ENSG00000165521
SORCS2	sortilin-related VPS10 domain containing receptor 2	57537	ENSG00000184985
NEK7	NIMA (never in mitosis gene a)-related kinase 7	140609	ENSG00000151414
GAA	glucosidase, alpha; acid	2548	ENSG00000171298
ZSCAN10	zinc finger and SCAN domain containing 10	84891	ENSG00000130182
BRF1	BRF1 homolog, subunit of RNA polymerase III transcription initiation factor IIIB (S. cerevisiae)	2972	ENSG00000185024
GNAO1	guanine nucleotide binding protein (G protein), alpha activating activity polypeptide O	2775	ENSG00000087258
BTK	Bruton agammaglobulinemia tyrosine kinase	695	ENSG00000010671
TRAF7	TNF receptor-associated factor 7, E3 ubiquitin protein ligase	84231	ENSG00000131653
TMEM171	transmembrane protein 171	134285	ENSG00000157111
PIGL	phosphatidylinositol glycan anchor biosynthesis, class L	9487	ENSG00000108474
MED18	mediator complex subunit 18	54797	ENSG00000130772
ZC3HAV1	zinc finger CCCH-type, antiviral 1	56829	ENSG00000105939
WNT3A	wingless-type MMTV integration site family, member 3A	89780	ENSG00000154342
ARHGAP28	Rho GTPase activating protein 28	79822	ENSG00000088756
CDHR1	cadherin-related family member 1	92211	ENSG00000148600
RRAS2	related RAS viral (r-ras) oncogene homolog 2	22800	ENSG00000133818
CCDC109B	coiled-coil domain containing 109B	55013	ENSG00000005059
LTBP2	latent transforming growth factor beta binding protein 2	4053	ENSG00000119681
FAM181B	family with sequence similarity 181, member B	220382	ENSG00000182103
GABBR1	gamma-aminobutyric acid (GABA) B receptor, 1	2550	ENSG00000204681
OSBPL10	oxysterol binding protein-like 10	114884	ENSG00000144645
KCNJ6	potassium inwardly-rectifying channel, subfamily J, member 6	3763	ENSG00000157542
KCNK10	potassium channel, subfamily K, member 10	54207	ENSG00000100433
OTOA	otoancorin	146183	ENSG00000155719
AHRR	aryl-hydrocarbon receptor repressor	57491	ENSG00000063438
SLC25A22	solute carrier family 25 (mitochondrial carrier: glutamate), member 22	79751	ENSG00000177542
SNAI1	snail homolog 1 (Drosophila)	6615	ENSG00000124216
CCDC6	coiled-coil domain containing 6	8030	ENSG00000108091
POLRMT	polymerase (RNA) mitochondrial (DNA directed)	5442	ENSG00000099821
CD151	CD151 molecule (Raph blood group)	977	ENSG00000177697
PEAK1	NKF3 kinase family member	79834	ENSG00000173517
PHF21B	PHD finger protein 21B	112885	ENSG00000056487
MAPK14	mitogen-activated protein kinase 14	1432	ENSG00000112062
PLA2G6	phospholipase A2, group VI (cytosolic, calcium-independent)	8398	ENSG00000184381
DYNLL2	dynein, light chain, LC8-type 2	140735	ENSG00000121083
VWA5B1	von Willebrand factor A domain containing 5B1	127731	ENSG00000158816
SOX9	SRY (sex determining region Y)-box 9	6662	ENSG00000125398
GJB5	gap junction protein, beta 5, 31.1kDa	2709	ENSG00000189280
TRPV2	transient receptor potential cation channel, subfamily V, member 2	51393	ENSG00000187688

PLA1A	phospholipase A1 member A	51365	ENSG00000144837
CAMKK1	calcium/calmodulin-dependent protein kinase kinase 1, alpha	84254	ENSG00000004660
SEC63	SEC63 homolog (S. cerevisiae)	11231	ENSG00000025796
PPP1R42	protein phosphatase 1, regulatory subunit 42	286187	ENSG00000178125
FTH1	ferritin, heavy polypeptide 1	2495	ENSG00000167996
SATB2	SATB homeobox 2	23314	ENSG00000119042
FAF1	Fas (TNFRSF6) associated factor 1	11124	ENSG00000185104
MST1P9	macrophage stimulating 1 (hepatocyte growth factor-like) pseudogene 9	11223	NA
ANXA6	annexin A6	309	ENSG00000197043
CDK6	cyclin-dependent kinase 6	1021	ENSG00000105810
TNRC18	trinucleotide repeat containing 18	84629	ENSG00000182095
KIAA1908	uncharacterized LOC114796	114796	NA
HMX2	H6 family homeobox 2	3167	ENSG00000188816
ABTB2	ankyrin repeat and BTB (POZ) domain containing 2	25841	ENSG00000166016
EIF2S3	eukaryotic translation initiation factor 2, subunit 3 gamma, 52kDa	1968	ENSG00000130741
SNTG2	syntrophin, gamma 2	54221	ENSG00000172554
MUC5B	mucin 5B, oligomeric mucus/gel-forming	727897	ENSG00000117983
GABRB1	gamma-aminobutyric acid (GABA) A receptor, beta 1	2560	ENSG00000163288
FOXL1	forkhead box L1	2300	ENSG00000176678
MORC3	MORC family CW-type zinc finger 3	23515	ENSG00000159256
SVIL	supervillin	6840	ENSG00000197321
COL6A2	collagen, type VI, alpha 2	1292	ENSG00000142173
TMEM132C	transmembrane protein 132C	92293	ENSG00000181234
MUC12	mucin 12, cell surface associated	10071	ENSG00000205277
FBLN1	fibulin 1	2192	ENSG00000077942
MYOF	myoferlin	26509	ENSG00000138119
CAMK2B	calcium/calmodulin-dependent protein kinase II beta	816	ENSG00000058404
NTM	neurotrimin	50863	ENSG00000182667
GATS	GATS, stromal antigen 3 opposite strand	352954	ENSG00000160844
GRIP1	glutamate receptor interacting protein 1	23426	ENSG00000155974
DDX39A	DEAD (Asp-Glu-Ala-Asp) box polypeptide 39A	10212	ENSG00000123136
SUB1	SUB1 homolog (S. cerevisiae)	10923	ENSG00000113387
INF2	inverted formin, FH2 and WH2 domain containing	64423	ENSG00000203485
STC1	stanniocalcin 1	6781	ENSG00000159167
ASAP1	ArfGAP with SH3 domain, ankyrin repeat and PH domain 1	50807	ENSG00000153317
SGSM1	small G protein signaling modulator 1	129049	ENSG00000167037
FAM138D	family with sequence similarity 138, member D	677784	NA
LYST	lysosomal trafficking regulator	1130	ENSG00000143669
NGFR	nerve growth factor receptor	4804	ENSG00000064300
MEIS1	Meis homeobox 1	4211	ENSG00000143995
ATP8B4	ATPase, class I, type 8B, member 4	79895	ENSG00000104043
MPV17L2	MPV17 mitochondrial membrane protein-like 2	84769	ENSG00000254858
DPCR1	diffuse panbronchiolitis critical region 1	135656	ENSG00000168631
NSD1	nuclear receptor binding SET domain protein 1	64324	ENSG00000165671
SEC13	SEC13 homolog (S. cerevisiae)	6396	ENSG00000157020
R3HDM2	R3H domain containing 2	22864	ENSG00000179912
ANKRD26	ankyrin repeat domain 26	22852	ENSG00000107890
PDGFD	platelet derived growth factor D	80310	ENSG00000170962
CACNA1H	calcium channel, voltage-dependent, T type, alpha 1H subunit	8912	ENSG00000196557
PDE8A	phosphodiesterase 8A	5151	ENSG00000073417
MYO1H	myosin IH	283446	ENSG00000174527
YPEL5	yippee-like 5 (Drosophila)	51646	ENSG00000119801
SH3KBP1	SH3-domain kinase binding protein 1	30011	ENSG00000147010
TRIL	TLR4 interactor with leucine-rich repeats	9865	NA
MTMR12	myotubularin related protein 12	54545	ENSG00000150712
CREBBP	CREB binding protein	1387	ENSG00000005339
ITGB1	integrin, beta 1 (fibronectin receptor, beta polypeptide, antigen CD29 includes MDF2, MSK12)	3688	ENSG00000150093
MIR100HG	mir-100-let-7a-2 cluster host gene (non-protein coding)	399959	NA

LINC00629	long intergenic non-protein coding RNA 629	100506757	NA
LARP1	La ribonucleoprotein domain family, member 1	23367	ENSG00000155506
GATAD1	GATA zinc finger domain containing 1	57798	ENSG00000157259
RPN1	ribophorin I	6184	ENSG00000163902
RIMBP2	RIMS binding protein 2	23504	ENSG00000060709
ABCC2	ATP-binding cassette, sub-family C (CFTR/MRP), member 2	1244	ENSG00000023839
SIX2	SIX homeobox 2	10736	ENSG00000170577
TTC12	tetratricopeptide repeat domain 12	54970	ENSG00000149292
FAM161A	family with sequence similarity 161, member A	84140	ENSG00000170264
VIM	vimentin	7431	ENSG00000026025
PSKH2	protein serine kinase H2	85481	ENSG00000147613
CADPS	Ca++-dependent secretion activator	8618	ENSG00000163618
LTB	lymphotoxin beta (TNF superfamily, member 3)	4050	ENSG00000227507
ZFPM1	zinc finger protein, multitype 1	161882	ENSG00000179588
DES	desmin	1674	ENSG00000175084
LGALS3BP	lectin, galactoside-binding, soluble, 3 binding protein	3959	ENSG00000108679
HLA-DOA	major histocompatibility complex, class II, DO alpha	3111	ENSG00000204252
CSMD3	CUB and Sushi multiple domains 3	114788	ENSG00000164796
CLDND1	claudin domain containing 1	56650	ENSG00000080822
TANK	TRAF family member-associated NFKB activator	10010	ENSG00000136560
SPR	sepiapterin reductase (7,8-dihydrobiopterin:NADP+ oxidoreductase)	6697	ENSG00000116096
BAI1	brain-specific angiogenesis inhibitor 1	575	ENSG00000181790
PCDHB2	protocadherin beta 2	56133	ENSG00000112852
GPATCH4	G patch domain containing 4	54865	ENSG00000160818
CYB5R4	cytochrome b5 reductase 4	51167	ENSG00000065615
ADAMTS4	ADAM metallopeptidase with thrombospondin type 1 motif, 4	9507	ENSG00000158859
MAML3	mastermind-like 3 (Drosophila)	55534	ENSG00000196782
SOLH	small optic lobes homolog (Drosophila)	6650	ENSG00000103326
RGS7	regulator of G-protein signaling 7	6000	ENSG00000182901
ENO4	enolase family member 4	387712	ENSG00000188316
MAP7	microtubule-associated protein 7	9053	ENSG00000135525
FHIT	fragile histidine triad	2272	ENSG00000189283
FER1L4	fer-1-like 4 (C. elegans) pseudogene	80307	NA
ADAMTS14	ADAM metallopeptidase with thrombospondin type 1 motif, 14	140766	ENSG00000138316
COX20	COX20 Cox2 chaperone homolog (S. cerevisiae)	116228	ENSG00000203667
TM9SF4	transmembrane 9 superfamily protein member 4	9777	ENSG00000101337
RCVRN	recoverin	5957	ENSG00000109047
NKX2-8	NK2 homeobox 8	26257	ENSG00000136327
HSPA6	heat shock 70kDa protein 6 (HSP70B')	3310	ENSG00000173110
EPB49	erythrocyte membrane protein band 4.9 (dematin)	2039	ENSG00000158856
HR	hairless homolog (mouse)	55806	ENSG00000168453
TTBK1	tau tubulin kinase 1	84630	ENSG00000146216
SCARNA2	small Cajal body-specific RNA 2	677766	NA
TERF2	telomeric repeat binding factor 2	7014	ENSG00000132604
CORO1C	coronin, actin binding protein, 1C	23603	ENSG00000110880
C7orf53	chromosome 7 open reading frame 53	286006	ENSG00000181016
CD8A	CD8a molecule	925	ENSG00000153563
ZCCHC7	zinc finger, CCHC domain containing 7	84186	ENSG00000147905
SYNJ2	synaptojanin 2	8871	ENSG00000078269
SLC25A29	solute carrier family 25 (mitochondrial carnitine/acylcarnitine carrier), member 29	123096	ENSG00000197119
PRRT1	proline-rich transmembrane protein 1	80863	ENSG00000204314
STK32A	serine/threonine kinase 32A	202374	ENSG00000169302
STARD3NL	STARD3 N-terminal like	83930	ENSG00000010270
TRIM26	tripartite motif containing 26	7726	ENSG00000234127
LRR1	leucine rich repeat protein 1	122769	ENSG00000165501
MED25	mediator complex subunit 25	81857	ENSG00000104973
XAF1	XIAP associated factor 1	54739	ENSG00000132530
MYH10	myosin, heavy chain 10, non-muscle	4628	ENSG00000133026
ARSB	arylsulfatase B	411	ENSG00000113273

PLA2G4C	phospholipase A2, group IVC (cytosolic, calcium-independent)	8605	ENSG00000105499
TTC39C	tetratricopeptide repeat domain 39C	125488	ENSG00000168234
FAM131C	family with sequence similarity 131, member C	348487	ENSG00000185519
F2	coagulation factor II (thrombin)	2147	ENSG00000180210
MAD1L1	MAD1 mitotic arrest deficient-like 1 (yeast)	8379	ENSG00000002822
BEND7	BEN domain containing 7	222389	ENSG00000165626
SPIN4	spindlin family, member 4	139886	ENSG00000186767
ZNF710	zinc finger protein 710	374655	ENSG00000140548
IL13RA1	interleukin 13 receptor, alpha 1	3597	ENSG00000131724
TBXAS1	thromboxane A synthase 1 (platelet)	6916	ENSG00000059377
EIF2B2	eukaryotic translation initiation factor 2B, subunit 2 beta, 39kDa	8892	ENSG00000119718
SPAG6	sperm associated antigen 6	9576	ENSG00000077327
GPR176	G protein-coupled receptor 176	11245	ENSG00000166073
KALRN	kalirin, RhoGEF kinase	8997	ENSG00000160145
TRABD2B	TraB domain containing 2B	388630	ENSG00000204018
TNK2	tyrosine kinase, non-receptor, 2	10188	ENSG00000061938
NOP16	NOP16 nucleolar protein homolog (yeast)	51491	ENSG00000048162
FOXR1	forkhead box R1	283150	ENSG00000176302
USP25	ubiquitin specific peptidase 25	29761	ENSG00000155313
ANKRD11	ankyrin repeat domain 11	29123	ENSG00000167522
DOPEY2	dopey family member 2	9980	ENSG00000142197
EBF3	early B-cell factor 3	253738	ENSG00000108001
KLHL35	kelch-like 35 (Drosophila)	283212	ENSG00000149243
SQLE	squalene epoxidase	6713	ENSG00000104549
LRP5	low density lipoprotein receptor-related protein 5	4041	ENSG00000162337
SEPHS1	selenophosphate synthetase 1	22929	ENSG00000086475
PLRG1	pleiotropic regulator 1	5356	ENSG00000171566
PCK1	phosphoenolpyruvate carboxykinase 1 (soluble)	5105	ENSG00000124253
FGF4	fibroblast growth factor 4	2249	ENSG00000075388
ARR3	arrestin 3, retinal (X-arrestin)	407	ENSG00000120500
PLEKHA7	pleckstrin homology domain containing, family A member 7	144100	ENSG00000166689
OLAH	oleoyl-ACP hydrolase	55301	ENSG00000152463
MYO5A	myosin VA (heavy chain 12, myoxin)	4644	ENSG00000197535
SPG7	spastic paraplegia 7 (pure and complicated autosomal recessive)	6687	ENSG00000197912
GPR45	G protein-coupled receptor 45	11250	ENSG00000135973
BCAR1	breast cancer anti-estrogen resistance 1	9564	ENSG00000050820
ATXN3	ataxin 3	4287	ENSG00000066427
PARD6G	par-6 partitioning defective 6 homolog gamma (C. elegans)	84552	ENSG00000178184
MAP4K3	mitogen-activated protein kinase kinase kinase kinase 3	8491	ENSG00000011566
LIPA	lipase A, lysosomal acid, cholesterol esterase	3988	ENSG00000107798
ADO	2-aminoethanethiol (cysteamine) dioxygenase	84890	ENSG00000181915
NAV2	neuron navigator 2	89797	ENSG00000166833
XXYLT1	xyloside xylosyltransferase 1	152002	ENSG00000173950
WNT9A	wingless-type MMTV integration site family, member 9A	7483	ENSG00000143816
TPGS1	tubulin polyglutamylase complex subunit 1	91978	ENSG00000141933
CLK3	CDC-like kinase 3	1198	ENSG00000179335
OXR1	oxidation resistance 1	55074	ENSG00000164830
MICA	MHC class I polypeptide-related sequence A	100507436	ENSG00000204520
GAMT	guanidinoacetate N-methyltransferase	2593	ENSG00000130005
ATXN7	ataxin 7	6314	ENSG00000163635
STK3	serine/threonine kinase 3	6788	ENSG00000104375
LOC339505	uncharacterized LOC339505	339505	NA
KRTAP6-3	keratin associated protein 6-3	337968	ENSG00000212938
H2AFY2	H2A histone family, member Y2	55506	ENSG00000099284
CLIP2	CAP-GLY domain containing linker protein 2	7461	ENSG00000106665
LOC100216001	uncharacterized LOC100216001	100216001	NA
CDH26	cadherin 26	60437	ENSG00000124215
SFMBT2	Scm-like with four mbt domains 2	57713	ENSG00000198879
CDC42	cell division cycle 42 (GTP binding protein, 25kDa)	998	ENSG00000070831

LHX8	LIM homeobox 8	431707	ENSG00000162624
GALNT9	UDP-N-acetyl-alpha-D-galactosamine:polypeptide N-acetylgalactosaminyltransferase 9 (GalNAc-T9)	50614	ENSG00000182870
CD97	CD97 molecule	976	ENSG00000123146
CRIM1	cysteine rich transmembrane BMP regulator 1 (chordin-like)	51232	ENSG00000150938
NINJ2	ninjurin 2	4815	ENSG00000171840
HHIPL1	HHIP-like 1	84439	ENSG00000182218
WDFY4	WDFY family member 4	57705	ENSG00000128815
MTIF3	mitochondrial translational initiation factor 3	219402	ENSG00000122033
CD93	CD93 molecule	22918	ENSG00000125810
NRG2	neuregulin 2	9542	ENSG00000158458
USP18	ubiquitin specific peptidase 18	11274	ENSG00000184979
KDM6B	lysine (K)-specific demethylase 6B	23135	ENSG00000132510
TAL2	T-cell acute lymphocytic leukemia 2	6887	ENSG00000186051
S100B	S100 calcium binding protein B	6285	ENSG00000160307
C7orf65	chromosome 7 open reading frame 65	401335	ENSG00000221845
CHDH	choline dehydrogenase	55349	ENSG00000016391
TRPC1	transient receptor potential cation channel, subfamily C, member 1	7220	ENSG00000144935
DNAJC1	DnaJ (Hsp40) homolog, subfamily C, member 1	64215	ENSG00000136770
KLF12	Kruppel-like factor 12	11278	ENSG00000118922
GABRG3	gamma-aminobutyric acid (GABA) A receptor, gamma 3	2567	ENSG00000182256
ASZ1	ankyrin repeat, SAM and basic leucine zipper domain containing 1	136991	ENSG00000154438
SURF4	surfeit 4	6836	ENSG00000148248
ITM2C	integral membrane protein 2C	81618	ENSG00000135916
PKNOX1	PBX/knotted 1 homeobox 1	5316	ENSG00000160199
PFKFB3	6-phosphofructo-2-kinase/fructose-2,6-biphosphatase 3	5209	ENSG00000170525
SIN3A	SIN3 transcription regulator homolog A (yeast)	25942	ENSG00000169375
ECSIT	ECSIT homolog (Drosophila)	51295	ENSG00000130159
VSIG4	V-set and immunoglobulin domain containing 4	11326	ENSG00000155659
CLCN7	chloride channel, voltage-sensitive 7	1186	ENSG00000103249
CCDC85B	coiled-coil domain containing 85B	11007	ENSG00000175602
TFF2	trefoil factor 2	7032	ENSG00000160181
DVL1	dishevelled, dsh homolog 1 (Drosophila)	1855	ENSG00000107404
WDR60	WD repeat domain 60	55112	ENSG00000126870
OTP	orthopedia homeobox	23440	ENSG00000171540
ITIH3	inter-alpha-trypsin inhibitor heavy chain 3	3699	ENSG00000162267
AGFG2	ArfGAP with FG repeats 2	3268	ENSG00000106351
THEGL	theg spermatid protein-like	100506564	ENSG00000249693
PLCXD3	phosphatidylinositol-specific phospholipase C, X domain containing 3	345557	ENSG00000182836
DENND2A	DENN/MADD domain containing 2A	27147	ENSG00000146966
CABLES2	Cdk5 and Abl enzyme substrate 2	81928	ENSG00000149679
DDA1	DET1 and DDB1 associated 1	79016	ENSG00000130311
ZSWIM6	zinc finger, SWIM-type containing 6	57688	ENSG00000130449
SLC5A9	solute carrier family 5 (sodium/glucose cotransporter), member 9	200010	ENSG00000117834
MAN2A1	mannosidase, alpha, class 2A, member 1	4124	ENSG00000112893
LOC100132891	uncharacterized LOC100132891	100132891	NA
SIK2	salt-inducible kinase 2	23235	ENSG00000170145
ZBTB47	zinc finger and BTB domain containing 47	92999	ENSG00000114853
SDK2	sidekick cell adhesion molecule 2	54549	ENSG00000069188
BAIAP3	BAI1-associated protein 3	8938	ENSG00000007516
HHIPL2	HHIP-like 2	79802	ENSG00000143512
CALY	calcyon neuron-specific vesicular protein	50632	ENSG00000130643
RND3	Rho family GTPase 3	390	ENSG00000115963
LRRC3C	leucine rich repeat containing 3C	100505591	ENSG00000204913
SSTR3	somatostatin receptor 3	6753	ENSG00000183473
IL1RAP	interleukin 1 receptor accessory protein	3556	ENSG00000196083
SPATS2L	spermatogenesis associated, serine-rich 2-like	26010	ENSG00000196141
LOC729444	uncharacterized LOC729444	729444	NA
VDAC1	voltage-dependent anion channel 1	7416	ENSG00000213585

PPIA	peptidylprolyl isomerase A (cyclophilin A)	5478	ENSG00000196262
NDOR1	NADPH dependent diflavin oxidoreductase 1	27158	ENSG00000188566
TRIB1	tribbles homolog 1 (Drosophila)	10221	ENSG00000173334
DLEU2	deleted in lymphocytic leukemia 2 (non-protein coding)	8847	NA
ESRRB	estrogen-related receptor beta	2103	ENSG00000119715
YPEL1	yippee-like 1 (Drosophila)	29799	ENSG00000100027
TPRN	taperin	286262	ENSG00000176058
KCNMB3	potassium large conductance calcium-activated channel, subfamily M beta member 3	27094	ENSG00000171121
CCNJL	cyclin J-like	79616	ENSG00000135083
PRKACB	protein kinase, cAMP-dependent, catalytic, beta	5567	ENSG00000142875
PDE3A	phosphodiesterase 3A, cGMP-inhibited	5139	ENSG00000172572
FRS3	fibroblast growth factor receptor substrate 3	10817	ENSG00000137218
ZNF536	zinc finger protein 536	9745	ENSG00000198597
NADKD1	NAD kinase domain containing 1	133686	ENSG00000152620
CAND2	cullin-associated and neddylation-dissociated 2 (putative)	23066	ENSG00000144712
BCL6	B-cell CLL/lymphoma 6	604	ENSG00000113916
RPH3AL	rabphilin 3A-like (without C2 domains)	9501	ENSG00000181031
CSMD1	CUB and Sushi multiple domains 1	64478	ENSG00000183117
FCHSD2	FCH and double SH3 domains 2	9873	ENSG00000137478
SLC6A20	solute carrier family 6 (proline IMINO transporter), member 20	54716	ENSG00000163817
LIMS1	LIM and senescent cell antigen-like domains 1	3987	ENSG00000169756
SGMS1	sphingomyelin synthase 1	259230	ENSG00000198964
TRERF1	transcriptional regulating factor 1	55809	ENSG00000124496
SETBP1	SET binding protein 1	26040	ENSG00000152217
ASB16	ankyrin repeat and SOCS box containing 16	92591	ENSG00000161664
CDH11	cadherin 11, type 2, OB-cadherin (osteoblast)	1009	ENSG00000140937
ADCY8	adenylate cyclase 8 (brain)	114	ENSG00000155897
ZNF653	zinc finger protein 653	115950	ENSG00000161914
NRSN1	neurensin 1	140767	ENSG00000152954
ADSSL1	adenylosuccinate synthase like 1	122622	ENSG00000185100
SLC31A2	solute carrier family 31 (copper transporters), member 2	1318	ENSG00000136867
MSANTD1	Myb/SANT-like DNA-binding domain containing 1	345222	ENSG00000188981
AP2S1	adaptor-related protein complex 2, sigma 1 subunit	1175	ENSG00000042753
ANO7	anoctamin 7	50636	ENSG00000146205
C3orf36	chromosome 3 open reading frame 36	80111	ENSG00000221972
C2orf69	chromosome 2 open reading frame 69	205327	ENSG00000178074
ZIC3	Zic family member 3	7547	ENSG00000156925
GRIN2A	glutamate receptor, ionotropic, N-methyl D-aspartate 2A	2903	ENSG00000183454
KANK4	KN motif and ankyrin repeat domains 4	163782	ENSG00000132854
RPL6	ribosomal protein L6	6128	ENSG00000089009
CEP72	centrosomal protein 72kDa	55722	ENSG00000112877
DDX46	DEAD (Asp-Glu-Ala-Asp) box polypeptide 46	9879	ENSG00000145833
SPRR1A	small proline-rich protein 1A	6698	ENSG00000169474
ZNF530	zinc finger protein 530	348327	ENSG00000183647
SCEL	sciellin	8796	ENSG00000136155
RUFY1	RUN and FYVE domain containing 1	80230	ENSG00000176783
LHPP	phospholysine phosphohistidine inorganic pyrophosphate phosphatase	64077	ENSG00000107902
ABHD16B	abhydrolase domain containing 16B	140701	ENSG00000183260
NTS	neurotensin	4922	ENSG00000133636
BCOR	BCL6 corepressor	54880	ENSG00000183337
SH3TC1	SH3 domain and tetratricopeptide repeats 1	54436	ENSG00000125089
TRIM51GP	tripartite motif-containing 51G, pseudogene	120824	NA
SAR1A	SAR1 homolog A (S. cerevisiae)	56681	ENSG00000079332
ASB1	ankyrin repeat and SOCS box containing 1	51665	ENSG00000065802
KCNK12	potassium channel, subfamily K, member 12	56660	ENSG00000184261
PTGIR	prostaglandin I2 (prostacyclin) receptor (IP)	5739	ENSG00000160013
UNKL	unkempt homolog (Drosophila)-like	64718	ENSG00000059145

TMEFF2	transmembrane protein with EGF-like and two follistatin-like domains 2	23671	ENSG00000144339
NLGN3	neuroligin 3	54413	ENSG00000196338
MUC4	mucin 4, cell surface associated	4585	ENSG00000145113
LMX1A	LIM homeobox transcription factor 1, alpha	4009	ENSG00000162761
MIAT	myocardial infarction associated transcript (non-protein coding)	440823	NA
NANP	N-acetylneuraminic acid phosphatase	140838	ENSG00000170191
IGSF5	immunoglobulin superfamily, member 5	150084	ENSG00000183067
OTUB2	OTU domain, ubiquitin aldehyde binding 2	78990	ENSG00000089723
NELL2	NEL-like 2 (chicken)	4753	ENSG00000184613
ELMOD2	ELMO/CED-12 domain containing 2	255520	ENSG00000179387
SHC3	SHC (Src homology 2 domain containing) transforming protein 3	53358	ENSG00000148082
CSNK1A1	casein kinase 1, alpha 1	1452	ENSG00000113712
ARID1B	AT rich interactive domain 1B (SWI1-like)	57492	ENSG00000049618
MTUS1	microtubule associated tumor suppressor 1	57509	ENSG00000129422
TBL2	transducin (beta)-like 2	26608	ENSG00000106638
MMEL1	membrane metallo-endopeptidase-like 1	79258	ENSG00000142606
DPY19L2P2	dpy-19-like 2 pseudogene 2 (C. elegans)	349152	NA
KRI1	KRI1 homolog (S. cerevisiae)	65095	ENSG00000129347
MGMT	O-6-methylguanine-DNA methyltransferase	4255	ENSG00000170430
TNFRSF13C	tumor necrosis factor receptor superfamily, member 13C	115650	ENSG00000159958
PIEZO1	piezo-type mechanosensitive ion channel component 1	9780	ENSG00000103335
PCSK9	proprotein convertase subtilisin/kexin type 9	255738	ENSG00000169174
MAGI1	membrane associated guanylate kinase, WW and PDZ domain containing 1	9223	ENSG00000151276
KIAA0087	KIAA0087	9808	NA
ZFP82	zinc finger protein 82 homolog (mouse)	284406	ENSG00000181007
CNOT10	CCR4-NOT transcription complex, subunit 10	25904	ENSG00000182973
DIMT1	DIM1 dimethyladenosine transferase 1 homolog (S. cerevisiae)	27292	ENSG00000086189
ABT1	activator of basal transcription 1	29777	ENSG00000146109
ANKRD9	ankyrin repeat domain 9	122416	ENSG00000156381
SLC14A2	solute carrier family 14 (urea transporter), member 2	8170	ENSG00000132874
MDFI	MyoD family inhibitor	4188	ENSG00000112559
C1orf220	chromosome 1 open reading frame 220	400798	NA
BTBD8	BTB (POZ) domain containing 8	284697	ENSG00000189195
LAMA5	laminin, alpha 5	3911	ENSG00000130702
TMEM184B	transmembrane protein 184B	25829	ENSG00000198792
LOC100287010	uncharacterized LOC100287010	100287010	NA
EIF3H	eukaryotic translation initiation factor 3, subunit H	8667	ENSG00000147677
KIF1B	kinesin family member 1B	23095	ENSG00000054523
ARHGAP23	Rho GTPase activating protein 23	57636	ENSG00000225485
MYO7B	myosin VIIB	4648	ENSG00000169994
GPT	glutamic-pyruvate transaminase (alanine aminotransferase)	2875	ENSG00000167701
ZNF267	zinc finger protein 267	10308	ENSG00000185947
FBXL19	F-box and leucine-rich repeat protein 19	54620	ENSG00000099364
OSGIN2	oxidative stress induced growth inhibitor family member 2	734	ENSG00000164823
PSMC1	proteasome (prosome, macropain) 26S subunit, ATPase, 1	5700	ENSG00000100764
ALKBH7	alkB, alkylation repair homolog 7 (E. coli)	84266	ENSG00000125652
AMPD3	adenosine monophosphate deaminase 3	272	ENSG00000133805
SNX29	sorting nexin 29	92017	ENSG00000048471
FBXO7	F-box protein 7	25793	ENSG00000100225
LRRFIP1	leucine rich repeat (in FLII) interacting protein 1	9208	ENSG00000124831
HYAL2	hyaluronoglucosaminidase 2	8692	ENSG00000068001
TAF6L	TAF6-like RNA polymerase II, p300/CBP-associated factor (PCAF)-associated factor, 65kDa	10629	ENSG00000162227
SEC61A1	Sec61 alpha 1 subunit (S. cerevisiae)	29927	ENSG00000058262
SRSF8	serine/arginine-rich splicing factor 8	10929	NA
LINC00094	long intergenic non-protein coding RNA 94	266655	NA
TFG	TRK-fused gene	10342	ENSG00000114354

HMGB2	high mobility group box 2	3148	ENSG00000164104
ERGIC3	ERGIC and golgi 3	51614	ENSG00000125991
CYMP	chymosin pseudogene	643160	NA
ONECUT3	one cut homeobox 3	390874	ENSG00000205922
MEGF6	multiple EGF-like-domains 6	1953	ENSG00000162591
GABRA1	gamma-aminobutyric acid (GABA) A receptor, alpha 1	2554	ENSG00000022355
C3orf27	chromosome 3 open reading frame 27	23434	ENSG00000198685
GALNT10	UDP-N-acetyl-alpha-D-galactosamine:polypeptide N-acetylgalactosaminyltransferase 10 (GalNAc-T10)	55568	ENSG00000164574
HTR3D	5-hydroxytryptamine (serotonin) receptor 3D, ionotropic	200909	ENSG00000186090
VIPR2	vasoactive intestinal peptide receptor 2	7434	ENSG00000106018
HEATR7B2	HEAT repeat family member 7B2	133558	ENSG00000171495
CREM	cAMP responsive element modulator	1390	ENSG00000095794
KCNQ1	potassium voltage-gated channel, KQT-like subfamily, member 1	3784	ENSG00000053918
MAST1	microtubule associated serine/threonine kinase 1	22983	ENSG00000105613
LIMK2	LIM domain kinase 2	3985	ENSG00000182541
HMGA2	high mobility group AT-hook 2	8091	ENSG00000149948
ADCY1	adenylate cyclase 1 (brain)	107	ENSG00000164742
RPS6KA2	ribosomal protein S6 kinase, 90kDa, polypeptide 2	6196	ENSG00000071242
ZNF558	zinc finger protein 558	148156	ENSG00000167785
C20orf112	chromosome 20 open reading frame 112	140688	ENSG00000197183
BSG	basigin (Ok blood group)	682	ENSG00000172270
PARP8	poly (ADP-ribose) polymerase family, member 8	79668	ENSG00000151883
LYPD1	LY6/PLAUR domain containing 1	116372	ENSG00000150551
ISG15	ISG15 ubiquitin-like modifier	9636	ENSG00000187608
TEAD4	TEA domain family member 4	7004	ENSG00000197905
DNAH2	dynein, axonemal, heavy chain 2	146754	ENSG00000183914
KCNQ1DN	KCNQ1 downstream neighbor (non-protein coding)	55539	NA
LOC728739	programmed cell death 2 pseudogene	728739	NA
LOC643623	uncharacterized LOC643623	643623	NA
FGFBP1	fibroblast growth factor binding protein 1	9982	ENSG00000137440
CCDC68	coiled-coil domain containing 68	80323	ENSG00000166510
HLA-DRB6	major histocompatibility complex, class II, DR beta 6 (pseudogene)	3128	NA
CYTH4	cytohesin 4	27128	ENSG00000100055
SLC25A30	solute carrier family 25, member 30	253512	ENSG00000174032
TAAR6	trace amine associated receptor 6	319100	ENSG00000146383
FBRS	fibrosin	64319	ENSG00000156860
CCRL2	chemokine (C-C motif) receptor-like 2	9034	ENSG00000121797
DOCK6	dedicator of cytokinesis 6	57572	ENSG00000130158
DGKK	diacylglycerol kinase, kappa	139189	NA
PCDHB6	protocadherin beta 6	56130	ENSG00000113211
FMNL1	formin-like 1	752	ENSG00000184922
GLCCI1	glucocorticoid induced transcript 1	113263	ENSG00000106415
ACAP1	ArfGAP with coiled-coil, ankyrin repeat and PH domains 1	9744	ENSG00000072818
LHX1	LIM homeobox 1	3975	ENSG00000132130
EIF4B	eukaryotic translation initiation factor 4B	1975	ENSG00000063046
FAM83A	family with sequence similarity 83, member A	84985	ENSG00000147689
ANKRD65	ankyrin repeat domain 65	441869	ENSG00000235098
NUP35	nucleoporin 35kDa	129401	ENSG00000163002
MCF2L	MCF.2 cell line derived transforming sequence-like	23263	ENSG00000126217
ZNF213	zinc finger protein 213	7760	ENSG00000085644
SPEG	SPEG complex locus	10290	ENSG00000072195
PGM2	phosphoglucomutase 2	55276	ENSG00000169299
CLNK	cytokine-dependent hematopoietic cell linker	116449	ENSG00000109684
LINC00521	long intergenic non-protein coding RNA 521	256369	NA
KIAA1045	KIAA1045	23349	ENSG00000122733
ABCD3	ATP-binding cassette, sub-family D (ALD), member 3	5825	ENSG00000117528
HERC1	HECT and RLD domain containing E3 ubiquitin protein ligase family member 1	8925	ENSG00000103657
ARSI	arylsulfatase family, member I	340075	ENSG00000183876

SHC2	SHC (Src homology 2 domain containing) transforming protein 2	25759	ENSG00000129946
CRADD	CASP2 and RIPK1 domain containing adaptor with death domain	8738	ENSG00000169372
LOC100506497	uncharacterized LOC100506497	100506497	NA
CGREF1	cell growth regulator with EF-hand domain 1	10669	ENSG00000138028
WDR81	WD repeat domain 81	124997	ENSG00000167716
PHACTR4	phosphatase and actin regulator 4	65979	ENSG00000204138
ACYP1	acylphosphatase 1, erythrocyte (common) type	97	ENSG00000119640
HIST1H4B	histone cluster 1, H4b	8366	ENSG00000124529
ZNF610	zinc finger protein 610	162963	ENSG00000167554
PRKCSH	protein kinase C substrate 80K-H	5589	ENSG00000130175
IGHMBP2	immunoglobulin mu binding protein 2	3508	ENSG00000132740
PRDM11	PR domain containing 11	56981	ENSG00000019485
COPB2	coatomer protein complex, subunit beta 2 (beta prime)	9276	ENSG00000184432
RPL26	ribosomal protein L26	6154	ENSG00000161970
LOC100499489	uncharacterized LOC100499489	100499489	NA
PRKAA2	protein kinase, AMP-activated, alpha 2 catalytic subunit	5563	ENSG00000162409
BRI3BP	BRI3 binding protein	140707	ENSG00000184992
SNORD116-1	small nucleolar RNA, C/D box 116-1	100033413	NA
AP1S3	adaptor-related protein complex 1, sigma 3 subunit	130340	ENSG00000152056
DUSP19	dual specificity phosphatase 19	142679	ENSG00000162999
ASB4	ankyrin repeat and SOCS box containing 4	51666	ENSG00000005981
LMO7	LIM domain 7	4008	ENSG00000136153
RHOD	ras homolog family member D	29984	ENSG00000173156
COL4A2	collagen, type IV, alpha 2	1284	ENSG00000134871
BBS2	Bardet-Biedl syndrome 2	583	ENSG00000125124
PGAM5	phosphoglycerate mutase family member 5	192111	ENSG00000247077
TCF7L1	transcription factor 7-like 1 (T-cell specific, HMG-box)	83439	ENSG00000152284
SLC12A7	solute carrier family 12 (potassium/chloride transporters), member 7	10723	ENSG00000113504
FBXO21	F-box protein 21	23014	ENSG00000135108
THAP4	THAP domain containing 4	51078	ENSG00000176946
C7orf10	chromosome 7 open reading frame 10	79783	ENSG00000175600
EPPK1	epiplakin 1	83481	NA
GRB10	growth factor receptor-bound protein 10	2887	ENSG00000106070
HOXD8	homeobox D8	3234	ENSG00000175879
TTC40	tetratricopeptide repeat domain 40	54777	ENSG00000171811
CHCHD3	coiled-coil-helix-coiled-coil-helix domain containing 3	54927	ENSG00000106554
FOXP2	forkhead box P2	93986	ENSG00000128573
PPP2R4	protein phosphatase 2A activator, regulatory subunit 4	5524	ENSG00000119383
LOC100287314	uncharacterized LOC100287314	100287314	NA
LINC00309	long intergenic non-protein coding RNA 309	150992	NA
NUCKS1	nuclear casein kinase and cyclin-dependent kinase substrate 1	64710	ENSG00000069275
FBXO34	F-box protein 34	55030	ENSG00000178974
HNRNPD	heterogeneous nuclear ribonucleoprotein D (AU-rich element RNA binding protein 1, 37kDa)	3184	ENSG00000138668
TRABD	TraB domain containing	80305	ENSG00000170638
SHCBP1	SHC SH2-domain binding protein 1	79801	ENSG00000171241
TSPAN15	tetraspanin 15	23555	ENSG00000099282
OR51A2	olfactory receptor, family 51, subfamily A, member 2	401667	ENSG00000205496
DLG2	discs, large homolog 2 (Drosophila)	1740	ENSG00000150672
LALBA	lactalbumin, alpha-	3906	ENSG00000167531
EBAG9	estrogen receptor binding site associated, antigen, 9	9166	ENSG00000147654
HEATR5A	HEAT repeat containing 5A	25938	ENSG00000129493
NXN	nucleoredoxin	64359	ENSG00000167693
TMEM51	transmembrane protein 51	55092	ENSG00000171729
PRKCZ	protein kinase C, zeta	5590	ENSG00000067606
PDE2A	phosphodiesterase 2A, cGMP-stimulated	5138	ENSG00000186642

DSC3	desmocollin 3	1825	ENSG00000134762
URGCP	upregulator of cell proliferation	55665	ENSG00000106608
MARCKS	myristoylated alanine-rich protein kinase C substrate	4082	ENSG00000155130
SACS	spastic ataxia of Charlevoix-Saguenay (sacsin)	26278	ENSG00000151835
AFF2	AF4/FMR2 family, member 2	2334	ENSG00000155966
YAE1D1	Yae1 domain containing 1	57002	ENSG00000241127
TELO2	TEL2, telomere maintenance 2, homolog (S. cerevisiae)	9894	ENSG00000100726
LAYN	layilin	143903	ENSG00000204381
GPR179	G protein-coupled receptor 179	440435	ENSG00000188888
PLXNA2	plexin A2	5362	ENSG00000076356
NRN1	neuritin 1	51299	ENSG00000124785
FAM19A5	family with sequence similarity 19 (chemokine (C-C motif)-like), member A5	25817	ENSG00000219438
TPRG1	tumor protein p63 regulated 1	285386	ENSG00000188001
LEFTY2	left-right determination factor 2	7044	ENSG00000143768
SCFD2	sec1 family domain containing 2	152579	ENSG00000184178
FLJ43860	FLJ43860 protein	389690	ENSG00000226807
RORA	RAR-related orphan receptor A	6095	ENSG00000069667
IL17RD	interleukin 17 receptor D	54756	ENSG00000144730
ALOX15B	arachidonate 15-lipoxygenase, type B	247	ENSG00000179593
CD59	CD59 molecule, complement regulatory protein	966	ENSG00000085063
ZNF365	zinc finger protein 365	22891	ENSG00000138311
TRAPPC12	trafficking protein particle complex 12	51112	ENSG00000171853
SGK223	homolog of rat pragma of Rnd2	157285	ENSG00000182319
ANKMY1	ankyrin repeat and MYND domain containing 1	51281	ENSG00000144504
ARHGAP18	Rho GTPase activating protein 18	93663	ENSG00000146376
C7orf50	chromosome 7 open reading frame 50	84310	ENSG00000146540
BBS9	Bardet-Biedl syndrome 9	27241	ENSG00000122507
SLC12A3	solute carrier family 12 (sodium/chloride transporters), member 3	6559	ENSG00000070915
PTPRN2	protein tyrosine phosphatase, receptor type, N polypeptide 2	5799	ENSG00000155093
CACNB4	calcium channel, voltage-dependent, beta 4 subunit	785	ENSG00000182389
LINC00674	long intergenic non-protein coding RNA 674	100499466	NA
MPZ	myelin protein zero	4359	ENSG00000158887
MYOM2	myomesin (M-protein) 2, 165kDa	9172	ENSG00000036448
CHM	choroideremia (Rab escort protein 1)	1121	ENSG00000188419
INSM1	insulinoma-associated 1	3642	ENSG00000173404
TG	thyroglobulin	7038	ENSG00000042832
HDAC4	histone deacetylase 4	9759	ENSG00000068024
RNASE13	ribonuclease, RNase A family, 13 (non-active)	440163	ENSG00000206150
ELP5	elongator acetyltransferase complex subunit 5	23587	ENSG00000170291
MRPL3	mitochondrial ribosomal protein L3	11222	ENSG00000114686
DGKG	diacylglycerol kinase, gamma 90kDa	1608	ENSG00000058866
BMPR2	bone morphogenetic protein receptor, type II (serine/threonine kinase)	659	ENSG00000204217
RFPL3	ret finger protein-like 3	10738	ENSG00000128276
KLF7	Kruppel-like factor 7 (ubiquitous)	8609	ENSG00000118263
ADCYAP1	adenylate cyclase activating polypeptide 1 (pituitary)	116	ENSG00000141433
FOXN3	forkhead box N3	1112	ENSG00000053254
PFKFB2	6-phosphofructo-2-kinase/fructose-2,6-biphosphatase 2	5208	ENSG00000123836
MIER2	mesoderm induction early response 1, family member 2	54531	ENSG00000105556
IFI6	interferon, alpha-inducible protein 6	2537	ENSG00000126709
MAPK6	mitogen-activated protein kinase 6	5597	ENSG00000069956
MAP2K5	mitogen-activated protein kinase kinase 5	5607	ENSG00000137764
AMFR	autocrine motility factor receptor, E3 ubiquitin protein ligase	267	ENSG00000159461
CNPY3	canopy 3 homolog (zebrafish)	10695	ENSG00000137161
SRXN1	sulfiredoxin 1	140809	ENSG00000172070
RNFT1	ring finger protein, transmembrane 1	51136	ENSG00000189050
HN1L	hematological and neurological expressed 1-like	90861	ENSG00000206053
ISLR	immunoglobulin superfamily containing leucine-rich repeat	3671	ENSG00000129009
LRRK1	leucine-rich repeat kinase 1	79705	ENSG00000154237
CLIC4	chloride intracellular channel 4	25932	ENSG00000169504

DDRGK1	DDRGK domain containing 1	65992	ENSG00000198171
TLE3	transducin-like enhancer of split 3 (E(sp1) homolog, Drosophila)	7090	ENSG00000140332
FRMD1	FERM domain containing 1	79981	ENSG00000153303
TSKS	testis-specific serine kinase substrate	60385	ENSG00000126467
HOMER2	homer homolog 2 (Drosophila)	9455	ENSG00000103942
LRP1	low density lipoprotein receptor-related protein 1	4035	ENSG00000123384
API5	apoptosis inhibitor 5	8539	ENSG00000166181
DSCAM	Down syndrome cell adhesion molecule	1826	ENSG00000171587
THUMPD3	THUMP domain containing 3	25917	ENSG00000134077
ZNF16	zinc finger protein 16	7564	ENSG00000170631
RFT1	RFT1 homolog (S. cerevisiae)	91869	ENSG00000163933
RIN3	Ras and Rab interactor 3	79890	ENSG00000100599
CFI	complement factor I	3426	ENSG00000205403
SUV39H1	suppressor of variegation 3-9 homolog 1 (Drosophila)	6839	ENSG00000101945
SLX4	SLX4 structure-specific endonuclease subunit homolog (S. cerevisiae)	84464	ENSG00000188827
USP46	ubiquitin specific peptidase 46	64854	ENSG00000109189
NAGLU	N-acetylglucosaminidase, alpha	4669	ENSG00000108784
PAX6	paired box 6	5080	ENSG00000007372
SCG2	secretogranin II	7857	ENSG00000171951
DHX8	DEAH (Asp-Glu-Ala-His) box polypeptide 8	1659	ENSG00000067596
ZC3HC1	zinc finger, C3HC-type containing 1	51530	ENSG00000091732
MMP14	matrix metallopeptidase 14 (membrane-inserted)	4323	ENSG00000157227
SORBS1	sorbin and SH3 domain containing 1	10580	ENSG00000095637
KCNK9	potassium channel, subfamily K, member 9	51305	ENSG00000169427
C3orf77	chromosome 3 open reading frame 77	375337	ENSG00000173769
SLC15A1	solute carrier family 15 (oligopeptide transporter), member 1	6564	ENSG00000088386
CALHM1	calcium homeostasis modulator 1	255022	ENSG00000185933
IL37	interleukin 37	27178	ENSG00000125571
BLVRA	biliverdin reductase A	644	ENSG00000106605
HNRNPF	heterogeneous nuclear ribonucleoprotein F	3185	ENSG00000169813
LRRC23	leucine rich repeat containing 23	10233	ENSG00000010626
ABCA6	ATP-binding cassette, sub-family A (ABC1), member 6	23460	ENSG00000154262
GALNT6	UDP-N-acetyl-alpha-D-galactosamine:polypeptide N-acetylgalactosaminyltransferase 6 (GalNAc-T6)	11226	ENSG00000139629
BRAT1	BRCA1-associated ATM activator 1	221927	ENSG00000106009
POTEC	POTE ankyrin domain family, member C	388468	ENSG00000183206
BOK	BCL2-related ovarian killer	666	ENSG00000176720
CDK2AP1	cyclin-dependent kinase 2 associated protein 1	8099	ENSG00000111328
SNAP23	synaptosomal-associated protein, 23kDa	8773	ENSG00000092531
NDRG1	N-myc downstream regulated 1	10397	ENSG00000104419
LOC100128822	uncharacterized LOC100128822	100128822	NA
TFDP1	transcription factor Dp-1	7027	ENSG00000198176
ADAMTS15	ADAM metallopeptidase with thrombospondin type 1 motif, 15	170689	ENSG00000166106
MIR4519	microRNA 4519	100616231	NA
GBE1	glucan (1,4-alpha-), branching enzyme 1	2632	ENSG00000114480
CLASP1	cytoplasmic linker associated protein 1	23332	ENSG00000074054
KCNAB2	potassium voltage-gated channel, shaker-related subfamily, beta member 2	8514	ENSG00000069424
SOD1	superoxide dismutase 1, soluble	6647	ENSG00000142168
B4GALNT3	beta-1,4-N-acetyl-galactosaminyl transferase 3	283358	ENSG00000139044
GSTK1	glutathione S-transferase kappa 1	373156	ENSG00000197448
SPTLC2	serine palmitoyltransferase, long chain base subunit 2	9517	ENSG00000100596
PACS1	phosphofurin acidic cluster sorting protein 1	55690	ENSG00000175115
MYO15A	myosin XVA	51168	ENSG00000091536
BOLL	bol, boule-like (Drosophila)	66037	ENSG00000152430
ODF3L1	outer dense fiber of sperm tails 3-like 1	161753	ENSG00000182950
MAGI2	membrane associated guanylate kinase, WW and PDZ domain containing 2	9863	ENSG00000187391
GPR161	G protein-coupled receptor 161	23432	ENSG00000143147

LRFN1	leucine rich repeat and fibronectin type III domain containing 1	57622	ENSG00000128011
MAG	myelin associated glycoprotein	4099	ENSG00000105695
MAP3K2	mitogen-activated protein kinase kinase kinase 2	10746	ENSG00000169967
CEP97	centrosomal protein 97kDa	79598	ENSG00000182504
ACSBG1	acyl-CoA synthetase bubblegum family member 1	23205	ENSG00000103740
C16orf91	chromosome 16 open reading frame 91	283951	ENSG00000174109
JMY	junction mediating and regulatory protein, p53 cofactor	133746	ENSG00000152409
HTT	huntingtin	3064	ENSG00000197386
C2orf54	chromosome 2 open reading frame 54	79919	ENSG00000172478
TMEM175	transmembrane protein 175	84286	ENSG00000127419
REEP5	receptor accessory protein 5	7905	ENSG00000129625
TRIM17	tripartite motif containing 17	51127	ENSG00000162931
LOC255025	uncharacterized LOC255025	255025	NA
RAB3D	RAB3D, member RAS oncogene family	9545	ENSG00000105514
BRI3	brain protein I3	25798	ENSG00000164713
SDK1	sidekick cell adhesion molecule 1	221935	ENSG00000146555
PRC1	protein regulator of cytokinesis 1	9055	ENSG00000198901
ZNF844	zinc finger protein 844	284391	ENSG00000223547
C16orf90	chromosome 16 open reading frame 90	646174	ENSG00000215131
TSC22D2	TSC22 domain family, member 2	9819	ENSG00000196428
C6orf222	chromosome 6 open reading frame 222	389384	ENSG00000189325
CDX4	caudal type homeobox 4	1046	ENSG00000131264
ASPDH	aspartate dehydrogenase domain containing	554235	ENSG00000204653
MCM9	minichromosome maintenance complex component 9	254394	ENSG00000111877
CLCN1	chloride channel, voltage-sensitive 1	1180	ENSG00000188037
MYT1L	myelin transcription factor 1-like	23040	ENSG00000186487
ACSM1	acyl-CoA synthetase medium-chain family member 1	116285	ENSG00000166743
CYS1	cystin 1	192668	ENSG00000205795
PTPN21	protein tyrosine phosphatase, non-receptor type 21	11099	ENSG00000070778
ANKFY1	ankyrin repeat and FYVE domain containing 1	51479	ENSG00000185722
SLC4A9	solute carrier family 4, sodium bicarbonate cotransporter, member 9	83697	ENSG00000113073
CLDN14	claudin 14	23562	ENSG00000159261
ZNF69	zinc finger protein 69	7620	ENSG00000198429
CREG1	cellular repressor of E1A-stimulated genes 1	8804	ENSG00000143162
ZNF773	zinc finger protein 773	374928	ENSG00000152439
SORCS3	sortilin-related VPS10 domain containing receptor 3	22986	ENSG00000156395
FNDC5	fibronectin type III domain containing 5	252995	ENSG00000160097
PDP2	pyruvate dehyrogenase phosphatase catalytic subunit 2	57546	ENSG00000172840
NAT16	N-acetyltransferase 16 (GCN5-related, putative)	375607	ENSG00000167011
PUS7	pseudouridylate synthase 7 homolog (S. cerevisiae)	54517	ENSG00000091127
APOOL	apolipoprotein O-like	139322	ENSG00000155008
SMARCA4	SWI/SNF related, matrix associated, actin dependent regulator of chromatin, subfamily a, member 4	6597	ENSG00000127616
WNT7A	wingless-type MMTV integration site family, member 7A	7476	ENSG00000154764
HCG4B	HLA complex group 4B (non-protein coding)	80868	NA
TSHZ3	teashirt zinc finger homeobox 3	57616	ENSG00000121297
ASIC2	acid-sensing (proton-gated) ion channel 2	40	ENSG00000108684
ARMC9	armadillo repeat containing 9	80210	ENSG00000135931
POU6F2	POU class 6 homeobox 2	11281	ENSG00000106536
KIAA0146	KIAA0146	23514	ENSG00000164808
NFE2L3	nuclear factor (erythroid-derived 2)-like 3	9603	ENSG00000050344
SPOCK2	sparc/osteonectin, cwcv and kazal-like domains proteoglycan (testican) 2	9806	ENSG00000107742
PSD4	pleckstrin and Sec7 domain containing 4	23550	ENSG00000125637
LINGO2	leucine rich repeat and Ig domain containing 2	158038	ENSG00000174482
ZBTB9	zinc finger and BTB domain containing 9	221504	ENSG00000213588
CLPTM1L	CLPTM1-like	81037	ENSG00000049656
HOXC10	homeobox C10	3226	ENSG00000180818
ITPKB	inositol-trisphosphate 3-kinase B	3707	ENSG00000143772
BLCAP	bladder cancer associated protein	10904	ENSG00000166619

TTL	tubulin tyrosine ligase	150465	ENSG00000114999
SGTA	small glutamine-rich tetratricopeptide repeat (TPR)-containing, alpha	6449	ENSG00000104969
TTC39B	tetratricopeptide repeat domain 39B	158219	ENSG00000155158
MED1	mediator complex subunit 1	5469	ENSG00000125686
EDDM3A	epididymal protein 3A	10876	ENSG00000181562
PRPF8	PRP8 pre-mRNA processing factor 8 homolog (S. cerevisiae)	10594	ENSG00000174231
EPHA6	EPH receptor A6	285220	ENSG00000080224
PPP2R1A	protein phosphatase 2, regulatory subunit A, alpha	5518	ENSG00000105568
LOC100144604	uncharacterized LOC100144604	100144604	NA
NTN1	netrin 1	9423	ENSG00000065320
HNRNPM	heterogeneous nuclear ribonucleoprotein M	4670	ENSG00000099783
PPP4R1L	protein phosphatase 4, regulatory subunit 1-like	55370	NA
GLIS3	GLIS family zinc finger 3	169792	ENSG00000107249
CCDC105	coiled-coil domain containing 105	126402	ENSG00000160994
MSX1	msh homeobox 1	4487	ENSG00000163132
ARFIP1	ADP-ribosylation factor interacting protein 1	27236	ENSG00000164144
TCERG1	transcription elongation regulator 1	10915	ENSG00000113649
PLSCR1	phospholipid scramblase 1	5359	ENSG00000188313
MICB	MHC class I polypeptide-related sequence B	4277	ENSG00000204516
ELOVL6	ELOVL fatty acid elongase 6	79071	ENSG00000170522
ST8SIA3	ST8 alpha-N-acetyl-neuraminide alpha-2,8-sialyltransferase 3	51046	ENSG00000177511
COG5	component of oligomeric golgi complex 5	10466	ENSG00000164597
UBXN11	UBX domain protein 11	91544	ENSG00000158062
TRPV4	transient receptor potential cation channel, subfamily V, member 4	59341	ENSG00000111199
PLEKHG4B	pleckstrin homology domain containing, family G (with RhoGef domain) member 4B	153478	NA
CTPS1	CTP synthase 1	1503	ENSG00000171793
DIRC3	disrupted in renal carcinoma 3	729582	NA
KLHL12	kelch-like 12 (Drosophila)	59349	ENSG00000117153
EHD4	EH-domain containing 4	30844	ENSG00000103966
DDX11L9	DEAD/H (Asp-Glu-Ala-Asp/His) box helicase 11 like 9	100288486	NA
SCARA3	scavenger receptor class A, member 3	51435	ENSG00000168077
LDB2	LIM domain binding 2	9079	ENSG00000169744
SLC27A4	solute carrier family 27 (fatty acid transporter), member 4	10999	ENSG00000167114
NECAB1	N-terminal EF-hand calcium binding protein 1	64168	ENSG00000123119
MICAL2	microtubule associated monoxygenase, calponin and LIM domain containing 2	9645	ENSG00000133816
PPIF	peptidylprolyl isomerase F	10105	ENSG00000108179
MRPS23	mitochondrial ribosomal protein S23	51649	ENSG00000181610
JSRP1	junctional sarcoplasmic reticulum protein 1	126306	ENSG00000167476
WNT11	wingless-type MMTV integration site family, member 11	7481	ENSG00000085741
FAR1	fatty acyl CoA reductase 1	84188	ENSG00000197601
STARD4	StAR-related lipid transfer (START) domain containing 4	134429	ENSG00000164211
SCN1B	sodium channel, voltage-gated, type I, beta subunit	6324	ENSG00000105711
ACVR1	activin A receptor, type I	90	ENSG00000115170
ABAT	4-aminobutyrate aminotransferase	18	ENSG00000183044
THBS1	thrombospondin 1	7057	ENSG00000137801
VAMP5	vesicle-associated membrane protein 5 (myobrevin)	10791	ENSG00000168899
TSNARE1	t-SNARE domain containing 1	203062	ENSG00000171045
TMEM35	transmembrane protein 35	59353	ENSG00000126950
BRSK2	BR serine/threonine kinase 2	9024	ENSG00000174672
TNFRSF1B	tumor necrosis factor receptor superfamily, member 1B	7133	ENSG00000028137
POU4F1-AS1	POU4F1 antisense RNA 1	100874222	NA
HELLS	helicase, lymphoid-specific	3070	ENSG00000119969
ZFYVE28	zinc finger, FYVE domain containing 28	57732	ENSG00000159733
FANCI	Fanconi anemia, complementation group I	55215	ENSG00000140525
LGALS9	lectin, galactoside-binding, soluble, 9	3965	ENSG00000168961
ABL1	c-abl oncogene 1, non-receptor tyrosine kinase	25	ENSG00000097007

PTPRG	protein tyrosine phosphatase, receptor type, G	5793	ENSG00000144724
JARID2	jumonji, AT rich interactive domain 2	3720	ENSG00000008083
PSMA7	proteasome (prosome, macropain) subunit, alpha type, 7	5688	ENSG00000101182
APLP2	amyloid beta (A4) precursor-like protein 2	334	ENSG00000084234
ELOF1	elongation factor 1 homolog (S. cerevisiae)	84337	ENSG00000130165
DPF1	D4, zinc and double PHD fingers family 1	8193	ENSG00000011332
PATL2	protein associated with topoisomerase II homolog 2 (yeast)	197135	ENSG00000229474
MC2R	melanocortin 2 receptor (adrenocorticotropic hormone)	4158	ENSG00000185231
CCDC91	coiled-coil domain containing 91	55297	ENSG00000123106
CELSR1	cadherin, EGF LAG seven-pass G-type receptor 1 (flamingo homolog, Drosophila)	9620	ENSG00000075275
CHRFAM7A	CHRNA7 (cholinergic receptor, nicotinic, alpha 7, exons 5-10) and FAM7A (family with sequence similarity 7A, exons A-E) fusion	89832	ENSG00000166664
CRIP2	cysteine-rich protein 2	1397	ENSG00000182809
CWC22	CWC22 spliceosome-associated protein homolog (S. cerevisiae)	57703	ENSG00000163510
THBS2	thrombospondin 2	7058	ENSG00000186340
CALN1	calneuron 1	83698	ENSG00000183166
GRID2	glutamate receptor, ionotropic, delta 2	2895	ENSG00000152208
ZNF423	zinc finger protein 423	23090	ENSG00000102935
HLA-G	major histocompatibility complex, class I, G	3135	ENSG00000204632
FAM184A	family with sequence similarity 184, member A	79632	ENSG00000111879
MAP3K12	mitogen-activated protein kinase kinase kinase 12	7786	ENSG00000139625
TGOLN2	trans-golgi network protein 2	10618	ENSG00000152291
FAM172A	family with sequence similarity 172, member A	83989	ENSG00000113391
SLC24A4	solute carrier family 24 (sodium/potassium/calcium exchanger), member 4	123041	ENSG00000140090
CHST7	carbohydrate (N-acetylglucosamine 6-O) sulfotransferase 7	56548	ENSG00000147119
ZNF121	zinc finger protein 121	7675	ENSG00000197961
PIGS	phosphatidylinositol glycan anchor biosynthesis, class S	94005	ENSG00000087111
MIR557	microRNA 557	693142	NA
TTYH2	tweety homolog 2 (Drosophila)	94015	ENSG00000141540
GJD2	gap junction protein, delta 2, 36kDa	57369	ENSG00000159248
GATA5	GATA binding protein 5	140628	ENSG00000130700
SPATA20	spermatogenesis associated 20	64847	ENSG00000006282
PCDH8	protocadherin 8	5100	ENSG00000136099
NOP14	NOP14 nucleolar protein homolog (yeast)	8602	ENSG00000087269
UVSSA	UV-stimulated scaffold protein A	57654	ENSG00000163945
SYT8	synaptotagmin VIII	90019	ENSG00000149043
TNIP2	TNFAIP3 interacting protein 2	79155	ENSG00000168884
C6orf10	chromosome 6 open reading frame 10	10665	ENSG00000204296
EXD3	exonuclease 3'-5' domain containing 3	54932	ENSG00000187609
MXD3	MAX dimerization protein 3	83463	ENSG00000213347
FAH	fumarylacetoacetate hydrolase (fumarylacetoacetase)	2184	ENSG00000103876
EHMT1	euchromatic histone-lysine N-methyltransferase 1	79813	ENSG00000181090
GPR6	G protein-coupled receptor 6	2830	ENSG00000146360
TTC29	tetratricopeptide repeat domain 29	83894	ENSG00000137473
VARS	valyl-tRNA synthetase	7407	ENSG00000204394
TMCO1	transmembrane and coiled-coil domains 1	54499	ENSG00000143183
RAX	retina and anterior neural fold homeobox	30062	ENSG00000134438
CDK5R1	cyclin-dependent kinase 5, regulatory subunit 1 (p35)	8851	ENSG00000176749
CTDSP1	CTD (carboxy-terminal domain, RNA polymerase II, polypeptide A) small phosphatase 1	58190	ENSG00000144579
WDR27	WD repeat domain 27	253769	ENSG00000184465
PDE6B	phosphodiesterase 6B, cGMP-specific, rod, beta	5158	ENSG00000133256
CABP2	calcium binding protein 2	51475	ENSG00000167791
TRRAP	transformation/transcription domain-associated protein	8295	ENSG00000196367
TRIM71	tripartite motif containing 71, E3 ubiquitin protein ligase	131405	ENSG00000206557
LDLRAD3	low density lipoprotein receptor class A domain containing 3	143458	ENSG00000179241
SLFN13	schlafen family member 13	146857	ENSG00000154760
SLC26A9	solute carrier family 26, member 9	115019	ENSG00000174502

C10orf129	chromosome 10 open reading frame 129	142827	ENSG00000173124
SPTBN2	spectrin, beta, non-erythrocytic 2	6712	ENSG00000173898
SLC36A2	solute carrier family 36 (proton/amino acid symporter), member 2	153201	ENSG00000186335
C17orf98	chromosome 17 open reading frame 98	388381	ENSG00000214556
CRAMP1L	Crm, cramped-like (Drosophila)	57585	ENSG00000007545
KIAA0895	KIAA0895	23366	ENSG00000164542
MMP17	matrix metallopeptidase 17 (membrane-inserted)	4326	ENSG00000198598
OXER1	oxoeicosanoid (OXE) receptor 1	165140	ENSG00000162881
TRIP4	thyroid hormone receptor interactor 4	9325	ENSG00000103671
CPLX2	complexin 2	10814	ENSG00000145920
ATXN1L	ataxin 1-like	342371	ENSG00000224470
CDC73	cell division cycle 73, Paf1/RNA polymerase II complex component, homolog (S. cerevisiae)	79577	ENSG00000134371
FAM76A	family with sequence similarity 76, member A	199870	ENSG00000009780
LINC00466	long intergenic non-protein coding RNA 466	199899	NA
HDAC11	histone deacetylase 11	79885	ENSG00000163517
KLF14	Kruppel-like factor 14	136259	ENSG00000174595
TBX20	T-box 20	57057	ENSG00000164532
NSMCE1	non-SMC element 1 homolog (S. cerevisiae)	197370	ENSG00000169189
GOLIM4	golgi integral membrane protein 4	27333	ENSG00000173905
PPP4C	protein phosphatase 4, catalytic subunit	5531	ENSG00000149923
SLC47A2	solute carrier family 47, member 2	146802	ENSG00000180638
EN2	engrailed homeobox 2	2020	ENSG00000164778
INHBE	inhibin, beta E	83729	ENSG00000139269
TP53I11	tumor protein p53 inducible protein 11	9537	ENSG00000175274
CCDC85C	coiled-coil domain containing 85C	317762	ENSG00000205476
TMEM200B	transmembrane protein 200B	399474	ENSG00000253304
NTNG1	netrin G1	22854	ENSG00000162631
ARHGAP44	Rho GTPase activating protein 44	9912	ENSG00000006740
TFAP2A	transcription factor AP-2 alpha (activating enhancer binding protein 2 alpha)	7020	ENSG00000137203
EIF4E3	eukaryotic translation initiation factor 4E family member 3	317649	ENSG00000163412
GNG7	guanine nucleotide binding protein (G protein), gamma 7	2788	ENSG00000176533
PLIN5	perilipin 5	440503	ENSG00000214456
PDE4B	phosphodiesterase 4B, cAMP-specific	5142	ENSG00000184588
STL	six-twelve leukemia	7955	NA
ZNF507	zinc finger protein 507	22847	ENSG00000168813
BRE	brain and reproductive organ-expressed (TNFRSF1A modulator)	9577	ENSG00000158019
LRP12	low density lipoprotein receptor-related protein 12	29967	ENSG00000147650
LRRC8B	leucine rich repeat containing 8 family, member B	23507	ENSG00000197147
IL1RN	interleukin 1 receptor antagonist	3557	ENSG00000136689
ODC1	ornithine decarboxylase 1	4953	ENSG00000115758
RPA3	replication protein A3, 14kDa	6119	ENSG00000106399
NOS1AP	nitric oxide synthase 1 (neuronal) adaptor protein	9722	ENSG00000198929
LOC728613	programmed cell death 6 pseudogene	728613	NA
KLHDC5	kelch domain containing 5	57542	ENSG00000087448
PDE11A	phosphodiesterase 11A	50940	ENSG00000128655
PABPN1L	poly(A) binding protein, nuclear 1-like (cytoplasmic)	390748	ENSG00000205022
ADIPOR1	adiponectin receptor 1	51094	ENSG00000159346
CCZ1	CCZ1 vacuolar protein trafficking and biogenesis associated homolog (S. cerevisiae)	51622	ENSG00000122674
KLHL29	kelch-like 29 (Drosophila)	114818	ENSG00000119771
SLITRK2	SLIT and NTRK-like family, member 2	84631	ENSG00000185985
SYBU	syntabulin (syntaxin-interacting)	55638	ENSG00000147642
SGCD	sarcoglycan, delta (35kDa dystrophin-associated glycoprotein)	6444	ENSG00000170624
RNF214	ring finger protein 214	257160	ENSG00000167257
HKR1	HKR1, GLI-Kruppel zinc finger family member	284459	ENSG00000181666
SMTN	smoothelin	6525	ENSG00000183963
DDX17	DEAD (Asp-Glu-Ala-Asp) box helicase 17	10521	ENSG00000100201
HIP1R	huntingtin interacting protein 1 related	9026	ENSG00000130787

FAM123A	family with sequence similarity 123A	219287	ENSG00000165566
CA3	carbonic anhydrase III, muscle specific	761	ENSG00000164879
MSRA	methionine sulfoxide reductase A	4482	ENSG00000175806
LYNX1	Ly6/neurotoxin 1	66004	ENSG00000180155
LOC100507377	uncharacterized LOC100507377	100507377	NA
NT5C3	5'-nucleotidase, cytosolic III	51251	ENSG00000122643
GABRB3	gamma-aminobutyric acid (GABA) A receptor, beta 3	2562	ENSG00000166206
DET1	de-etiolated homolog 1 (Arabidopsis)	55070	ENSG00000140543
PRKAR1B	protein kinase, cAMP-dependent, regulatory, type I, beta	5575	ENSG00000188191
SLC30A5	solute carrier family 30 (zinc transporter), member 5	64924	ENSG00000145740
UBE2Z	ubiquitin-conjugating enzyme E2Z	65264	ENSG00000159202
RAP2A	RAP2A, member of RAS oncogene family	5911	ENSG00000125249
KRTAP8-1	keratin associated protein 8-1	337879	ENSG00000183640
SLC35C1	solute carrier family 35, member C1	55343	ENSG00000181830
DEPTOR	DEP domain containing MTOR-interacting protein	64798	ENSG00000155792
SERPINE1	serpin peptidase inhibitor, clade E (nexin, plasminogen activator inhibitor type 1), member 1	5054	ENSG00000106366
COL4A1	collagen, type IV, alpha 1	1282	ENSG00000187498
CCDC88C	coiled-coil domain containing 88C	440193	ENSG00000015133
C20orf20	chromosome 20 open reading frame 20	55257	ENSG00000101189
DSCR4	Down syndrome critical region gene 4	10281	ENSG00000184029
MAP1B	microtubule-associated protein 1B	4131	ENSG00000131711
GRM7	glutamate receptor, metabotropic 7	2917	ENSG00000196277
SLC39A8	solute carrier family 39 (zinc transporter), member 8	64116	ENSG00000138821
TSLP	thymic stromal lymphopoietin	85480	ENSG00000145777
FBXL16	F-box and leucine-rich repeat protein 16	146330	ENSG00000127585
DENND1C	DENN/MADD domain containing 1C	79958	ENSG00000205744
RBMS3	RNA binding motif, single stranded interacting protein 3	27303	ENSG00000144642
MGC32805	uncharacterized LOC153163	153163	NA
RNF139	ring finger protein 139	11236	ENSG00000170881
BTNL2	butyrophilin-like 2 (MHC class II associated)	56244	ENSG00000204290
TGIF1	TGFB-induced factor homeobox 1	7050	ENSG00000177426
C7orf31	chromosome 7 open reading frame 31	136895	ENSG00000153790
CSNK1G3	casein kinase 1, gamma 3	1456	ENSG00000151292
NOL9	nucleolar protein 9	79707	ENSG00000162408
MNT	MAX binding protein	4335	ENSG00000070444
ENTPD1	ectonucleoside triphosphate diphosphohydrolase 1	953	ENSG00000138185
RSAD2	radical S-adenosyl methionine domain containing 2	91543	ENSG00000134321
SHANK2	SH3 and multiple ankyrin repeat domains 2	22941	ENSG00000162105
CTBP2	C-terminal binding protein 2	1488	ENSG00000175029
RAB40C	RAB40C, member RAS oncogene family	57799	ENSG00000197562
ABP1	amiloride binding protein 1 (amine oxidase (copper-containing))	26	ENSG00000002726
GDE1	glycerophosphodiester phosphodiesterase 1	51573	ENSG00000006007
INSR	insulin receptor	3643	ENSG00000171105
CDH23	cadherin-related 23	64072	ENSG00000107736
INTS4	integrator complex subunit 4	92105	ENSG00000149262
VASP	vasodilator-stimulated phosphoprotein	7408	ENSG00000125753
DTHD1	death domain containing 1	401124	ENSG00000197057
LOX	lysyl oxidase	4015	ENSG00000113083
BAG5	BCL2-associated athanogene 5	9529	ENSG00000166170
SLN	sarcolipin	6588	ENSG00000170290
PHF13	PHD finger protein 13	148479	ENSG00000116273
LOC440970	uncharacterized LOC440970	440970	NA
RPS27L	ribosomal protein S27-like	51065	ENSG00000185088
PBXIP1	pre-B-cell leukemia homeobox interacting protein 1	57326	ENSG00000163346
HLA-DRB1	major histocompatibility complex, class II, DR beta 1	3123	ENSG00000196126
MEG3	maternally expressed 3 (non-protein coding)	55384	NA
TMEM219	transmembrane protein 219	124446	ENSG00000149932
EHD1	EH-domain containing 1	10938	ENSG00000110047

ILDR2	immunoglobulin-like domain containing receptor 2	387597	ENSG00000143195
CIT	citron (rho-interacting, serine/threonine kinase 21)	11113	ENSG00000122966
BTBD19	BTB (POZ) domain containing 19	149478	ENSG00000222009
AMOTL1	angiomotin like 1	154810	ENSG00000166025
TMEM229B	transmembrane protein 229B	161145	ENSG00000198133
HERC4	HECT and RLD domain containing E3 ubiquitin protein ligase 4	26091	ENSG00000148634
ZNF615	zinc finger protein 615	284370	ENSG00000197619
SPSB4	splA/ryanodine receptor domain and SOCS box containing 4	92369	ENSG00000175093
ZNF768	zinc finger protein 768	79724	ENSG00000169957
DYRK1A	dual-specificity tyrosine-(Y)-phosphorylation regulated kinase 1A	1859	ENSG00000157540
OR51D1	olfactory receptor, family 51, subfamily D, member 1	390038	ENSG00000197428
MIR146B	microRNA 146b	574447	NA
PARD6G-AS1	PARD6G antisense RNA 1	100130522	NA
PADI6	peptidyl arginine deiminase, type VI	353238	NA
ADAMTSL5	ADAMTS-like 5	339366	ENSG00000185761
LOC283867	uncharacterized LOC283867	283867	NA
RPS6KA1	ribosomal protein S6 kinase, 90kDa, polypeptide 1	6195	ENSG00000117676
SLC7A1	solute carrier family 7 (cationic amino acid transporter, y+ system), member 1	6541	ENSG00000139514
ARHGEF17	Rho guanine nucleotide exchange factor (GEF) 17	9828	ENSG00000110237
TCF7L2	transcription factor 7-like 2 (T-cell specific, HMG-box)	6934	ENSG00000148737
ARRDC5	arrestin domain containing 5	645432	ENSG00000205784
WSB2	WD repeat and SOCS box containing 2	55884	ENSG00000176871
RNASET2	ribonuclease T2	8635	ENSG00000026297,ENSG00000249141
MORN4	MORN repeat containing 4	118812	ENSG00000171160
MORC4	MORC family CW-type zinc finger 4	79710	ENSG00000133131
C7orf25	chromosome 7 open reading frame 25	79020	ENSG00000136197
LOC100507387	uncharacterized LOC100507387	100507387	NA
CXCL5	chemokine (C-X-C motif) ligand 5	6374	ENSG00000163735
SETD7	SET domain containing (lysine methyltransferase) 7	80854	ENSG00000145391
CDV3	CDV3 homolog (mouse)	55573	ENSG00000091527
ZNF800	zinc finger protein 800	168850	ENSG00000048405
LINGO4	leucine rich repeat and Ig domain containing 4	339398	ENSG00000213171
NUDCD3	NudC domain containing 3	23386	ENSG00000015676
ACSL6	acyl-CoA synthetase long-chain family member 6	23305	ENSG00000164398
MAD2L1BP	MAD2L1 binding protein	9587	ENSG00000124688
ERMN	ermin, ERM-like protein	57471	ENSG00000136541
SYCN	syncollin	342898	ENSG00000179751
MBP	myelin basic protein	4155	ENSG00000197971
TRIML1	tripartite motif family-like 1	339976	ENSG00000184108
GCNT3	glucosaminyl (N-acetyl) transferase 3, mucin type	9245	ENSG00000140297
GPANK1	G patch domain and ankyrin repeats 1	7918	ENSG00000204438
ZMIZ1	zinc finger, MIZ-type containing 1	57178	ENSG00000108175
DESI2	desumoylating isopeptidase 2	51029	ENSG00000121644
GABRA5	gamma-aminobutyric acid (GABA) A receptor, alpha 5	2558	ENSG00000186297
CHERP	calcium homeostasis endoplasmic reticulum protein	10523	ENSG00000085872
CFLAR	CASP8 and FADD-like apoptosis regulator	8837	ENSG00000003402
ADIPOR2	adiponectin receptor 2	79602	ENSG00000006831
IGBP1	immunoglobulin (CD79A) binding protein 1	3476	ENSG00000089289
ENPP6	ectonucleotide pyrophosphatase/phosphodiesterase 6	133121	ENSG00000164303
TTC23	tetratricopeptide repeat domain 23	64927	ENSG00000103852
CDC42BPB	CDC42 binding protein kinase beta (DMPK-like)	9578	ENSG00000198752
RIMS2	regulating synaptic membrane exocytosis 2	9699	ENSG00000176406
TRAK1	trafficking protein, kinesin binding 1	22906	ENSG00000182606
MCM6	minichromosome maintenance complex component 6	4175	ENSG00000076003
RNMTL1	RNA methyltransferase like 1	55178	ENSG00000171861

AGPAT5	1-acylglycerol-3-phosphate O-acyltransferase 5 (lysophosphatidic acid acyltransferase, epsilon)	55326	ENSG00000155189
ZC3H10	zinc finger CCCH-type containing 10	84872	ENSG00000135482
SHROOM2	shroom family member 2	357	ENSG00000146950
LSM2	LSM2 homolog, U6 small nuclear RNA associated (S. cerevisiae)	57819	ENSG00000204392
COL6A3	collagen, type VI, alpha 3	1293	ENSG00000163359
RAB39A	RAB39A, member RAS oncogene family	54734	ENSG00000179331
ARHGAP36	Rho GTPase activating protein 36	158763	ENSG00000147256
GPR125	G protein-coupled receptor 125	166647	ENSG00000152990
VPS52	vacuolar protein sorting 52 homolog (S. cerevisiae)	6293	ENSG00000223501
NT5C3L	5'-nucleotidase, cytosolic III-like	115024	ENSG00000141698
DLC1	deleted in liver cancer 1	10395	ENSG00000164741
FAM178B	family with sequence similarity 178, member B	51252	ENSG00000168754
KLHL5	kelch-like 5 (Drosophila)	51088	ENSG00000109790
LAP3	leucine aminopeptidase 3	51056	ENSG00000002549
PKI55	DKFZp434H1419	150967	NA
RGMA	RGM domain family, member A	56963	ENSG00000182175
NMU	neuromedin U	10874	ENSG00000109255
KCNC2	potassium voltage-gated channel, Shaw-related subfamily, member 2	3747	ENSG00000166006
SNX8	sorting nexin 8	29886	ENSG00000106266
KCTD16	potassium channel tetramerisation domain containing 16	57528	ENSG00000183775
RSRC2	arginine/serine-rich coiled-coil 2	65117	ENSG00000111011
WDR37	WD repeat domain 37	22884	ENSG00000047056
PITX3	paired-like homeodomain 3	5309	ENSG00000107859
PTX4	pentraxin 4, long	390667	ENSG00000251692
PON2	paraoxonase 2	5445	ENSG00000105854
RALGAPA1	Ral GTPase activating protein, alpha subunit 1 (catalytic)	253959	ENSG00000174373
GRM4	glutamate receptor, metabotropic 4	2914	ENSG00000124493
STX1B	syntaxin 1B	112755	ENSG00000099365
LINC00114	long intergenic non-protein coding RNA 114	400866	NA
ASTN1	astrotactin 1	460	ENSG00000152092
GPR63	G protein-coupled receptor 63	81491	ENSG00000112218
JAKMIP3	Janus kinase and microtubule interacting protein 3	282973	ENSG00000188385
ERI3	ERI1 exoribonuclease family member 3	79033	ENSG00000117419
MAP2K1	mitogen-activated protein kinase kinase 1	5604	ENSG00000169032
MICALL2	MICAL-like 2	79778	ENSG00000164877
ANK3	ankyrin 3, node of Ranvier (ankyrin G)	288	ENSG00000151150
GSTA5	glutathione S-transferase alpha 5	221357	ENSG00000182793
OCSTAMP	osteoclast stimulatory transmembrane protein	128506	ENSG00000149635
TIMP2	TIMP metallopeptidase inhibitor 2	7077	ENSG00000035862
HIVEP3	human immunodeficiency virus type I enhancer binding protein 3	59269	ENSG00000127124
KDM2B	lysine (K)-specific demethylase 2B	84678	ENSG00000089094
SYVN1	synovial apoptosis inhibitor 1, synoviolin	84447	ENSG00000162298
PDE8B	phosphodiesterase 8B	8622	ENSG00000113231
LPCAT3	lysophosphatidylcholine acyltransferase 3	10162	ENSG00000111684
NUDT5	nudix (nucleoside diphosphate linked moiety X)-type motif 5	11164	ENSG00000165609
CDKL5	cyclin-dependent kinase-like 5	6792	ENSG00000008086
UPF3B	UPF3 regulator of nonsense transcripts homolog B (yeast)	65109	ENSG00000125351
SNX24	sorting nexin 24	28966	ENSG00000064652
PSMC3	proteasome (prosome, macropain) 26S subunit, ATPase, 3	5702	ENSG00000165916
UBP1	upstream binding protein 1 (LBP-1a)	7342	ENSG00000153560
WIPF1	WAS/WASL interacting protein family, member 1	7456	ENSG00000115935
TPPP	tubulin polymerization promoting protein	11076	ENSG00000171368
IGF2R	insulin-like growth factor 2 receptor	3482	ENSG00000197081
UST	uronyl-2-sulfotransferase	10090	ENSG00000111962
TLL1	tolloid-like 1	7092	ENSG00000038295
ATP8A2	ATPase, aminophospholipid transporter, class I, type 8A, member 2	51761	ENSG00000132932
VIPAS39	VPS33B interacting protein, apical-basolateral polarity regulator, spe-39 homolog	63894	ENSG00000151445
CLDND2	claudin domain containing 2	125875	ENSG00000160318

ADAMTS10	ADAM metallopeptidase with thrombospondin type 1 motif, 10	81794	ENSG00000142303
CALD1	caldesmon 1	800	ENSG00000122786
ALKBH8	alkB, alkylation repair homolog 8 (E. coli)	91801	ENSG00000137760
FAM82A1	family with sequence similarity 82, member A1	151393	ENSG00000115841
ERICH1	glutamate-rich 1	157697	ENSG00000104714
ARHGEF25	Rho guanine nucleotide exchange factor (GEF) 25	115557	ENSG00000240771
STMN4	stathmin-like 4	81551	ENSG00000015592
BRF2	BRF2, subunit of RNA polymerase III transcription initiation factor, BRF1-like	55290	ENSG00000104221
BLOC1S6	biogenesis of lysosomal organelles complex-1, subunit 6, pallidin	26258	ENSG00000104164
SKIL	SKI-like oncogene	6498	ENSG00000136603
GPRC5B	G protein-coupled receptor, family C, group 5, member B	51704	ENSG00000167191
SLC24A3	solute carrier family 24 (sodium/potassium/calcium exchanger), member 3	57419	ENSG00000185052
CBR3-AS1	CBR3 antisense RNA 1	100506428	NA
F11R	F11 receptor	50848	ENSG00000158769
TFCP2	transcription factor CP2	7024	ENSG00000135457
KDELR2	KDEL (Lys-Asp-Glu-Leu) endoplasmic reticulum protein retention receptor 2	11014	ENSG00000136240
POLR3F	polymerase (RNA) III (DNA directed) polypeptide F, 39 kDa	10621	ENSG00000132664
TLE4	transducin-like enhancer of split 4 (E(sp1) homolog, Drosophila)	7091	ENSG00000106829
SV2C	synaptic vesicle glycoprotein 2C	22987	ENSG00000122012
WASH3P	WAS protein family homolog 3 pseudogene	374666	NA
GIPC1	GIPC PDZ domain containing family, member 1	10755	ENSG00000123159
PKP3	plakophilin 3	11187	ENSG00000184363
ZBTB7B	zinc finger and BTB domain containing 7B	51043	ENSG00000160685
ST3GAL5	ST3 beta-galactoside alpha-2,3-sialyltransferase 5	8869	ENSG00000115525
MARS2	methionyl-tRNA synthetase 2, mitochondrial	92935	ENSG00000247626
ITGA7	integrin, alpha 7	3679	ENSG00000135424
ZNF821	zinc finger protein 821	55565	ENSG00000102984
TSPAN4	tetraspanin 4	7106	ENSG00000214063
GAK	cyclin G associated kinase	2580	ENSG00000178950
ZFAND2A	zinc finger, AN1-type domain 2A	90637	ENSG00000178381
TOLLIP	toll interacting protein	54472	ENSG00000078902
PPP2R5E	protein phosphatase 2, regulatory subunit B', epsilon isoform	5529	ENSG00000154001
DNAL1	dynein, axonemal, light chain 1	83544	ENSG00000119661
C12orf23	chromosome 12 open reading frame 23	90488	ENSG00000151135
C20orf43	chromosome 20 open reading frame 43	51507	ENSG00000022277
FBXL18	F-box and leucine-rich repeat protein 18	80028	ENSG00000155034
GPT2	glutamic pyruvate transaminase (alanine aminotransferase) 2	84706	ENSG00000166123
C1QA	complement component 1, q subcomponent, A chain	712	ENSG00000173372
DDR1	discoidin domain receptor tyrosine kinase 1	780	ENSG00000204580
PMEPA1	prostate transmembrane protein, androgen induced 1	56937	ENSG00000124225
DLK1	delta-like 1 homolog (Drosophila)	8788	ENSG00000185559
MAP4K4	mitogen-activated protein kinase kinase kinase kinase 4	9448	ENSG00000071054
HOXD9	homeobox D9	3235	ENSG00000128709
TMEM185A	transmembrane protein 185A	84548	ENSG00000155984
FAM47E	family with sequence similarity 47, member E	100129583	ENSG00000189157
FEZ2	fasciculation and elongation protein zeta 2 (zygin II)	9637	ENSG00000171055
UBTF	upstream binding transcription factor, RNA polymerase I	7343	ENSG00000108312
BEND3	BEN domain containing 3	57673	ENSG00000178409
KCNJ5	potassium inwardly-rectifying channel, subfamily J, member 5	3762	ENSG00000120457
OR10A5	olfactory receptor, family 10, subfamily A, member 5	144124	ENSG00000166363
TBCD	tubulin folding cofactor D	6904	ENSG00000141556
TMEM48	transmembrane protein 48	55706	ENSG00000058804
PDSS2	prenyl (decaprenyl) diphosphate synthase, subunit 2	57107	ENSG00000164494
TPH2	tryptophan hydroxylase 2	121278	ENSG00000139287
ACTN1	actinin, alpha 1	87	ENSG00000072110
AMPD2	adenosine monophosphate deaminase 2	271	ENSG00000116337
PRICKLE1	prickle homolog 1 (Drosophila)	144165	ENSG00000139174

OR10Q1	olfactory receptor, family 10, subfamily Q, member 1	219960	ENSG00000180475
NRBF2	nuclear receptor binding factor 2	29982	ENSG00000148572
LPIN1	lipin 1	23175	ENSG00000134324
FOXO3	forkhead box O3	2309	ENSG00000118689
ASCL1	achaete-scute complex homolog 1 (Drosophila)	429	ENSG00000139352
LARS2	leucyl-tRNA synthetase 2, mitochondrial	23395	ENSG00000011376
CCP110	centriolar coiled coil protein 110kDa	9738	ENSG00000103540
SRSF5	serine/arginine-rich splicing factor 5	6430	ENSG00000100650
B3GALNT2	beta-1,3-N-acetylgalactosaminyltransferase 2	148789	ENSG00000162885
WDR13	WD repeat domain 13	64743	ENSG00000101940
PHF10	PHD finger protein 10	55274	ENSG00000130024
LOXHD1	lipoxygenase homology domains 1	125336	ENSG00000167210
NXPE3	neurexophilin and PC-esterase domain family, member 3	91775	ENSG00000144815
LINC00663	long intergenic non-protein coding RNA 663	284440	NA
LINC00673	long intergenic non-protein coding RNA 673	100499467	NA
ZNF233	zinc finger protein 233	353355	ENSG00000159915
UHRF1	ubiquitin-like with PHD and ring finger domains 1	29128	ENSG00000034063
SNED1	sushi, nidogen and EGF-like domains 1	25992	ENSG00000162804
SLC25A31	solute carrier family 25 (mitochondrial carrier; adenine nucleotide translocator), member 31	83447	ENSG00000151475
EPS15L1	epidermal growth factor receptor pathway substrate 15-like 1	58513	ENSG00000127527
ENGASE	endo-beta-N-acetylglucosaminidase	64772	ENSG00000167280
KCNMB4	potassium large conductance calcium-activated channel, subfamily M, beta member 4	27345	ENSG00000135643
LOC154822	uncharacterized LOC154822	154822	NA
NMD3	NMD3 homolog (S. cerevisiae)	51068	ENSG00000169251
ING1	inhibitor of growth family, member 1	3621	ENSG00000153487
AHNAK2	AHNAK nucleoprotein 2	113146	ENSG00000185567
DNER	delta/notch-like EGF repeat containing	92737	ENSG00000187957
OLA1	Obg-like ATPase 1	29789	ENSG00000138430
FAM20C	family with sequence similarity 20, member C	56975	NA
POLR2E	polymerase (RNA) II (DNA directed) polypeptide E, 25kDa	5434	ENSG00000099817
TSHB	thyroid stimulating hormone, beta	7252	ENSG00000134200
ARHGEF19	Rho guanine nucleotide exchange factor (GEF) 19	128272	ENSG00000142632
SCN2B	sodium channel, voltage-gated, type II, beta subunit	6327	ENSG00000149575
RELN	reelin	5649	ENSG00000189056
MFHAS1	malignant fibrous histiocytoma amplified sequence 1	9258	ENSG00000147324
RASGRF2	Ras protein-specific guanine nucleotide-releasing factor 2	5924	ENSG00000113319
SPTBN4	spectrin, beta, non-erythrocytic 4	57731	ENSG00000160460
FMNL2	formin-like 2	114793	ENSG00000157827
ATCAY	ataxia, cerebellar, Cayman type	85300	ENSG00000167654
BNC2	basonuclin 2	54796	ENSG00000173068
SERBP1	SERPINE1 mRNA binding protein 1	26135	ENSG00000142864
MAGED1	melanoma antigen family D, 1	9500	ENSG00000179222
RESP18	regulated endocrine-specific protein 18 homolog (rat)	389075	ENSG00000182698
HEATR7A	HEAT repeat containing 7A	727957	ENSG00000179832
LIG3	ligase III, DNA, ATP-dependent	3980	ENSG00000005156
PARD3	par-3 partitioning defective 3 homolog (C. elegans)	56288	ENSG00000148498
ECI1	enoyl-CoA delta isomerase 1	1632	ENSG00000167969
RBP1	retinol binding protein 1, cellular	5947	ENSG00000114115
MCPH1	microcephalin 1	79648	ENSG00000147316
ONECUT2	one cut homeobox 2	9480	ENSG00000119547
AUTS2	autism susceptibility candidate 2	26053	ENSG00000158321
CAST	calpastatin	831	ENSG00000153113
LGR4	leucine-rich repeat containing G protein-coupled receptor 4	55366	ENSG00000205213
ROR1	receptor tyrosine kinase-like orphan receptor 1	4919	ENSG00000185483
KIAA1432	KIAA1432	57589	ENSG00000107036
MIR143HG	MIR143 host gene (non-protein coding)	728264	NA
FBXO32	F-box protein 32	114907	ENSG00000156804
PNN	pinin, desmosome associated protein	5411	ENSG00000100941

FANK1	fibronectin type III and ankyrin repeat domains 1	92565	ENSG00000203780
EPHB2	EPH receptor B2	2048	ENSG00000133216
SLC4A11	solute carrier family 4, sodium borate transporter, member 11	83959	ENSG00000088836
PTDSS2	phosphatidylserine synthase 2	81490	ENSG00000174915
TRNP1	TMF1-regulated nuclear protein 1	388610	ENSG00000253368
SNAI2	snail homolog 2 (Drosophila)	6591	ENSG00000019549
RASA3	RAS p21 protein activator 3	22821	ENSG00000185989
VSIG10	V-set and immunoglobulin domain containing 10	54621	ENSG00000176834
PLAUR	plasminogen activator, urokinase receptor	5329	ENSG00000011422
RUNDC3A	RUN domain containing 3A	10900	ENSG00000108309
FLT4	fms-related tyrosine kinase 4	2324	ENSG00000037280
ARHGEF10	Rho guanine nucleotide exchange factor (GEF) 10	9639	ENSG00000104728
ZNF133	zinc finger protein 133	7692	ENSG00000125846
OGDHL	oxoglutarate dehydrogenase-like	55753	ENSG00000197444
CASK	calcium/calmodulin-dependent serine protein kinase (MAGUK family)	8573	ENSG00000147044
GIT2	G protein-coupled receptor kinase interacting ArfGAP 2	9815	ENSG00000139436
AP2A2	adaptor-related protein complex 2, alpha 2 subunit	161	ENSG00000183020
CWF19L2	CWF19-like 2, cell cycle control (S. pombe)	143884	ENSG00000152404
FAM101B	family with sequence similarity 101, member B	359845	ENSG00000183688
RPS9	ribosomal protein S9	6203	ENSG00000170889
RFC5	replication factor C (activator 1) 5, 36.5kDa	5985	ENSG00000111445
HSPG2	heparan sulfate proteoglycan 2	3339	ENSG00000142798
SYTL3	synaptotagmin-like 3	94120	ENSG00000164674
LPCAT1	lysophosphatidylcholine acyltransferase 1	79888	ENSG00000153395
WAC	WW domain containing adaptor with coiled-coil	51322	ENSG00000095787
RBM28	RNA binding motif protein 28	55131	ENSG00000106344
ANKS3	ankyrin repeat and sterile alpha motif domain containing 3	124401	ENSG00000168096
ADCY9	adenylate cyclase 9	115	ENSG00000162104
SLCO4C1	solute carrier organic anion transporter family, member 4C1	353189	ENSG00000173930
PAX5	paired box 5	5079	ENSG00000196092
NTN3	netrin 3	4917	ENSG00000162068
DEFB136	defensin, beta 136	613210	ENSG00000205884
FCRL1	Fc receptor-like 1	115350	ENSG00000163534
UTP11L	UTP11-like, U3 small nucleolar ribonucleoprotein, (yeast)	51118	ENSG00000183520
LOC147093	uncharacterized LOC147093	147093	NA
TEX101	testis expressed 101	83639	ENSG00000131126
FDPS	farnesyl diphosphate synthase	2224	ENSG00000160752
OCA2	oculocutaneous albinism II	4948	ENSG00000104044
CP	ceruloplasmin (ferroxidase)	1356	ENSG00000047457
C1orf86	chromosome 1 open reading frame 86	199990	ENSG00000162585
TMEM26	transmembrane protein 26	219623	ENSG00000196932
SFRP1	secreted frizzled-related protein 1	6422	ENSG00000104332
AANAT	aralkylamine N-acetyltransferase	15	ENSG00000129673
ADCK5	aarF domain containing kinase 5	203054	ENSG00000173137
NANS	N-acetylneuraminic acid synthase	54187	ENSG00000095380
MTMR11	myotubularin related protein 11	10903	ENSG00000014914
IL1R1	interleukin 1 receptor, type I	3554	ENSG00000115594
FCHO2	FCH domain only 2	115548	ENSG00000157107
ZC3H12B	zinc finger CCCH-type containing 12B	340554	ENSG00000102053
SLC16A1	solute carrier family 16, member 1 (monocarboxylic acid transporter 1)	6566	ENSG00000155380
ADARB2	adenosine deaminase, RNA-specific, B2	105	ENSG00000185736
FLJ43663	uncharacterized LOC378805	378805	NA
GGA2	golgi-associated, gamma adaptin ear containing, ARF binding protein 2	23062	ENSG00000103365
IFI27L2	interferon, alpha-inducible protein 27-like 2	83982	ENSG00000119632
C11orf70	chromosome 11 open reading frame 70	85016	ENSG00000137691
INPP5A	inositol polyphosphate-5-phosphatase, 40kDa	3632	ENSG00000068383
LNX1	ligand of numb-protein X 1, E3 ubiquitin protein ligase	84708	ENSG00000072201
VRK1	vaccinia related kinase 1	7443	ENSG00000100749

CARKD	carbohydrate kinase domain containing	55739	ENSG00000213995
NR1D1	nuclear receptor subfamily 1, group D, member 1	9572	ENSG00000126368
ENPP3	ectonucleotide pyrophosphatase/phosphodiesterase 3	5169	ENSG00000154269
CLIC6	chloride intracellular channel 6	54102	ENSG00000159212
ARID1A	AT rich interactive domain 1A (SWI-like)	8289	ENSG00000117713
TRAP1	TNF receptor-associated protein 1	10131	ENSG00000126602
TUBB1	tubulin, beta 1 class VI	81027	ENSG00000101162
CAPZB	capping protein (actin filament) muscle Z-line, beta	832	ENSG00000077549
WDR73	WD repeat domain 73	84942	ENSG00000177082
SEMA3C	sema domain, immunoglobulin domain (Ig), short basic domain, secreted, (semaphorin) 3C	10512	ENSG00000075223
KAT6B	K(lysine) acetyltransferase 6B	23522	ENSG00000156650
LOC441009	uncharacterized LOC441009	441009	NA
ZBTB40	zinc finger and BTB domain containing 40	9923	ENSG00000184677
ARMCX2	armadillo repeat containing, X-linked 2	9823	ENSG00000184867
KAT2B	K(lysine) acetyltransferase 2B	8850	ENSG00000114166
ESPNP	espin pseudogene	284729	NA
COMT	catechol-O-methyltransferase	1312	ENSG00000093010
CHST3	carbohydrate (chondroitin 6) sulfotransferase 3	9469	ENSG00000122863
GPR123	G protein-coupled receptor 123	84435	ENSG00000197177
ZPBP	zona pellucida binding protein	11055	ENSG00000042813
RPS6KA3	ribosomal protein S6 kinase, 90kDa, polypeptide 3	6197	ENSG00000177189
CENPF	centromere protein F, 350/400kDa (mitosin)	1063	ENSG00000117724
BMP8B	bone morphogenetic protein 8b	656	ENSG00000116985
PCNT	pericentrin	5116	ENSG00000160299
ZBTB17	zinc finger and BTB domain containing 17	7709	ENSG00000116809
SLC19A2	solute carrier family 19 (thiamine transporter), member 2	10560	ENSG00000117479
NCR3LG1	natural killer cell cytotoxicity receptor 3 ligand 1	374383	ENSG00000188211
USP3	ubiquitin specific peptidase 3	9960	ENSG00000140455
ALPK2	alpha-kinase 2	115701	ENSG00000198796
ARL13A	ADP-ribosylation factor-like 13A	392509	ENSG00000174225
HDAC6	histone deacetylase 6	10013	ENSG00000094631
LOC339975	uncharacterized LOC339975	339975	NA
ART5	ADP-ribosyltransferase 5	116969	ENSG00000167311
AP1AR	adaptor-related protein complex 1 associated regulatory protein	55435	ENSG00000138660
PNP	purine nucleoside phosphorylase	4860	ENSG00000198805
MAPK8IP1	mitogen-activated protein kinase 8 interacting protein 1	9479	ENSG00000121653
MAGEA4	melanoma antigen family A, 4	4103	ENSG00000147381
ZADH2	zinc binding alcohol dehydrogenase domain containing 2	284273	ENSG00000180011
MLLT4	myeloid/lymphoid or mixed-lineage leukemia (trithorax homolog, Drosophila); translocated to, 4	4301	ENSG00000130396
PITPNM1	phosphatidylinositol transfer protein, membrane-associated 1	9600	ENSG00000110697
HECW1	HECT, C2 and WW domain containing E3 ubiquitin protein ligase 1	23072	ENSG00000002746
ZNF622	zinc finger protein 622	90441	ENSG00000173545
C10orf11	chromosome 10 open reading frame 11	83938	ENSG00000148655
HOPX	HOP homeobox	84525	ENSG00000171476
MEIS1-AS3	MEIS1 antisense RNA 3	730198	NA
ARNT2	aryl-hydrocarbon receptor nuclear translocator 2	9915	ENSG00000172379
KCTD1	potassium channel tetramerisation domain containing 1	284252	ENSG00000134504
OSBPL5	oxysterol binding protein-like 5	114879	ENSG00000021762
CBFA2T3	core-binding factor, runt domain, alpha subunit 2; translocated to, 3	863	ENSG00000129993
RNASEH1	ribonuclease H1	246243	ENSG00000171865
CTSC	cathepsin C	1075	ENSG00000109861
AQP7	aquaporin 7	364	ENSG00000165269
FZR1	fizzy/cell division cycle 20 related 1 (Drosophila)	51343	ENSG00000105325
WDSUB1	WD repeat, sterile alpha motif and U-box domain containing 1	151525	ENSG00000196151
SLC44A4	solute carrier family 44, member 4	80736	ENSG00000204385
BAIAP2L1	BAI1-associated protein 2-like 1	55971	ENSG00000006453
C12orf54	chromosome 12 open reading frame 54	121273	ENSG00000177627
ESM1	endothelial cell-specific molecule 1	11082	ENSG00000164283

ZNF833P	zinc finger protein 833, pseudogene	401898	NA

Supplementary Data 2. Gene set enrichment analysis for giDMR genes in T2D-discordant MZ twin pairs

Pathway name based on commons	#Gene	EntrezGene	Statistics
Proteoglycan syndecan-mediated signaling events	132	11099 10755 25942 891 1432 9815 30011 1390 10746 57521 1387 114907 998 57732 5791 7074 1021 5567 7709 2147 51065 5468 695 64798 4035 8997 2773 4772 4922 4953 6009 6498 7343 83439 8295 5524 3688 5054 1855 23671 4026 8850 2 9744 133746 7057 8837 7408 1046 50807 596 5292 4684 89780 7786 9475 1456 54756 6934 8851 3667 3643 3064 116985 5337 6197 5607 10580 8886 10397 6195 5604 6597 3597 2495 6095 5518 5300 4323 604 59349 6591 113 1356 2697 2249 9448 5209 10971 87 80854 1832 2260 4301 5590 2887 3911 6300 58190 23043 659 8874 2059 682 1452 2309 25 2048 5781 6422 9020 5469 10253 7091 5530 7050 79753 8573 1975 5329 6615 144100 3570 9564 925 816 2775 7027 80310 6840 57178 56963	C=1345; O=132; E=51.74; R=2.55; rawP=8.02e-23; adjP=4.29e-20
Beta1 integrin cell surface interactions	132	11099 891 25942 1432 9815 30011 1390 1387 57521 10746 114907 998 57732 7074 5791 1021 5567 7709 51065 5468 695 4035 64798 8997 4953 4922 4772 2773 6009 6498 7343 83439 8295 5524 3688 5054 1855 23671 4026 8850 2 9744 1293 133746 8837 7057 7408 1046 50807 596 5292 89780 4684 7786 9475 1456 54756 8851 6934 3667 3643 3064 3679 116985 5337 6197 10580 5607 8886 6195 10397 5604 6597 2495 3597 6095 5518 5300 604 4323 1282 6591 113 1292 2697 1356 2249 9448 10971 5209 87 80854 1832 2260 4301 5590 2887 3911 58190 6300 23043 659 8874 2059 1452 2309 25 5781 6422 9020 5469 10253 7091 7050 5530 79753 1975 7058 6615 5329 5575 144100 3570 9564 925 2775 816 7027 6840 80310 57178 56963	C=1351; O=132; E=51.97; R=2.54; rawP=1.19e-22; adjP=4.29e-20
Integrin family cell surface interactions	133	11099 50848 891 25942 1432 9815 30011 1390 1387 57521 10746 114907 998 57732 7074 5791 1021 5567 7709 51065 5468 695 4035 64798 8997 4953 4922 4772 2773 6009 6498 7343 83439 8295 5524 3688 5054 1855 23671 4026 8850 2 9744 1293 133746 8837 7057 7408 1046 50807 596 5292 89780 4684 7786 9475 1456 54756 8851 6934 3667 3643 3064 3679 116985 5337 6197 10580 5607 8886 6195 10397 5604 6597 2495 3597 6095 5518 5300 604 4323 1282 6591 113 1292 2697 1356 2249 9448 10971 5209 87 80854 1832 2260 4301 5590 2887 3911 58190 6300 23043 659 8874 2059 1452 2309 25 5781 6422 9020 5469 10253 7091 7050 5530 79753 1975 7058 5329 6615 5575 144100 3570 9564 925 2775 816 7027 6840 80310 57178 56963	C=1378; O=133; E=53.01; R=2.51; rawP=2.44e-22; adjP=5.86e-20

Endothelins	128	11099 891 25942 1432 9815 30011 1390 1387 57521 10746 114907 998 57732 7074 5791 1021 5567 7709 51065 5468 695 4035 64798 8997 4953 4922 4772 2773 6009 6498 7343 83439 8295 114 5524 3688 5054 1855 23671 4026 8850 2 9744 133746 8837 7408 1046 50807 596 5292 89780 4684 7786 9475 1456 54756 8851 6934 3667 3643 3064 116985 5337 6197 5607 10580 8886 10397 6195 5604 6597 3597 2495 6095 5518 5300 115 4323 604 6591 113 1356 2697 2249 9448 107 5209 10971 87 80854 1832 2260 4301 5590 2887 6300 58190 23043 659 8874 2059 1452 2309 25 5781 6422 9020 5469 10253 7091 5530 7050 79753 1975 5329 6615 144100 3570 9564 925 816 2775 7027 6550 6840 80310 57178 56963	C=1307; O=128; E=50.28; R=2.55; rawP=4.47e-22; adjP=8.06e-20
Syndecan-1-mediated signaling events	127	11099 891 25942 1432 9815 30011 1390 1387 57521 10746 114907 998 57732 7074 5791 1021 5567 7709 51065 5468 695 4035 64798 8997 4953 4922 4772 2773 6009 6498 7343 83439 8295 5524 3688 5054 1855 23671 4026 8850 2 9744 133746 8837 7408 1046 50807 596 5292 89780 4684 7786 9475 1456 54756 8851 6934 3667 3643 3064 116985 5337 6197 5607 10580 8886 10397 6195 5604 6597 3597 2495 6095 5518 5300 4323 604 6591 113 1356 2697 2249 9448 5209 10971 87 80854 1832 2260 4301 5590 2887 3911 6300 58190 23043 659 8874 2059 682 1452 2309 25 5781 6422 9020 5469 10253 7091 5530 7050 79753 8573 1975 5329 6615 144100 3570 9564 925 816 2775 7027 6840 80310 57178 56963	C=1300; O=127; E=50.01; R=2.54; rawP=8.10e-22; adjP=1.17e-19
VEGF and VEGFR signaling network	127	11099 891 25942 1432 9815 30011 1390 1387 57521 10746 114907 998 57732 7074 5791 1021 5567 7709 51065 5468 695 4035 64798 8997 4953 4922 4772 2773 6009 6498 7343 83439 8295 5524 3688 5054 1855 23671 8829 4026 8850 2 9744 133746 8837 7408 1046 2324 50807 596 5292 89780 4684 7786 9475 1456 54756 8851 6934 3667 3643 3064 116985 5337 6197 5607 10580 8886 10397 6195 5604 6597 3597 2495 6095 5518 5300 4323 604 6591 113 1356 2697 2249 9448 5209 10971 87 80854 1832 2260 4301 5590 2887 6300 58190 23043 659 8874 2059 1452 2309 25 5781 6422 9020 5469 10253 7091 5530 7050 79753 1975 26509 5329 6615 144100 3570 9564 925 816 2775 7027 6840 80310 57178 56963	C=1304; O=127; E=50.16; R=2.53; rawP=1.05e-21; adjP=1.26e-19
ErbB1 downstream signaling	124	11099 891 25942 1432 9815 30011 1390 1387 57521 10746 114907 998 57732 7074 5791 1021 5567 7709 51065 5468 695 4035 64798 8997 4953 4922 4772 2773 6009 6498 7343 83439 8295 5524 3688 5054 1855 23671 4026 8850 2 9744 133746 8837 7408 1046 50807 596 5292 89780 4684 7786 9475 1456 54756 8851 6934 3667 3643 3064 116985 5337 6197 5607 10580 8886 10397 6195 5604 6597 3597 2495 6095 5518 5300 4323 604 6591 113 1356 2697 2249 9448 5209 10971 87 80854 1832 2260 4301 5590 2887 6300 58190 23043 659 8874 2059 1452 2309 25 5781 6422 9020 5469 10253 7091 5530 7050 79753 1975 5329 6615 144100 3570 9564 925 816 2775 7027 6840 80310 57178 56963	C=1288; O=124; E=49.55; R=2.50; rawP=8.55e-21; adjP=2.28e-19

Class I PI3K signaling events mediated by Akt	124	11099 891 25942 1432 9815 30011 1390 1387 57521 10746 114907 998 57732 7074 5791 1021 5567 7709 51065 5468 695 4035 64798 8997 4953 4922 4772 2773 6009 6498 7343 83439 8295 5524 3688 5054 1855 23671 4026 8850 2 9744 133746 8837 7408 1046 50807 596 5292 89780 4684 7786 9475 1456 54756 8851 6934 3667 3643 3064 116985 5337 6197 5607 10580 8886 10397 6195 5604 6597 3597 2495 6095 5518 5300 4323 604 6591 113 1356 2697 2249 9448 5209 10971 87 80854 1832 2260 4301 5590 2887 6300 58190 23043 659 8874 2059 1452 2309 25 5781 6422 9020 5469 10253 7091 5530 7050 79753 1975 5329 6615 144100 3570 9564 925 816 2775 7027 6840 80310 57178 56963	C=1288; O=124; E=49.55; R=2.50; rawP=8.55e-21; adjP=2.28e-19
Signaling events mediated by VEGFR1 and VEGFR2	125	11099 891 25942 1432 9815 30011 1390 1387 57521 10746 114907 998 57732 7074 5791 1021 5567 7709 51065 5468 695 4035 64798 8997 4953 4922 4772 2773 6009 6498 7343 83439 8295 5524 3688 5054 1855 23671 4026 8850 2 9744 133746 8837 7408 1046 50807 596 5292 89780 4684 7786 9475 1456 54756 8851 6934 3667 3643 3064 116985 5337 6197 5607 10580 8886 10397 6195 5604 6597 3597 2495 6095 5518 5300 4323 604 6591 113 1356 2697 2249 9448 5209 10971 87 80854 1832 2260 4301 5590 2887 6300 58190 23043 659 8874 2059 1452 2309 25 5781 6422 9020 5469 10253 7091 5530 7050 79753 1975 26509 5329 6615 144100 3570 9564 925 816 2775 7027 6840 80310 57178 56963	C=1296; O=125; E=49.85; R=2.51; rawP=5.07e-21; adjP=2.28e-19
TRAIL signaling pathway	127	11099 891 25942 1432 9815 30011 1390 1387 57521 10746 114907 998 57732 7074 5791 1021 5567 7709 51065 5468 695 4035 64798 8997 4953 4922 4772 2773 6009 6498 7343 83439 8295 5524 3688 5054 1855 23671 4026 8850 2 9744 133746 8837 7408 1046 50807 596 5292 89780 4684 7786 9475 1456 54756 8738 8851 6934 3667 3643 3064 116985 5337 6197 5607 10580 8886 10397 6195 5604 6597 3597 2495 6095 5518 5300 4323 604 6591 113 1356 2697 2249 9448 5209 10971 87 7431 80854 1832 2260 4301 5590 2887 6300 58190 23043 659 8874 2059 1452 2309 25 7020 5781 6422 9020 5469 10253 7091 5530 7050 79753 1975 5329 6615 144100 3570 9564 925 816 2775 7027 6840 80310 57178 56963	C=1328; O=127; E=51.09; R=2.49; rawP=4.96e-21; adjP=2.28e-19

**Pathway name based on signaling**	**#Gene**	**EntrezGene**	**Statistics**
Calcium Regulation in the Cardiac Cell	26	5999 55970 6261 309 2697 8536 2788 107 5575 57369 10971 6000 2773 10804 3762 114 2775 816 115 1133 5590 784 10636 2709 5567 113	C=151; O=26; E=5.81; R=4.48; rawP=1.49e-10; adjP=2.18e-08

Insulin Signaling	26	5563 5607 10580 6195 9448 5604 8491 1432 2309 6009 25759 10746 3667 6196 3643 53358 8773 5597 5781 5590 2887 9020 7786 6197 6300 10938	C=163; O=26; E=6.27; R=4.15; rawP=8.33e-10; adjP=6.08e-08
Neural Crest Differentiation	21	4155 4487 7021 6615 6662 3975 79885 1488 10013 2260 9734 83439 9759 4359 429 3688 7020 1855 89780 2253 6591	C=123; O=21; E=4.73; R=4.44; rawP=9.91e-09; adjP=4.82e-07
Muscle cell TarBase	41	51479 7798 79602 6560 1432 255520 5139 7057 24145 1075 596 23261 84188 114793 29128 1021 10105 5530 3482 2697 79813 7447 4035 10971 64359 8289 3784 91869 6009 3667 4924 90441 7837 9759 23603 8773 5524 2580 8829 6300 58190	C=424; O=41; E=16.31; R=2.51; rawP=8.03e-08; adjP=2.93e-06
Epithelium TarBase	35	51479 140707 1284 79602 6560 1432 1075 604 84188 1021 29128 4670 10105 3482 60481 2697 4860 79813 4035 10971 64359 79751 8289 6009 90441 4924 7837 9759 23603 5524 55722 2580 8829 3234 58190	C=340; O=35; E=13.08; R=2.68; rawP=1.57e-07; adjP=3.82e-06
Lymphocyte TarBase	47	51479 140707 7798 1284 79602 51251 6560 1432 255520 5139 24145 27236 1075 604 596 84188 114793 29128 1021 4670 10105 5530 3482 60481 4860 79813 64359 10971 79751 3784 8289 91869 6009 3667 90441 4924 7837 9759 23603 8773 5524 55722 2580 8829 3234 6300 58190	C=533; O=47; E=20.50; R=2.29; rawP=1.52e-07; adjP=3.82e-06
Focal Adhesion	23	5607 1284 5604 7057 7408 998 53358 23533 1282 596 9475 10188 1292 7058 87 5649 9564 3679 5597 3688 80310 3911 6300	C=185; O=23; E=7.12; R=3.23; rawP=8.15e-07; adjP=1.40e-05
G Protein Signaling Pathways	16	55970 5137 5142 115 5567 113 5530 2788 107 5575 5151 2773 8622 2775 114 5590	C=95; O=16; E=3.65; R=4.38; rawP=6.70e-07; adjP=1.40e-05
EGF-EGFR Signaling Pathway	22	5607 6195 2059 5604 1432 30011 10746 25 6196 998 5781 10253 3985 10188 5359 2697 58513 9564 5590 5337 1175 6197	C=172; O=22; E=6.62; R=3.33; rawP=8.63e-07; adjP=1.40e-05
Wnt Signaling Pathway and Pluripotency	16	1488 5519 1387 5518 89780 7476 7481 4041 6934 2103 5529 83439 10023 5524 5590 1855	C=99; O=16; E=3.81; R=4.20; rawP=1.19e-06; adjP=1.74e-05

Pathway name based on KEGG	#Gene	EntrezGene	Statistics

MAPK signaling pathway	34	55970 5607 6195 5604 8912 785 1432 10746 6196 6788 998 9020 22800 2253 7786 8398 5567 2318 5923 5530 2249 9448 4914 51295 8491 8822 9479 2260 3310 3554 5924 784 6197 6300	C=268; O=34; E=10.31; R=3.30; rawP=1.26e-09; adjP=1.14e-07
Purine metabolism	26	271 55276 4860 5142 5137 107 2766 5151 5138 51251 122622 5139 8622 50940 953 272 114 115 5434 11164 2272 5158 56953 5169 113 10621	C=162; O=26; E=6.23; R=4.17; rawP=7.27e-10; adjP=1.14e-07
Axon guidance	22	5530 285220 10154 29984 2043 5362 4917 5361 4772 2773 1947 5364 25 2048 998 3688 10512 9423 8829 22854 9475 3985	C=129; O=22; E=4.96; R=4.43; rawP=4.54e-09; adjP=2.05e-07
Progesterone-mediated oocyte maturation	18	6195 5604 107 891 995 1432 2773 5139 6196 23533 114 115 51343 8379 6197 5567 113 6300	C=86; O=18; E=3.31; R=5.44; rawP=3.94e-09; adjP=2.05e-07
Neurotrophin signaling pathway	21	5607 6195 4914 9500 5604 10971 4804 1432 2309 25759 3667 25 6196 998 23533 53358 816 5781 596 6197 6300	C=127; O=21; E=4.89; R=4.30; rawP=1.78e-08; adjP=6.44e-07
Wnt signaling pathway	22	5530 7483 7476 144165 4041 1488 7481 1452 4772 6934 5519 1387 5529 83439 5518 10023 816 6422 1855 89780 9475 5567	C=150; O=22; E=5.77; R=3.81; rawP=7.67e-08; adjP=2.31e-06
Oocyte meiosis	18	5530 5529 6196 5518 816 6195 114 115 5604 107 10971 891 995 5567 5519 6197 6300 113	C=112; O=18; E=4.31; R=4.18; rawP=2.84e-07; adjP=6.90e-06
Melanogenesis	17	7483 7476 5604 107 7481 2773 6934 1387 83439 114 2775 816 115 1855 89780 5567 113	C=101; O=17; E=3.89; R=4.38; rawP=3.05e-07; adjP=6.90e-06
Tight junction	19	8573 154810 84552 50848 87 23562 4628 2773 5519 4301 998 5518 150084 5590 1364 9223 22800 9863 56288	C=132; O=19; E=5.08; R=3.74; rawP=7.78e-07; adjP=1.56e-05
Focal adhesion	24	2318 5923 1292 1284 7058 5604 5649 87 9564 25759 1293 7057 7408 998 23533 2324 53358 3679 3688 80310 596 1282 9475 3911	C=200; O=24; E=7.69; R=3.12; rawP=9.07e-07; adjP=1.64e-05

Spplementary Data 3. Gene set list for pathway enrichment analysis for giDMR genes in in human preadipocytes and adipocytes.

Gene Symbol	Gene Name	EntrezGene	Ensembl
MSRB1	methionine sulfoxide reductase B1	51734	ENSG00000198736
TINAG	tubulointerstitial nephritis antigen	27283	ENSG00000137251
NAA25	N(alpha)-acetyltransferase 25, NatB auxiliary subunit	80018	ENSG00000111300
STAB1	stabilin 1	23166	ENSG00000010327
ECE1	endothelin converting enzyme 1	1889	ENSG00000117298
RPTOR	regulatory associated protein of MTOR, complex 1	57521	ENSG00000141564
COG2	component of oligomeric golgi complex 2	22796	ENSG00000135775
OSMR	oncostatin M receptor	9180	ENSG00000145623
ABRA	actin-binding Rho activating protein	137735	ENSG00000174429
KIF26B	kinesin family member 26B	55083	ENSG00000162849
AGPAT2	1-acylglycerol-3-phosphate O-acyltransferase 2 (lysophosphatidic acid acyltransferase, beta)	10555	ENSG00000169692
DDAH1	dimethylarginine dimethylaminohydrolase 1	23576	ENSG00000153904
SNORA8	small nucleolar RNA, H/ACA box 8	654320	NA
CPA1	carboxypeptidase A1 (pancreatic)	1357	ENSG00000091704
HPN	hepsin	3249	ENSG00000105707
PLEKHM2	pleckstrin homology domain containing, family M (with RUN domain) member 2	23207	ENSG00000116786
AHDC1	AT hook, DNA binding motif, containing 1	27245	ENSG00000126705
CNTN1	contactin 1	1272	ENSG00000018236
CCDC80	coiled-coil domain containing 80	151887	ENSG00000091986
AOC3	amine oxidase, copper containing 3 (vascular adhesion protein 1)	8639	ENSG00000131471
PTHLH	parathyroid hormone-like hormone	5744	ENSG00000087494
TTC13	tetratricopeptide repeat domain 13	79573	ENSG00000143643
LRIG1	leucine-rich repeats and immunoglobulin-like domains 1	26018	ENSG00000144749
RNU5E-1	RNA, U5E small nuclear 1	26829	NA
THAP4	THAP domain containing 4	51078	ENSG00000176946
ERI1	exoribonuclease 1	90459	ENSG00000104626
SLC25A13	solute carrier family 25 (aspartate/glutamate carrier), member 13	10165	ENSG00000004864
NHSL1	NHS-like 1	57224	ENSG00000135540
CMYA5	cardiomyopathy associated 5	202333	ENSG00000164309
HMGN3	high mobility group nucleosomal binding domain 3	9324	ENSG00000118418
FAM195B	family with sequence similarity 195, member B	348262	ENSG00000225663
LIMS1	LIM and senescent cell antigen-like domains 1	3987	ENSG00000169756
SLC26A1	solute carrier family 26 (sulfate transporter), member 1	10861	ENSG00000145217
SRGAP3	SLIT-ROBO Rho GTPase activating protein 3	9901	ENSG00000196220
KLHDC8B	kelch domain containing 8B	200942	ENSG00000185909
TNC	tenascin C	3371	ENSG00000041982
ITGB1BP1	integrin beta 1 binding protein 1	9270	ENSG00000119185
FAM213A	family with sequence similarity 213, member A	84293	ENSG00000122378
GEMIN7	gem (nuclear organelle) associated protein 7	79760	ENSG00000142252
PHLDA3	pleckstrin homology-like domain, family A, member 3	23612	ENSG00000174307
FOXI1	forkhead box I1	2299	ENSG00000168269
LIPE	lipase, hormone-sensitive	3991	ENSG00000079435
SLC45A1	solute carrier family 45, member 1	50651	ENSG00000162426
DOK7	docking protein 7	285489	ENSG00000175920
MEST	mesoderm specific transcript homolog (mouse)	4232	ENSG00000106484
PRKAG2	protein kinase, AMP-activated, gamma 2 non-catalytic subunit	51422	ENSG00000106617
CMTM2	CKLF-like MARVEL transmembrane domain containing 2	146225	ENSG00000140932
PKD1L1	polycystic kidney disease 1 like 1	168507	ENSG00000158683
LRRC42	leucine rich repeat containing 42	115353	ENSG00000116212
PEMT	phosphatidylethanolamine N-methyltransferase	10400	ENSG00000133027
SENP6	SUMO1/sentrin specific peptidase 6	26054	ENSG00000112701
DGAT2	diacylglycerol O-acyltransferase 2	84649	ENSG00000062282

CEP112	centrosomal protein 112kDa	201134	ENSG00000154240
ACSL1	acyl-CoA synthetase long-chain family member 1	2180	ENSG00000151726
ZNF516	zinc finger protein 516	9658	ENSG00000101493
NGF	nerve growth factor (beta polypeptide)	4803	ENSG00000134259
MIR100HG	mir-100-let-7a-2 cluster host gene (non-protein coding)	399959	NA
TOMM7	translocase of outer mitochondrial membrane 7 homolog (yeast)	54543	ENSG00000196683
MGRN1	mahogunin ring finger 1, E3 ubiquitin protein ligase	23295	ENSG00000102858
WWOX	WW domain containing oxidoreductase	51741	ENSG00000186153
HTR3A	5-hydroxytryptamine (serotonin) receptor 3A, ionotropic	3359	ENSG00000166736
CASP8	caspase 8, apoptosis-related cysteine peptidase	841	ENSG00000064012
UVRAG	UV radiation resistance associated gene	7405	ENSG00000198382
IGFBP7	insulin-like growth factor binding protein 7	3490	ENSG00000163453
NAA50	N(alpha)-acetyltransferase 50, NatE catalytic subunit	80218	ENSG00000121579
DAXX	death-domain associated protein	1616	ENSG00000204209
FASN	fatty acid synthase	2194	ENSG00000169710
ITGA9	integrin, alpha 9	3680	ENSG00000144668
FAM184A	family with sequence similarity 184, member A	79632	ENSG00000111879
HMHA1	histocompatibility (minor) HA-1	23526	ENSG00000180448
PECR	peroxisomal trans-2-enoyl-CoA reductase	55825	ENSG00000115425
DUSP6	dual specificity phosphatase 6	1848	ENSG00000139318
PIEZO1	piezo-type mechanosensitive ion channel component 1	9780	ENSG00000103335
TMEM95	transmembrane protein 95	339168	ENSG00000182896
OR4C13	olfactory receptor, family 4, subfamily C, member 13	283092	ENSG00000258817
KAZN	kazrin, periplakin interacting protein	23254	ENSG00000189337
ZFP91	zinc finger protein 91 homolog (mouse)	80829	ENSG00000255073, ENSG00000186660
HIPK2	homeodomain interacting protein kinase 2	28996	ENSG00000064393
ATG16L2	autophagy related 16-like 2 (S. cerevisiae)	89849	ENSG00000168010
SCD5	stearoyl-CoA desaturase 5	79966	ENSG00000145284
CFLAR	CASP8 and FADD-like apoptosis regulator	8837	ENSG00000003402
DIP2C	DIP2 disco-interacting protein 2 homolog C (Drosophila)	22982	ENSG00000151240
BAHCC1	BAH domain and coiled-coil containing 1	57597	ENSG00000171282
EIF4EBP1	eukaryotic translation initiation factor 4E binding protein 1	1978	ENSG00000187840
GYG2	glycogenin 2	8908	ENSG00000056998
TMEM60	transmembrane protein 60	85025	ENSG00000135211
ACACB	acetyl-CoA carboxylase beta	32	ENSG00000076555
KIAA0226	KIAA0226	9711	ENSG00000145016
PC	pyruvate carboxylase	5091	ENSG00000173599
WDR33	WD repeat domain 33	55339	ENSG00000136709
CP	ceruloplasmin (ferroxidase)	1356	ENSG00000047457
CORO2B	coronin, actin binding protein, 2B	10391	ENSG00000103647
LEMD2	LEM domain containing 2	221496	ENSG00000161904
CPEB2	cytoplasmic polyadenylation element binding protein 2	132864	ENSG00000137449
BICC1	bicaudal C homolog 1 (Drosophila)	80114	ENSG00000122870
NPEPPS	aminopeptidase puromycin sensitive	9520	ENSG00000141279
DLC1	deleted in liver cancer 1	10395	ENSG00000164741
FBN3	fibrillin 3	84467	ENSG00000142449
GRK5	G protein-coupled receptor kinase 5	2869	ENSG00000198873
MACROD1	MACRO domain containing 1	28992	ENSG00000133315
AKAP8	A kinase (PRKA) anchor protein 8	10270	ENSG00000105127
ADD2	adducin 2 (beta)	119	ENSG00000075340
ADAMTS2	ADAM metallopeptidase with thrombospondin type 1 motif, 2	9509	ENSG00000087116
SRSF4	serine/arginine-rich splicing factor 4	6429	ENSG00000116350
HEBP2	heme binding protein 2	23593	ENSG00000051620
ANKRD11	ankyrin repeat domain 11	29123	ENSG00000167522
ZAK	sterile alpha motif and leucine zipper containing kinase AZK	51776	ENSG00000091436
DLG5	discs, large homolog 5 (Drosophila)	9231	ENSG00000151208
WDR73	WD repeat domain 73	84942	ENSG00000177082
ADAMTSL4	ADAMTS-like 4	54507	ENSG00000143382

MEGF6	multiple EGF-like-domains 6	1953	ENSG00000162591
MIR3134	microRNA 3134	100422990	NA
SLC35D1	solute carrier family 35 (UDP-glucuronic acid/UDP-N-acetylgalactosamine dual transporter), member D1	23169	ENSG00000116704
EGFR	epidermal growth factor receptor	1956	ENSG00000146648
RREB1	ras responsive element binding protein 1	6239	ENSG00000124782
FMNL3	formin-like 3	91010	ENSG00000161791
KDM3B	lysine (K)-specific demethylase 3B	51780	ENSG00000120733
ATXN7	ataxin 7	6314	ENSG00000163635
TOM1L2	target of myb1-like 2 (chicken)	146691	ENSG00000175662
CELF2	CUGBP, Elav-like family member 2	10659	ENSG00000048740
ZNF736	zinc finger protein 736	728927	ENSG00000234444
PKD2L1	polycystic kidney disease 2-like 1	9033	ENSG00000107593
ADTRP	androgen-dependent TFPI-regulating protein	84830	ENSG00000111863
PGBD5	piggyBac transposable element derived 5	79605	ENSG00000177614
ORMDL1	ORM1-like 1 (S. cerevisiae)	94101	ENSG00000128699
ZNF876P	zinc finger protein 876, pseudogene	642280	NA
ZCCHC14	zinc finger, CCHC domain containing 14	23174	ENSG00000140948
ATP10A	ATPase, class V, type 10A	57194	ENSG00000206190
TDP1	tyrosyl-DNA phosphodiesterase 1	55775	ENSG00000042088
CEP170	centrosomal protein 170kDa	9859	ENSG00000143702
TBC1D16	TBC1 domain family, member 16	125058	ENSG00000167291
DNAJA4	DnaJ (Hsp40) homolog, subfamily A, member 4	55466	ENSG00000140403
MIR548AE2	microRNA 548ae-2	100616339	NA
MOCS1	molybdenum cofactor synthesis 1	4337	ENSG00000124615
FBXL14	F-box and leucine-rich repeat protein 14	144699	ENSG00000171823
TRIM10	tripartite motif containing 10	10107	ENSG00000204613
USP54	ubiquitin specific peptidase 54	159195	ENSG00000166348
NR4A1	nuclear receptor subfamily 4, group A, member 1	3164	ENSG00000123358
TNNT3	troponin T type 3 (skeletal, fast)	7140	ENSG00000130595
MYO1C	myosin IC	4641	ENSG00000197879
SGPL1	sphingosine-1-phosphate lyase 1	8879	ENSG00000166224
DDX39B	DEAD (Asp-Glu-Ala-Asp) box polypeptide 39B	7919	ENSG00000198563
MLXIP	MLX interacting protein	22877	ENSG00000175727
BRF1	BRF1 homolog, subunit of RNA polymerase III transcription initiation factor IIIB (S. cerevisiae)	2972	ENSG00000185024

Supplementary Data 4. Gene set enrichment analysis for giDMR genes in human preadipocytes and adipocytes.

Pathway name based on commons	#Gene	EntrezGene	Statistics
Fatty acid, triacylglycerol, and ketone body metabolism	6	32 2180 51422 10555 2194 84649	C=83; O=6; E=0.28; R=21.65; rawP=3.81e-07; adjP=4.91e-05
Metabolism of lipids and lipoproteins	8	32 2180 51422 10555 2194 84649 3991 8879	C=258; O=8; E=0.86; R=9.29; rawP=2.75e-06; adjP=0.0001
Triglyceride Biosynthesis	4	2180 10555 2194 84649	C=28; O=4; E=0.09; R=42.78; rawP=2.29e-06; adjP=0.0001
Alpha9 beta1 integrin signaling events	15	57521 1848 1356 1616 146225 3164 1978 28996 26018 841 3680 3371 51776 1956 8837	C=1305; O=15; E=4.36; R=3.44; rawP=3.37e-05; adjP=0.0009
Activated AMPK stimulates fatty-acid oxidation in muscle	3	32 2180 51422	C=18; O=3; E=0.06; R=49.92; rawP=2.87e-05; adjP=0.0009
Arf6 signaling events	13	57521 1848 1356 1616 146225 3164 1978 28996 26018 841 51776 1956 8837	C=1288; O=13; E=4.30; R=3.02; rawP=0.0004; adjP=0.0011
S1P1 pathway	13	57521 1848 1356 1616 146225 3164 1978 28996 26018 841 51776 1956 8837	C=1288; O=13; E=4.30; R=3.02; rawP=0.0004; adjP=0.0011
PAR1-mediated thrombin signaling events	13	57521 1848 1356 1616 146225 3164 1978 28996 26018 841 51776 1956 8837	C=1299; O=13; E=4.34; R=3.00; rawP=0.0004; adjP=0.0011
Glucose metabolism	3	8908 5091 10165	C=36; O=3; E=0.12; R=24.96; rawP=0.0002; adjP=0.0011
EGFR-dependent Endothelin signaling events	13	57521 1848 1356 1616 146225 3164 1978 28996 26018 841 51776 1956 8837	C=1289; O=13; E=4.30; R=3.02; rawP=0.0004; adjP=0.0011

Pathway name based on signaling	#Gene	EntrezGene	Statistics
Fatty Acid Biosynthesis	5	32 2180 55825 5091 2194	C=29; O=5; E=0.10; R=51.64; rawP=4.31e-08; adjP=1.12e-06
AMPK signaling	6	57521 32 3991 51422 1978 2194	C=66; O=6; E=0.22; R=27.23; rawP=9.61e-08; adjP=1.25e-06
MAPK signaling pathway	7	1848 841 4803 1616 3164 51776 1956	C=165; O=7; E=0.55; R=12.71; rawP=1.49e-06; adjP=1.29e-05
Triacylglyceride Synthesis	3	3991 10555 84649	C=24; O=3; E=0.08; R=37.44; rawP=7.01e-05; adjP=0.0005

TOR signaling	3	57521 51422 1978	C=36; O=3; E=0.12; R=24.96; rawP=0.0002; adjP=0.0010
Integrated Pancreatic Cancer Pathway	5	2972 841 10555 1616 1956	C=181; O=5; E=0.60; R=8.27; rawP=0.0004; adjP=0.0017
FAS pathway and Stress induction of HSP regulation	3	841 1616 8837	C=51; O=3; E=0.17; R=17.62; rawP=0.0007; adjP=0.0026
Mitochondrial LC-Fatty Acid Beta-Oxidation	2	2180 55825	C=16; O=2; E=0.05; R=37.44; rawP=0.0013; adjP=0.0042
Interferon induced apoptosis	2	841 8837	C=20; O=2; E=0.07; R=29.95; rawP=0.0020; adjP=0.0058
Insulin Signaling	3	3991 1978 4641	C=163; O=3; E=0.54; R=5.51; rawP=0.0175; adjP=0.0455

**Pathway name based on KEGG**	**#Gene**	**EntrezGene**	**Statistics**
Insulin signaling pathway	6	57521 3991 32 51422 1978 2194	C=138; O=6; E=0.46; R=13.02; rawP=7.46e-06; adjP=0.0002
MAPK signaling pathway	6	1848 1616 4803 51776 3164 1956	C=268; O=6; E=0.89; R=6.71; rawP=0.0003; adjP=0.0026
Fatty acid biosynthesis	2	32 2194	C=6; O=2; E=0.02; R=99.83; rawP=0.0002; adjP=0.0026
Adipocytokine signaling pathway	3	32 51422 2180	C=68; O=3; E=0.23; R=13.21; rawP=0.0016; adjP=0.0104
Biosynthesis of unsaturated fatty acids	2	55825 79966	C=21; O=2; E=0.07; R=28.52; rawP=0.0022; adjP=0.0114
Apoptosis	3	841 4803 8837	C=87; O=3; E=0.29; R=10.33; rawP=0.0031; adjP=0.0134
Pyruvate metabolism	2	32 5091	C=40; O=2; E=0.13; R=14.97; rawP=0.0079; adjP=0.0293
RNA transport	3	1978 79760 7919	C=151; O=3; E=0.50; R=5.95; rawP=0.0143; adjP=0.0310
Glycerolipid metabolism	2	10555 84649	C=50; O=2; E=0.17; R=11.98; rawP=0.0122; adjP=0.0310
Metabolic pathways	9	32 8639 2180 5091 10555 10400 84649 2194 8879	C=1130; O=9; E=3.77; R=2.39; rawP=0.0139; adjP=0.0310
